# Reagent-controlled regiodivergent intermolecular cyclization of 2-aminobenzothiazoles with β-ketoesters and β-ketoamides

**DOI:** 10.3762/bjoc.13.270

**Published:** 2017-12-18

**Authors:** Irwan Iskandar Roslan, Kian-Hong Ng, Gaik-Khuan Chuah, Stephan Jaenicke

**Affiliations:** 1 Department of Chemistry, National University of Singapore, 3 Science Drive 3, Singapore 117543

**Keywords:** cyclization, fused-ring systems, indium, radical, regiodivergent

## Abstract

Two regiodivergent approaches to intermolecular cyclization of 2-aminobenzothiazoles with β-ketoesters and amides have been developed, controlled by the reagents employed. With the Brønsted base KO*t-*Bu and CBrCl_3_ as radical initiator, benzo[*d*]imidazo[2,1-*b*]thiazoles are synthesized via attack at the α-carbon and keto carbon of the β-ketoester moiety. In contrast, switching to the Lewis acid catalyst, In(OTf)_3_, results in the regioselective nucleophilic attack at both carbonyl groups forming benzo[4,5]thiazolo[3,2-*a*]pyrimidin-4-ones instead.

## Introduction

β-Ketoesters are versatile substrates frequently used in heterocyclic synthesis, having both electrophilic keto and ester moieties as well as a nucleophilic α-carbon. They can act as dinucleophiles [1–4], dielectrophiles [5–7] or ambiphiles [8–9] in the presence of their complementary coupling partners. Furthermore, they can be prefunctionalized [10–13] with leaving groups, thus switching to an electrophile [14–16], or convert to an α-radical carbon with an oxidant [17–19]. β-Ketoesters are also inexpensive, abundant and commercially available, making them attractive substrates.

In our continuing effort to develop green and atom-efficient protocols, we have employed transition-metal-free approaches for the one-pot synthesis of imidazo[1,2-*a*]pyridines and thiazolamines by coupling β-ketoesters or their derivatives, phenylacetones and phenylacetophenones, with aminopyridines [20–21] and thioureas [22]. The strategy involves in situ bromination of the α-carbon using CBrCl_3_ as the Br source. This in situ halogenation strategy has been employed for the synthesis of quinoxalines [23], oxazoles [24–25], pyrido[1,2-*a*]benzimidazoles [26], imidazo[1,2-*a*]pyridines [27–30], thiazoles [31–32] and benzothiazoles [33–34]. With weak bicarbonate bases, direct bromination of the α-carbon does not occur. Instead, the Br is shuttled to the α-carbon by its coupling partner. With this tandem bromination and cyclization strategy, there is no need to presynthesize substrates, thus reducing the number of synthetic steps, time, chemicals and wastes. Here we describe the extension of this α-bromination shuttle system to 2-aminobenzothiazoles as substrates to synthesize benzo[*d*]imidazo[2,1-*b*]thiazoles.

The benzo[*d*]imidazo[2,1-*b*]thiazole backbone is found in many bioactive molecules and pharmaceutical compounds as evident by its use as antimicrobial [35–36], antitumor [37–39], antibacterial [40], and anti-allergic agents [41]. In addition, compounds with this backbone are employed as kinase inhibitors and receptors [42–44] and as a tracer for PET imaging of β-amyloid plaques [45–46]. The conventional approach for the construction of benzo[*d*]imidazo[2,1-*b*]thiazole is the condensation of 2-aminobenzothiazole with α-halo or tosyloxy ketone [47–48]. The requirement for prior functionalization of the ketone moiety is a drawback, and several more direct methods have been developed in recent years [49–52]. Zhang et al. coupled various 1,2-dihaloarenes with 2-mercaptobenzimidazole in a nucleophilic aromatic substitution reaction [53]. Zhu’s group used Cu salts as a promoter for the cycloaddition of isocyanides with benzothiazoles [54]. Benzo[*d*]imidazo[2,1-*b*]thiazoles have also been synthesized via coupling of 2-aminobenzothiazole with acetophenones [55] or aldehydes and nitroalkanes [56].

Because the bicyclic structure of 2-aminobenzothiazole is more stable than aminopyridines and thioureas, the nucleophilicity of the aromatic N atom is reduced. A stronger base, KO*t-*Bu, is therefore needed for in situ bromination to form the benzo[*d*]imidazo[2,1-*b*]thiazole derivatives via coupling of 2-aminobenzothiazole with the brominated β-ketoesters and amides. Over the past decade, there has been a lot of interest in KO*t*-Bu-mediated synthesis, especially after Itami’s group showed that KO*t-*Bu provides a metal-free approach to the traditional Pd-catalyzed aryl–heteroaryl coupling to biaryls [57]. Since then, KO*t-*Bu has been used as a mediator for various reactions including aryl–aryl coupling [58–63], inter- and intramolecular cyclizations [64–68], amidation [69], alkenylation [70], oxidation [71] and silylation [72]. In these reactions, the single electron transfer (SET) is initiated by KO*t*-Bu/DMF [63,67,69,71] or KO*t-*Bu in combination with additives such as bidentate diamine ligands [61–65], 18-crown-6 [70] or azobisisobutyronitrile (AIBN) [62,66].

**Scheme 1:**
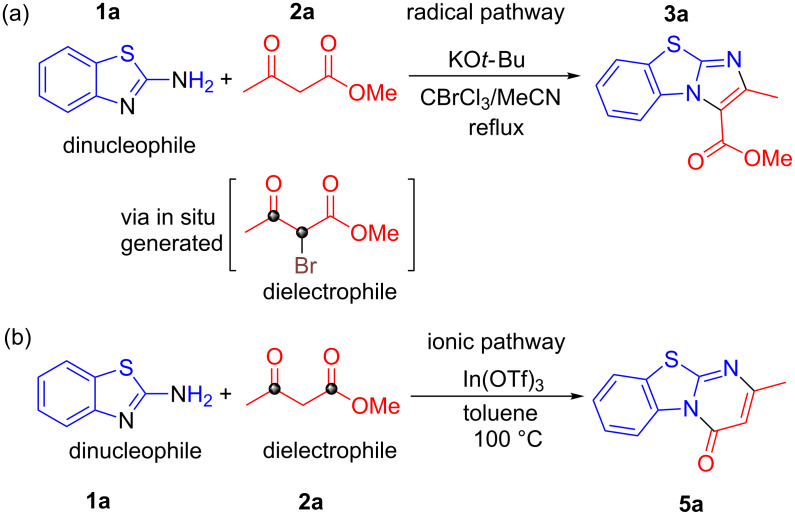
Two different intermolecular cyclization pathways controlled by reagents used.

Herein, we report the synthesis of benzo[*d*]imidazo[2,1-*b*]thiazoles via an in situ bromination strategy using a KO*t*-Bu/CBrCl_3_ system (Scheme 1a). By employing a base with radical susceptibility, the reaction mechanism is expected to differ substantially from that using bicarbonate bases which operates via the α-Br shuttle mechanism [20–22]. Interestingly, by replacing the radical initiator and Brønsted base system with a Lewis acid catalyst, benzo[4,5]thiazolo[3,2-*a*]pyrimidin-4-ones were formed instead (Scheme 1b). This highlights the versatility of β-ketoesters where the regioselectivity of the reaction is directed by the nature of the reagents.

## Results and Discussion

Optimization studies were carried out by reacting 1.2 mmol of 2-aminobenzothiazole (**1a**) with 1.0 mmol of methyl acetoacetate (**2a**) in the presence of 2 equivalents of base using a solvent mixture of CBrCl_3_/MeCN under reflux. After 16 h, the desired product **3a** was not formed with KHCO_3_ (Table 1, entry 1). Instead, the *N*-acetylated side product **4** was obtained in moderate yields, together with trace amounts of the benzo[4,5]thiazolo[3,2-*a*]pyrimidin-4-one side product **5a**. Even after switching to the stronger bases K_2_CO_3_ and K_3_PO_4_, only poor yields of **3a** were obtained (Table 1, entries 2 and 3). These results are not surprising as the substrate **1a** is a weaker dinucleophile than aminopyridine and thiourea used previously [20–22]. However, 56% yield of **3a** was obtained with KOH (Table 1, entry 4). The use of KOH as a base in the α-bromination of 1,3-dicarbonyl compounds has been reported previously by Sasson’s group [73]. We propose that with a strong base like KOH, direct α-bromination of **2a** occurs instead of relaying the Br through an α-bromination shuttle as observed in our previous studies [20–22]. Other strong bases like NaH and KOEt also proceeded with moderate yields of **3a** (Table 1, entries 5 and 6). Gratifyingly, an even higher yield of **3a**, 86%, was obtained with KO*t*-Bu (Table 1, entry 7).

**Table 1:** Optimization parameters for the synthesis of benzo[*d*]imidazo[2,1-*b*]thiazoles **3a**.^a^

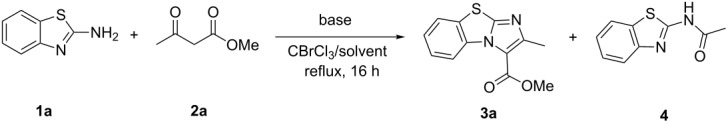				

Entry	Base	Solvent	Yield of **3a** (%)^b,c^	Yield of **4** (%)^b,c^

1	KHCO_3_	MeCN	0	40 (31)
2	K_2_CO_3_	MeCN	trace	45 (37)
3	K_3_PO_4_	MeCN	5	53 (46)
4	KOH	MeCN	56 (48)	31
5	NaH	MeCN	69 (63)	20
6	KOEt	MeCN	61 (53)	22
7	KO*t*-Bu	MeCN	86 (84)	14
8^d^	KO*t*-Bu	DMF	77 (75)	5
9^d^	KO*t*-Bu	DMAc	74 (70)	7
10	KO*t*-Bu	EtOAc	33 (22)	38
11	KO*t*-Bu	DCE	9	24
12^d^	KO*t*-Bu	toluene	trace	44 (37)
13^e^	KO*t*-Bu	MeCN	89 (86)	11
14^f^	KO*t*-Bu	MeCN	65 (58)	23
15^g^	KO*t*-Bu	MeCN	78 (74)	22
16^h^	KO*t*-Bu	MeCN	46 (35)	6

^a^Reaction conditions: **1a** (1.2 mmol), **2a** (1.0 mmol), base (2.0 equiv), in 3 mL of 1:9 (v/v) CBrCl_3_/solvent (3 mmol CBrCl_3_) for 16 h. ^b^Yield (from GC) with respect to **2a**, using biphenyl as an internal standard. ^c^Isolated yields in parenthesis. ^d^Reaction conducted at 80 °C. ^e^5.0 and ^f^2.0 mmol of CBrCl_3_ was used. ^g^2.0 mmol of **1a** used. ^h^1.0 mmol of **1a** and 2.0 mmol of **2a** used.

Next, various solvents besides MeCN were investigated. The polar aprotic solvents DMF and DMAc were suitable albeit with slightly lower yields of **3a** (Table 1, entries 8 and 9). On the other hand, ethyl acetate and 1,2-dichloroethane fared poorly whereas toluene gave only the *N*-acetylated side product **4** (Table 1, entries 10–12). Unfortunately, further attempts to increase the yields of **3a**, including increasing the equivalents of CBrCl_3_, **1a** and **2a** proved futile (Table 1, entries 13–16).

**Scheme 2:**
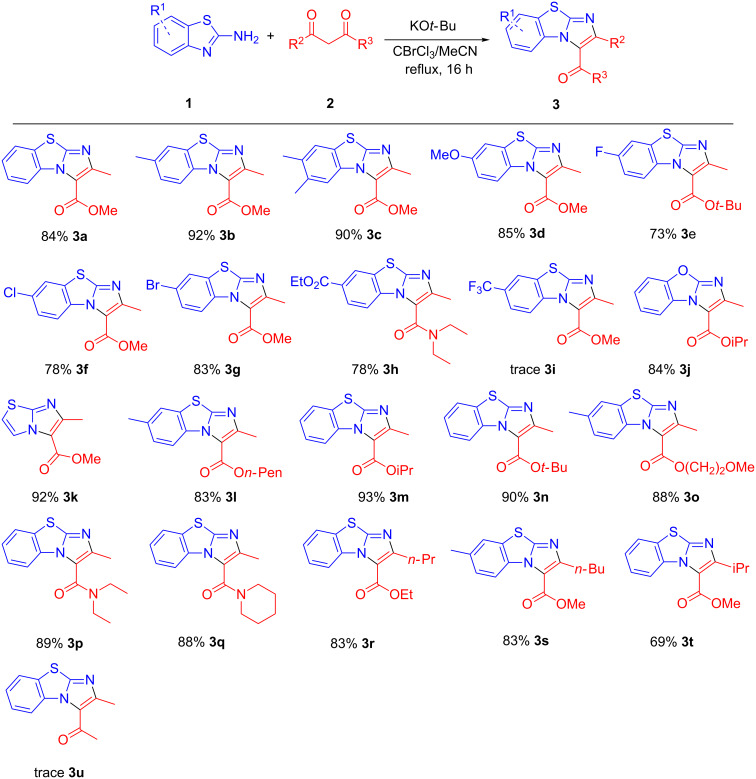
Scope of reaction. Reaction conditions: **1** (1.2 mmol), **2** (1.0 mmol), KO*t*-Bu (2 mmol), in 3 mL CBrCl_3_/MeCN 1:9 (v/v) under reflux for 16 h.

Hence, exploring the scope of the reaction was carried out using 1.0 mmol of methyl acetoacetate **2a** with 1.2 mmol of CBrCl_3_/MeCN solvent mixture at refluxing conditions for 16 h. A wide array of 2-aminobenzothiazoles was screened (Scheme 2). Good to excellent yields were obtained with 2-aminobenzothiazoles bearing electron donating methyl, dimethyl or methoxy groups (Scheme 2, **3b**–**d**). Halogen substituents F, Cl and Br were well tolerated under the optimized conditions. 2-Aminobenzothiazoles with an ester moiety also proceeded with good yields (Scheme 2, **3h**). However, only trace amounts of **3i** were obtained for 2-aminobenzothiazoles bearing the strongly electron-withdrawing CF_3_ group. It was encouraging to see both that the benzoxazole and thiazole derivatives reacted well with their respective β-ketoester coupling partners to form **3j** and **3k** with 84% and 92% yields, respectively.

**Scheme 3:**
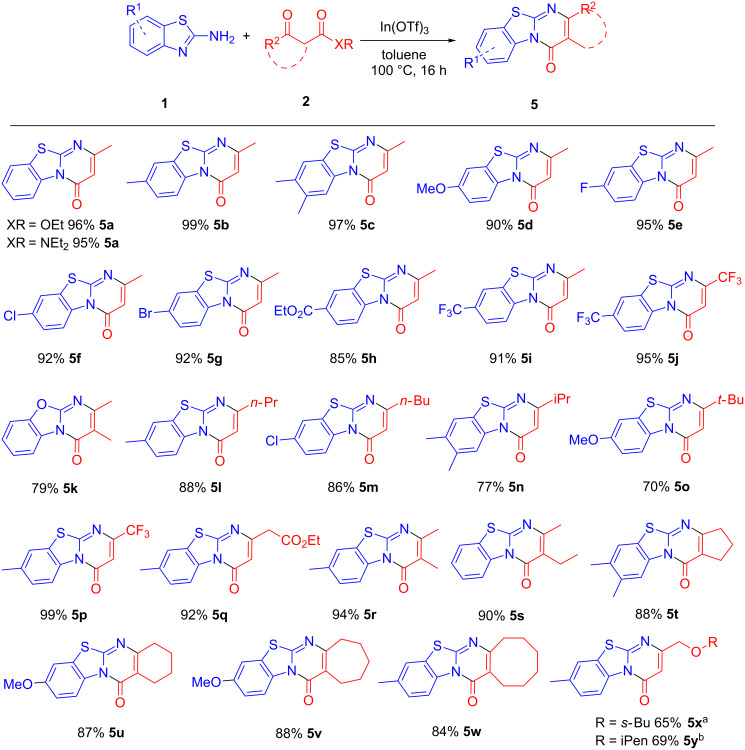
Scope of the reaction. Reaction conditions: **1** (1.0 mmol), **2** (1.5 mmol), In(OTf)_3_ (0.1 mmol), in 1.5 mL toluene at 100 °C for 16 h. ^a^2 mL of 2-butanol/toluene 1:3 (v/v) used as solvent instead. ^b^Isopentanol/toluene.

A variety of β-ketoesters and β-ketoamides were tested using the optimized conditions. Alkyl acetoacetates, including pentyl, isopropyl, *tert*-butyl and methoxyethyl, reacted smoothly with excellent yields (Scheme 2, **3l**–**3o**). β-Ketoamides were well tolerated (Scheme 2, **3h**, **3p** and **3q**) while good yields were obtained with β-ketoesters containing *n*-propyl and *n*-butyl moieties (Scheme 2, **3r** and **3s**). However, significantly lower yields were obtained with the sterically more demanding iPr group (Scheme 2, **3t**). Unfortunately, β-diketone (acetylacetone) was not suitable for the reaction as only trace amounts of **3u** were formed, with the bulk of the substrate being converted to the N-acylated side product **4** instead.

As trace amounts of benzo[4,5]thiazolo[3,2-*a*]pyrimidin-4-ones, **5a**, were observed in the synthesis of benzo[*d*]imidazo[2,1-*b*]-thiazoles, we searched for a suitable reagent to regioselectively form this instead of **3a** and **4**. This tricyclic backbone can be found in compounds that possess a wide range of medicinal properties including those with antimalarial [74], anticancer [75–76], antiallergic [77–78], antibacterial [79], and antimicrobial properties [80–81]. In addition, they have been found to be biologically active antagonists of adenosine receptors [82], inhibitors of cyclic-AMP-diphosphoesterase [83], and benzodiazepine receptor ligands [84–85]. Reported methods to access this structural motif include annulation between an aromatic amine and acid chloride [86] or via aza-Diels–Alder reaction [87]. Coupling between alkynoic acid and 2-aminobenzothiazole and the use of ionic liquids have also been developed [88]. Heterogeneous catalysts such as kaolin and hydrotalcites have been employed in the synthesis of benzo[4,5]thiazolo[3,2-*a*]pyrimidin-4-ones [89–90]. Polyphosphoric acid has also been used to access **5a** by coupling 2-aminobenzothiazole with β-ketoesters [79,91–92].

We have previously shown that bismuth salts can catalyze the intermolecular cyclization of 2-aminopyridines and β-ketoesters to pyrido[1,2-*a*]pyrimidin-4-ones [6]. Using Bi(OTf)_3_ in 1.5 mmol 2-aminobenzolthiazole (**1a**) and 1.0 mmol methyl acetoacetate (**2a**) gave a relatively poor yield of **5a** (Table 2, entry 1). This prompted us to switch the equivalents of **1a** and **2a** to 1.0 and 1.5 mmol, respectively, which improved the yield tremendously (Table 2, entry 2). Hence, the survey of Lewis acids was conducted with 10 mol % of the Lewis acid at 100 °C using toluene as solvent (Table 2). In(OTf)_3_ was a better catalyst for this reaction with 95% yield (Table 2, entry 3). Reactions with Zn^II^ and Yb^III^ triflates proceeded with moderate yields while Ag^I^, Cu^II^ and Co^II^ were unreactive (Table 2, entries 4–8). Other In^III^ salts screened were not as effective as its triflate salt (Table 2, entries 9 and 10). A wide range of solvents were also screened. Dioxane was fairly suitable for the reaction with moderate yields of **5a** whereas nitromethane fared poorly (Table 2, entries 11 and 12). Propionitrile, DMF and DMSO were also unsuitable for the reaction (Table 2, entries 13–15). Hence, establishing the scope of reaction was carried out using In(OTf)_3_ as catalyst and toluene as solvent.

**Table 2:** Optimization parameters for the synthesis of benzo[4,5]thiazolo[3,2-*a*]pyrimidin-4-ones **5a**.^a^

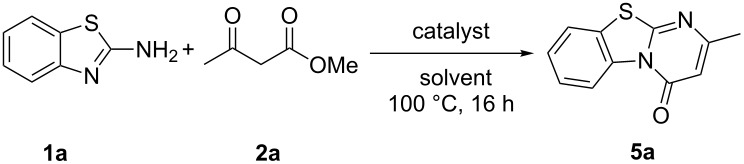			

Entry	Catalyst	Solvent	Yield of **5a**^b,c^ (%)^b,c^

1^d^	Bi(OTf)_3_	toluene	23
2	Bi(OTf)_3_	toluene	73 (70)
3	In(OTf)_3_	toluene	96 (95)
4	Yb(OTf)_3_	toluene	59 (52)
5	Zn(OTf)_2_	toluene	67 (57)
6	Cu(OTf)_2_	toluene	0
7	AgOTf	toluene	0
8	CoCl_2_	toluene	0
9	InCl_3_	toluene	63 (58)
10	InBr_3_	toluene	50 (40)
11	In(OTf)_3_	dioxane	39 (26)
12	In(OTf)_3_	NO_2_Me	10
13	In(OTf)_3_	EtCN	18
14	In(OTf)_3_	DMF	0
15	In(OTf)_3_	DMSO	0

^a^Reaction conditions: **1a** (1.0 mmol), **2a** (1.5 mmol), catalyst (10 mol %), in 1.5 mL of solvent for 16 h at 100 °C. ^b^Yield (from GC) with respect to **1a**, using biphenyl as an internal standard. ^c^Isolated yields in parenthesis. ^d^Reaction using 1.5 mmol of **1a** and 1.0 mmol of **2a** instead.

Both ethyl acetoacetate and *N*,*N*-diethylacetamide reacted smoothly with **1a** to give **5a** in good yields (Scheme 3). A large variety of substituted 2-aminobenzothiazoles were then screened in this reaction. Electron-donating groups including methyl and methoxy were well tolerated under the reaction conditions (Scheme 3, **5b**–**d**). 2-Aminobenzothiazoles with halogen substituents F, Cl and Br reacted smoothly with excellent yields (Scheme 3, **5e**–**g**). Good to excellent yields were also obtained with electron-withdrawing ester and CF_3_ moieties (Scheme 3, **5h**–**j**). Likewise, the reaction with 2-aminobenzoxazole proceeded smoothly giving **5k** in 79% yield.

Next, a variety of methyl or ethyl carboxylates of β-ketoesters were screened for this reaction. Good yields were obtained for β-ketoesters with *n*-propyl and *n*-butyl moieties at the C-4 carbon (Scheme 3, **5l** and **5m**). Bulkier isopropyl and *tert*-butyl substituents were less well tolerated, with significantly lower yields (Scheme 3, **5n** and **5o**). The best yield of 99% was obtained when ethyl 4,4,4-trifluoroacetoacetate was employed while the reaction with diethyl 3-oxopentanedioate proceeded with 92% yield (Scheme 3, **5p** and **5q**). β-Ketoesters with methyl or ethyl substituent at the α-carbon were well suited for this reaction (Scheme 3, **5r** and **5s**). Gratifyingly, the reaction also proceeded smoothly with various cyclic β-oxo esters ranging from cyclopentane to cyclooctane (Scheme 3, **5t**–**w**). Interestingly, the ethers **5x** and **5y** were obtained when ethyl 4-chloroacetoacetate was reacted with 2-amino-6-methylbenzothiazole in a mixed solvent system of toluene with 2-butanol or isopentanol, respectively. The Cl is utilized as a leaving group during the nucleophilic attack by the corresponding alcohol.

**Scheme 4:**
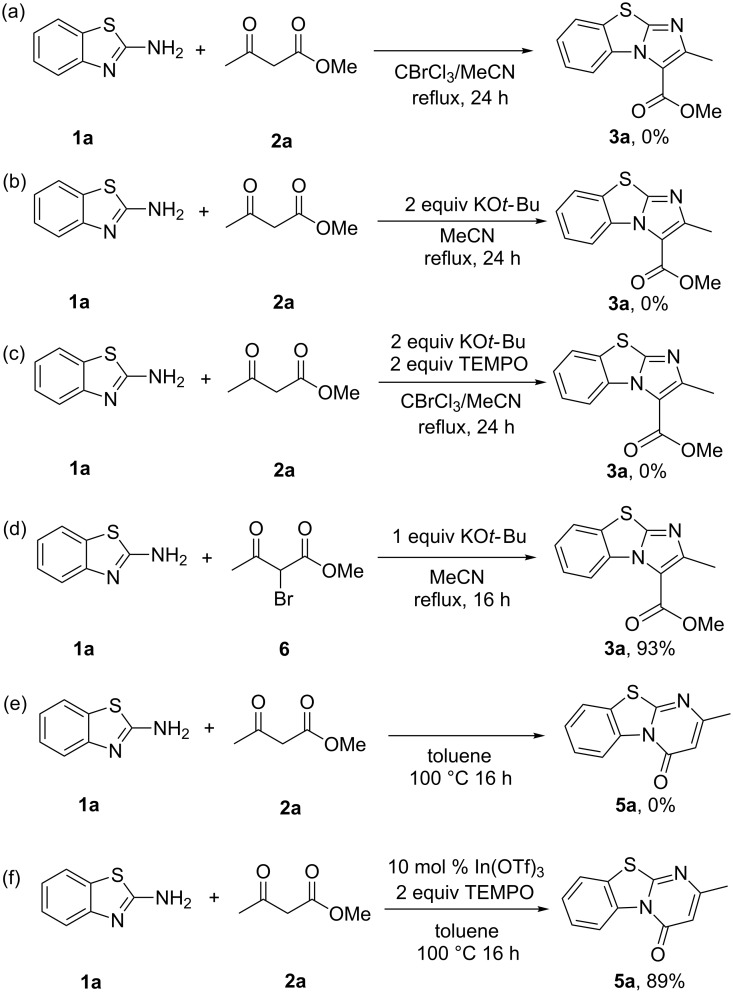
Control experiments.

A series of control experiments were conducted to gain insights into the mechanism of the two contrasting reactions. Firstly, two separate coupling reactions between 2-aminobenzothiazole (**1a**) and methyl acetoacetate (**2a**) were conducted using the optimized reaction conditions, one without KO*t*-Bu and the other without CBrCl_3_ (Scheme 4a and b). The absence of **3a** in both cases shows that both KO*t-*Bu and CBrCl_3_ have to be present to form the desired product. Next, to confirm that the reaction proceeds via a radical pathway, 2,2,6,6-tetramethylpiperidin-1-yl oxyl (TEMPO, 2.0 equiv) was added to the reaction mixture as a radical scavenger (Scheme 4c). After 24 h, none of the desired product **3a** had formed, indicating that the reaction pathway was a radical one in nature. Homolytic cleavage of the C–Br bond in CBrCl_3_ can occur under UVC irradiation or thermally [21]. The cleavage is facile and was also observed under blue LED light [93]. Finally, the α-brominated methyl acetoacetate **6** was detected in several reaction mixtures. To prove that **6** is indeed a reaction intermediate, the starting material **2a** was replaced with **6** (Scheme 4d). After 16 h, 93% of **3a** was formed, showing unambiguously that the reaction proceeds via **6** as an intermediate. We propose that KO*t*-Bu assists in α-bromination of **2a** to form the intermediate **6** via single electron transfer (SET). Recent mechanistic work by Murphy and co-workers showed that in the presence of polyhalomethane CBr_4_, KO*t*-Bu undergoes a SET reaction, forming •CBr_3_ and *t*-BuO• radicals [95]. Furthermore, with other strong bases of similar strength including NaH and KOEt the reaction proceeded with significantly lower yields. This lends additional support to the hypothesis that KO*t*-Bu, besides being a base, has a secondary role of assisting in SET (Table 1, entries 5 and 6).

The following control experiments were carried out for the synthesis of benzo[4,5]thiazolo[3,2-*a*]pyrimidin-4-ones. A blank reaction was done without the In^III^ catalyst in toluene under the optimized conditions (Scheme 4e). No desired product **5a** was formed, implying the need for In(OTf)_3_ as catalyst in the reaction. A radical trapping experiment with 2 equivalents of TEMPO was also conducted using the optimized conditions (Scheme 4f). 89% of **5a** was formed, implying that the reaction does not proceed via a radical pathway but an ionic one.

**Figure 1:**
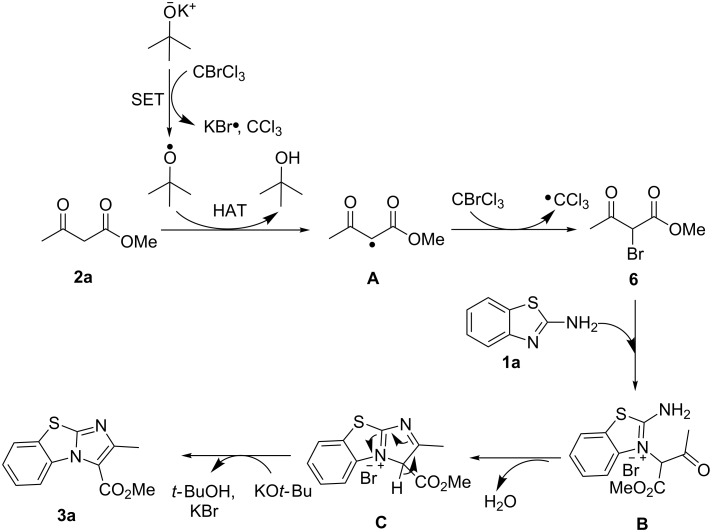
Proposed mechanism (benzo[*d*]imidazo[2,1-*b*]thiazoles).

**Figure 2:**
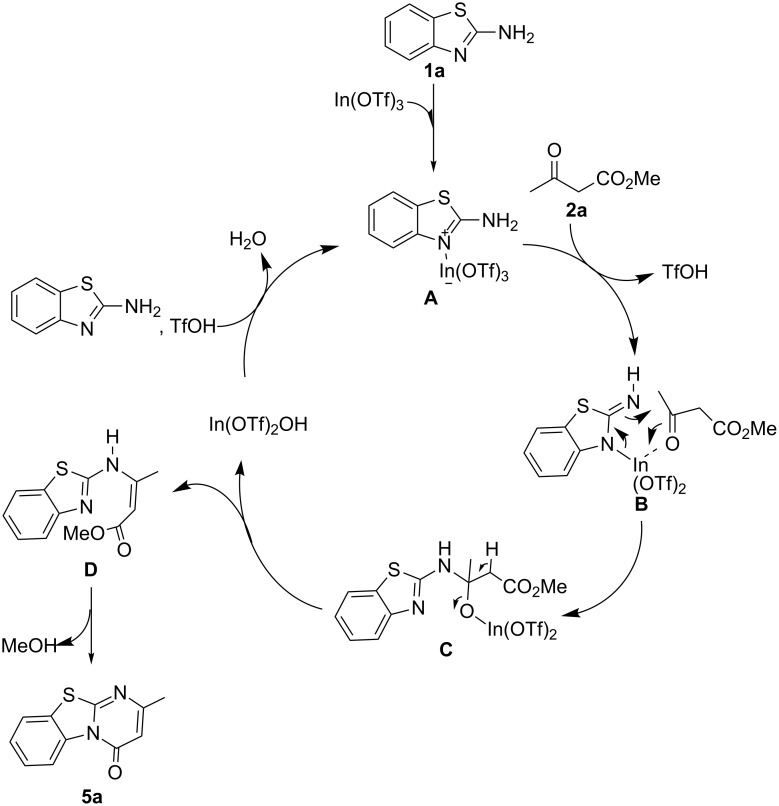
Proposed mechanism (benzo[4,5]thiazolo[3,2-*a*]pyrimidin-4-ones).

Based on these observations, the syntheses of benzo[*d*]imidazo[2,1-*b*]thiazoles and benzo[4,5]thiazolo[3,2-*a*]pyrimidin-4-ones are proposed to occur via the following mechanisms (Figure 1 and Figure 2). For the former, the reaction is initiated by SET from the *tert*-butoxide anion to CBrCl_3_, forming the *tert*-butoxy radical [94]. This radical attacks the α-hydrogen of **2a** via hydrogen atom transfer (HAT), to form intermediate **A** with a radical at the α-carbon. **A** then undergoes α-bromination to form the intermediate **6** [95]. Attack at the α-carbon of **6** by 2-aminobenzothiazole (**1a**) via an Ortoleva–King type of reaction forms **B** [96–97]. This is followed by a nucleophilic addition and dehydration to form **C**. Upon deprotonation of the acidic proton at **C** by KO*t*-Bu, the desired product **3a** is formed with release of KBr and *tert*-butanol. As proposed by Zeitler’s group, the •CCl_3_ radicals are quenched to CHCl_3_ via HAT [93].

The proposed catalytic cycle for the synthesis of benzo[4,5]thiazolo[3,2-*a*]pyrimidin-4-ones is as follows. The Lewis acidic In^III^ metal center coordinates to the more nucleophilic benzothiazole N atom, forming an adduct **A** [98]. This activates the N–H bonds and its subsequent cleavage by the triflate forms **B** [99]. The In^III^ metal center also coordinates to the keto carbon of methyl acetoacetate (**2a**) and thus brings both substrates in close proximity to one another [100–101]. An addition reaction ensues the formation of **C** which is followed by proton abstraction, releasing the catalyst as In(OTf)_2_OH [102] and forming **D**. An intramolecular condensation reaction occurs which forms the desired product **5a** with extrusion of MeOH. The In(OTf)_3_ is regenerated by TfOH with release of a water molecule, and the catalytic cycle is repeated.

## Conclusion

We have developed two regiodivergent protocols for the intermolecular cyclization of 2-aminobenzothiazoles with β-ketoesters and β-ketoamides that are determined by the reagents used. This is possible due to the versatility of β-ketoesters in switching polarities and reactivities in the presence of different reagents. With the Brønsted base and radical initiator system of KO*t-*BU/CBrCl_3_, in situ α-bromination occurs and nucleophilic attacks at the α-carbon and keto carbon lead to the formation of benzo[*d*]imidazo[2,1-*b*]thiazoles. On the other hand, the Lewis acidic catalyst In(OTf)_3_ allows for nucleophilic attacks at both carbonyl groups to form benzo[4,5]thiazolo[3,2-*a*]pyrimidin-4-ones. The scope of these regiodivergent protocols was demonstrated with 19 examples of tricyclic benzo[*d*]imidazo[2,1-*b*]thiazoles and 27 examples of tricyclic and tetracyclic benzo[4,5]thiazolo[3,2-*a*]pyrimidin-4-ones.

## Experimental

**Representative procedure for the synthesis of benzo[*d*]imidazo[2,1-*b*]thiazole:** A 25 mL two-neck round-bottomed flask was charged with 2-aminobenzothiazole (**1a**, 180 mg, 1.2 mmol), methyl acetoacetate (**2a**, 108 μL, 1.0 mmol), in 3 mL of CBrCl_3_/MeCN 1:9 (v/v) solvent mixture. KO*t*-Bu (224 mg, 2.0 mmol) was added slowly at room temperature and the reaction mixture was stirred under reflux for 16 h. Upon completion, the reaction mixture was diluted with 30 mL of ethyl acetate, filtered through a short pad of silica gel and washed down with an additional 60 mL ethyl acetate. The filtrate was washed with distilled water (3 × 30 mL) and the organic phase was dried with anhydrous Na_2_SO_4_. After filtration, the solvent was removed by rotary evaporation and the residue was purified by column chromatography using hexane and ethyl acetate (v/v = 8:1) as eluent to afford **3a** with 84% yield.

**Methyl 2-methylbenzo[*d*]imidazo[2,1-*b*]thiazole-3-carboxylate (3a):** Obtained as a yellow semi-solid (206 mg, 84%); ^1^H NMR (300 MHz, CDCl_3_) 8.95 (d, *J* = 8.1 Hz, 1H), 7.66 (d, *J* = 7.8 Hz, 1H), 7.45 (t, *J* = 8.0 Hz, 1H), 7.34 (t, *J* = 7.5 Hz, 1H), 3.97 (s, 3H), 2.63 (s, 3H); ^13^C NMR (75 MHz, CDCl_3_) 161.1, 154.4, 151.7, 134.0, 129.7,126.3, 125.0, 123.6, 118.3, 117.6, 51.6, 16.9; HRMS–ESI (*m*/*z*): [M + H]^+^: calcd for C_12_H_11_N_2_O_2_S, 247.0536; found, 247.0533.

**Representative procedure for the synthesis of benzo[*d*]imidazo[2,1-*b*]thiazole:** A 10 mL round-bottomed flask was charged with 2-aminobenzothiazole (**1a**, 150 mg, 1.0 mmol), methyl acetoacetate (**2a**, 162 μL, 1.5 mmol) and indium(III) trifluoromethanesulfonate (56 mg, 0.1 mmol) in 1.5 mL of toluene. After stirring at 100 °C for 16 h, the reaction was diluted with water and extracted with EtOAc (15 mL × 5). The combined organic layers were washed with brine and dried with anhydrous Na_2_SO_4_. After filtration, the solvent was removed by rotary evaporation, and the residue was cleaned up by column chromatography using hexane and ethyl acetate (v/v = 4:1) as eluent to afford **5a** with 95% yield.

**2-Methyl-4*H*-benzo[4,5]thiazolo[3,2-*a*]pyrimidin-4-one (5a):** Obtained as a light yellow solid (206 mg, 95%); mp 202–204 °C; ^1^H NMR (300 MHz, CDCl_3_) δ 8.94 (d, *J* = 7.5 Hz, 1H), 7.57 (d, *J* = 7.2 Hz, 1H), 7.44–7.33 (m, 2H), 6.16 (s, 1H), 2.30 (s, 3H); ^13^C NMR (75 MHz, CDCl_3_) δ 162.6, 161.1, 160.8, 135.8, 126.7, 126.6, 123.8, 121.5, 119.7, 106.9, 23.5; HRMS–ESI (*m*/*z*): [M + H]^+^ calcd for C_11_H_9_N_2_OS, 217.0430; found, 217.0432.

## Supporting Information

File 1 Experimental procedure, analytical data and NMR spectra.
