# Supplementary material for: Reagent-controlled regiodivergent intermolecular cyclization of 2-aminobenzothiazoles with β-ketoesters and β-ketoamides
Source: Beilstein J Org Chem. 2017 Dec 18;13:2739–50. doi: 10.3762/bjoc.13.270 (PMC5753174; doi:10.3762/bjoc.13.270)
Supplement: File 1 — Experimental procedure, analytical data and NMR spectra. [file Beilstein_J_Org_Chem-13-2739-s001.pdf]

Supporting Information

for

Reagent-controlled regiodivergent intermolecular cyclization of 2-aminobenzothiazoles with β-ketoesters and β-ketoamides

Irwan Iskandar Roslan, Kian-Hong Ng, Gaik-Khuan Chuah* and Stephan Jaenicke*

Address: Department of Chemistry, National University of Singapore, 3 Science Drive 3, Singapore 117543

Email: Gaik-Khuan Chuah - chmcgk@nus.edu.sg; Stephan Jaenicke - chmsj@nus.edu.sg

*Corresponding author

Experimental procedure, analytical data and NMR spectra

General information……................……………….....…………………………………….…......S2

General procedure for synthesis of benzo[*d*]imidazo[2,1-*b*]thiazole........…………………...S2

General procedure for synthesis of benzo[4,5]thiazolo[3,2-*a*]pyrimidin-4-ones……………….S2

Analytical data for products...S3

References S18

NMR spectra S19

General information

The following chemicals were obtained from Alfa-Aesar, Sigma-Aldrich, and TCI and used as received: $CBrCl_3$, KO^tBu, MeCN, $In(OTf)_3$, toluene, methyl acetoacetate and their derivatives, 2-aminobenzothiazole and their derivatives. Thin-layer chromatography (TLC) was performed using TLC silica gel 60 F_{254} glass plates. Silica gel 60 (230–400 mesh) was used for column chromatography. The ^1H NMR and ^{13}C NMR of samples in $CDCl_3$ were measured using a Bruker Avance 300 (AV300) spectrometer with tetramethylsilane as an internal standard. For ^1H NMR spectra, chemical shifts were reported in ppm (δ), multiplicity (s = singlet, d = doublet, t = triplet, q = quartet, m = multiplet, b = broad) and coupling constant (Hz). Detection of compounds by gas chromatography was performed using an Agilent 6890N gas chromatograph equipped with a HP-5 column and an FID detector. Analysis of samples by gas chromatography mass spectrometry was carried out using a Hewlett-Packard HP6890 gas chromatograph with 5973 mass selective detector. Mass spectra measurements were recorded on Bruker micrOTOFQII under electrospray ionization (ESI) mode. Melting points were recorded with samples in capillary tubes using an automated melting point system.

General procedure for the synthesis of benzo[*d*]imidazo[2,1-*b*]thiazole

A 25 mL two-neck round-bottomed flask was charged with 2-aminobenzothiazole (**1a**, 180 mg, 1.2 mmol), methyl acetoacetate (**2a**, 108 μL, 1.0 mmol), in 3 mL of $CBrCl_3$/MeCN 1:9 (v/v) solvent mixture. KO*t*-Bu (224 mg, 2.0 mmol) was added slowly at room temperature and the reaction mixture was stirred under reflux for 16 h. Upon completion, the reaction mixture was diluted with 30 mL of ethyl acetate, filtered through a short pad of silica gel and washed down with an additional 60 mL ethyl acetate. The filtrate was washed with distilled water (3 × 30 mL) and the organic phase was dried with anhydrous Na_2SO_4. After filtration, the solvent was removed by rotary evaporation and the residue was purified by column chromatography using hexane and ethyl acetate (v/v = 8:1) as eluent to afford **3a** with 84% yield.

General procedure for the synthesis of benzo[4,5]thiazolo[3,2-*a*]pyrimidin-4-ones

A 10 mL round-bottomed flask was charged with 2-aminobenzothiazole (**1a**, 150 mg, 1.0 mmol), methyl acetoacetate (**2a**, 162 μL, 1.5 mmol) and indium(III) trifluoromethanesulfonate (56 mg,

0.1 mmol) in 1.5 mL of toluene. After stirring at 100 °C for 16 h, the reaction was diluted with water and extracted with EtOAc (15 mL × 5). The combined organic layers were washed with brine and dried with anhydrous Na_2SO_4. After filtration, the solvent was removed by rotary evaporation, and the residue was cleaned up by column chromatography using hexane and ethyl acetate (v/v = 4:1) as eluent to afford **5a** with 95% yield.

Analytical Data for Products

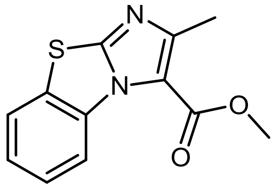

Methyl 2-methylbenzo[*d*]imidazo[2,1-*b*]thiazole-3-carboxylate (3a). Obtained as a yellow semi-solid (206 mg, 84%); ^{1}H NMR (300 MHz, $CDCl_3$) δ 8.95 (d, J = 8.1 Hz, 1H), 7.66 (d, J = 7.8 Hz, 1H), 7.45 (t, J = 8.0 Hz, 1H), 7.34 (t, J = 7.5 Hz, 1H), 3.97 (s, 3H), 2.63 (s, 3H); ^{13}C NMR (75 MHz, $CDCl_3$) δ 161.1, 154.4, 151.7, 134.0, 129.7,126.3, 125.0, 123.6, 118.3, 117.6, 51.6, 16.9. HRMS (ESI) calcd for $C_{12}H_{11}N_2O_2S$ $[M+H]^+$: 247.0536; found 247.0533.

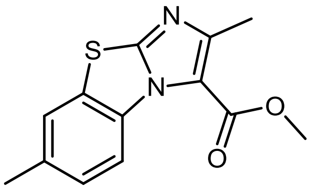

Methyl 2,7-dimethylbenzo[*d*]imidazo[2,1-*b*]thiazole-3-carboxylate (3b). Obtained as a yellow solid (240 mg, 92%); mp 145-147 °C; ^{1}H NMR (300 MHz, $CDCl_3$) δ 8.79 (d, J = 8.7 Hz, 1H), 7.44 (s, 1H), 7.23 (d, J = 8.7 Hz, 1H), 3.96 (s, 3H), 2.62 (s, 3H), 2.44 (s, 3H); ^{13}C NMR (75 MHz, $CDCl_3$) δ 161.1, 153.9, 151.5, 135.1, 132.0, 129.8, 127.3, 123.5, 118.2, 117.2, 51.5, 21.1, 16.8. HRMS (ESI) calcd for $C_{13}H_{13}N_2O_2S$ $[M+H]^+$: 261.0692; found 261.0695.

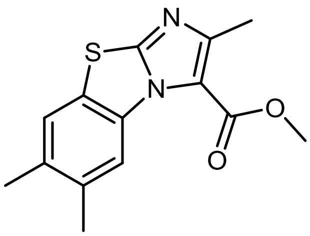

Methyl 2,6,7-trimethylbenzo[*d*]imidazo[2,1-*b*]thiazole-3-carboxylate (3c). Obtained as a yellow semi-solid (246 mg, 90%); ^{1}H NMR (75 MHz, $CDCl_3$) δ 8.70 (s, 1H), 7.38 (s, 1H), 3.97 (s, 3H), 2.62 (s, 3H), 2.39 (s, 3H), 2.33 (s, 3H); ^{13}C NMR (300 MHz, $CDCl_3$) δ 161.2, 154.0, 151.8, 135.4, 134.1, 132.4, 126.7, 123.7, 118.1, 118.0, 51.5, 20.4, 19.7, 16.9. HRMS (ESI) calcd for $C_{14}H_{15}N_2O_2S$ [M+H]$^+$: 275.0849; found 275.0850.

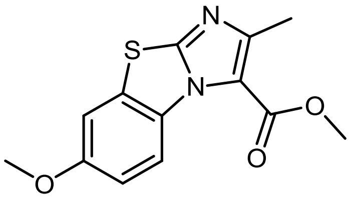

Methyl 2-methyl-7-methoxybenzo[*d*]imidazo[2,1-*b*]thiazole-3-carboxylate (3d). Obtained as a yellow-orange semi-solid (234 mg, 85%); ^{1}H NMR (300 MHz, $CDCl_3$) δ 8.80 (d, J = 9.3 Hz, 1H), 7.10 (s, 1H), 6.95 (d, J = 9.1 Hz, 1H), 3.94 (s, 3H), 3.83 (s, 3H), 2.59 (s, 3H); ^{13}C NMR (75 MHz, $CDCl_3$) δ 161.1, 152.0, 153.6, 150.9, 131.4, 128.3, 118.3, 118.1, 113.4, 107.6, 55.7, 51.4, 16.8. HRMS (ESI) calcd for $C_{13}H_{13}N_2O_3S$ [M+H]$^+$: 277.0641; found 277.0638.

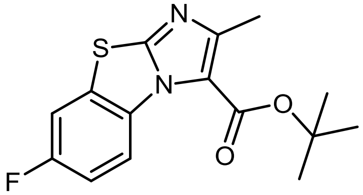

***tert*-Butyl 7-fluoro-2-methylbenzo[*d*]imidazo[2,1-*b*]thiazole-3-carboxylate (3e).** Obtained as a yellow solid (223 mg, 73%); mp 102-104 °C; ^{1}H NMR (300 MHz, $CDCl_3$) δ 9.01 (d, J = 9.1 Hz, 1H), 7.36 (d, J = 7.8 Hz, 1H), 7.16 (d, J = 8.4 Hz, 1H), 2.60 (s, 3H), 1.65 (s, 9H); ^{13}C NMR (75 MHz, $CDCl_3$) δ 161.2, 160.1, 157.9, 152.9, 150.5, 131.1, 131.0, 130.6, 119.9, 119.1, 119.0, 113.9, 113.5, 110.4, 110.0, 82.1, 28.4, 17.1. HRMS (ESI) calcd for $C_{15}H_{16}FN_2O_2S$ [M+H]$^+$: 307.0911; found 307.0914.

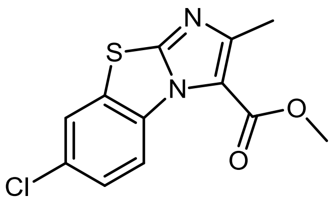

Methyl 7-chloro-2-methylbenzo[*d*]imidazo[2,1-*b*]thiazole-3-carboxylate (3f). Obtained as a yellow solid (220 mg, 78%); mp 139-141 °C; ^{1}H NMR (300 MHz, $CDCl_3$) δ 8.91 (d, J = 9.0 Hz, 1H), 7.62 (s, 1H), 7.39 (d, J = 9.0 Hz, 1H), 3.96 (s, 3H), 2.62 (s, 3H); ^{13}C NMR (75 MHz, $CDCl_3$) δ 161.0, 154.4, 151.4, 132.6, 131.2, 130.3, 126.6, 123.1, 120.7, 118.6, 51.7, 16.9. HRMS (ESI) calcd for $C_{12}H_{10}ClN_2O_2S$ [M+H]$^+$: 281.0146; found 281.0149.

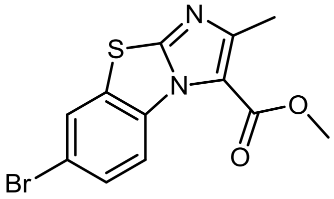

Methyl 7-bromo-2-methylbenzo[*d*]imidazo[2,1-*b*]thiazole-3-carboxylate (3g). Obtained as a yellow solid (270 mg, 83%); mp 170-172 °C; ^{1}H NMR (300 MHz, $CDCl_3$) δ 8.86 (d, J = 9.0 Hz, 1H), 7.78 (s, 1H), 7.53 (d, J = 9.0 Hz, 1H), 3.97 (s, 3H), 2.62 (s, 3H); ^{13}C NMR (75 MHz, $CDCl_3$) δ 161.1, 154.5, 151.3, 133.0, 131.5, 129.5, 126.0, 123.8, 118.9, 118.0, 51.7, 16.9. HRMS (ESI) calcd for $C_{12}H_{10}BrN_2O_2S$ [M+H]$^+$: 324.9641; found 324.9643.

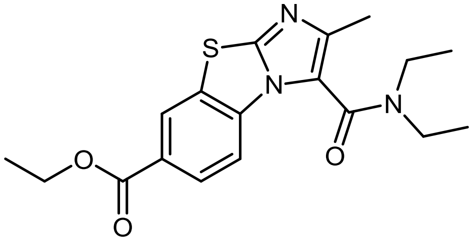

Ethyl 3-(diethylcarbamoyl)-2-methyl-benzo[*d*]imidazo[2,1-*b*] thiazole-7-carboxylate (3h). Obtained as a yellow semi-solid (281 mg, 78 %); ^{1}H NMR (300 MHz, $CDCl_3$) δ 8.36 (s, 1H), 8.06 (d, J = 8.7 Hz, 1H), 7.71 (d, J = 7.8 Hz, 1H), 4.38 (q, J = 7.2 Hz, 2H), 3.55 (broad, 4H), 1.38 (q, J = 7.2 Hz, 3H), 1.22 (broad, 6H); ^{13}C NMR (75 MHz, $CDCl_3$) δ 165.3, 161.8, 149.0, 144.1, 135.0, 130.0, 127.9, 127.0, 125.7, 113.6, 61.3, 42.9, 40.1, 14.5, 14.2, 14.0. HRMS (ESI) calcd for $C_{18}H_{22}N_3O_3S$ [M+H]$^+$: 360.1377; found 360.1375.

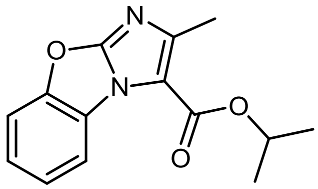

Isopropyl 2-methylbenzo[*d*]imidazo[2,1-*b*]oxazole-3-carboxy late (3j). Obtained as a pale yellow semi-solid (218 mg, 84%); ^{1}H NMR (300 MHz, $CDCl_3$) δ 8.28 (d, *J* = 9.3 Hz, 1H), 7.48 (d, *J* = 9.3 Hz, 1H), 7.35-7.25 (m, 2H), 5.37-5.22 (m, 1H), 2.60 (s, 3H), 1.42 (d, *J* = 6.3 Hz, 6H); ^{13}C NMR (75 MHz, $CDCl_3$) δ 160.0, 155.5, 150.6, 150.5, 127.3, 124.5, 124.3, 115.1, 113.8, 112.0, 68.4, 22.1, 16.2. HRMS (ESI) calcd for $C_{14}H_{15}N_2O_3$ $[M+H]^+$: 259.1077; found 259.1079.

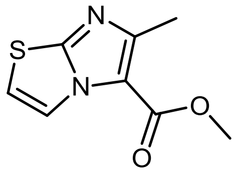

Methyl 6-methylimidazo[2,1-*b*]thiazole-5-carboxylate (3k). Obtained as a white solid (181 mg, 92%); mp 97-99 °C; ^{1}H NMR (300 MHz, $CDCl_3$) δ 8.02 (d, *J* = 4.5 Hz, 1H), 6.88 (d, *J* = 4.5 Hz, 1H), 3.90 (s, 3H), 2.59 (s, 3H); ^{13}C NMR (75 MHz, $CDCl_3$) δ 160.8, 153.2, 152.3, 121.1, 115.2, 112.6, 51.3, 15.9. HRMS (ESI) calcd for $C_8H_9N_2O_2S$ $[M+H]^+$: 197.0379; found 197.0379.

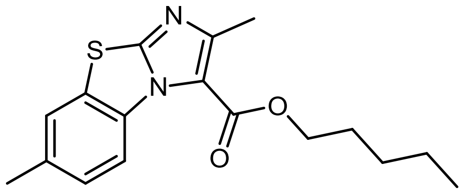

Pentyl 2,7-dimethyl-benzo[*d*]imidazo[2,1-*b*]thiazole-3-carboxy late (3l). Obtained as a yellow solid (262 mg, 83%); mp 59-62 °C; ^{1}H NMR (300 MHz, $CDCl_3$) δ 8.80 (d, *J* = 8.7 Hz, 1H), 7.41 (s, 1H), 7.20 (d, *J* = 9.0 Hz, 1H), 4.35 (t, *J* = 6.8 Hz, 2H), 2.62 (s, 3H), 2.42 (s, 3H), 1.83-1.75 (m, 2H), 1.47-1.33 (m, 4H), 0.93 (t, *J* = 6.9 Hz, 3H); ^{13}C NMR (75 MHz, $CDCl_3$) δ 160.8, 153.7, 151.3, 135.0, 132.0, 129.7, 127.2, 123.5, 118.4, 117.3, 64.8, 28.4, 22.8, 28.2, 22.3, 21.1, 17.0, 13.9. HRMS (ESI) calcd for $C_{17}H_{21}N_2O_2S$ $[M+H]^+$: 317.1319; found 317.1322.

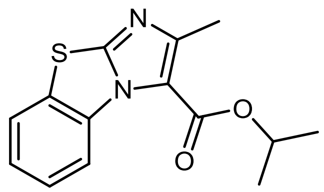

Isopropyl 2-methyl-benzo[*d*]imidazo[2,1-*b*]thiazole-3-carboxylate (3m). Obtained as a yellow oil (256 mg, 93%); ^{1}H NMR (300 MHz, $CDCl_3$) δ 8.95 (d, J = 8.4 Hz, 1H), 7.62 (d, J = 8.1 Hz, 1H), 7.41 (t, J = 8.0 Hz, 1H), 7.30 (t, J = 7.7 Hz, 1H), 5.36-5.22 (m, 1H), 2.62 (s, 3H), 1.42 (d, J = 6.3 Hz, 6H); ^{13}C NMR (75 MHz, $CDCl_3$) δ 160.2, 153.9, 151.3, 134.0, 129.6, 126.1, 124.8, 123.4, 118.8, 117.7, 68.5, 22.1, 17.0. HRMS (ESI) calcd for $C_{14}H_{15}N_2O_2S$ $[M+H]^+$: 275.0849; found 275.0849.

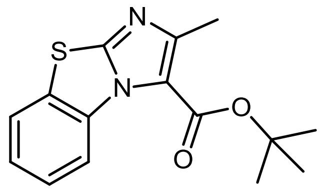

***tert*-Butyl 2-methylbenzo[*d*]imidazo[2,1-*b*]thiazole-3-carboxylate (3n).** Obtained as a yellow oil (259 mg, 90%); ^{1}H NMR (300 MHz, $CDCl_3$) δ 8.96 (d, J = 8.4 Hz, 1H), 7.64 (d, J = 8.1 Hz, 1H), 7.43 (t, J = 8.0 Hz, 1H), 7.32 (t, J = 7.7 Hz, 1H), 2.61 (s, 3H), 1.65 (s, 9H); ^{13}C NMR (75 MHz, $CDCl_3$) δ 160.1, 153.2, 150.9, 134.1, 129.7, 126.1, 124.8, 123.5, 119.8, 117.8, 81.9, 28.5, 17.1. HRMS (ESI) calcd for $C_{15}H_{17}N_2O_2S$ $[M+H]^+$: 289.1005; found 289.1007.

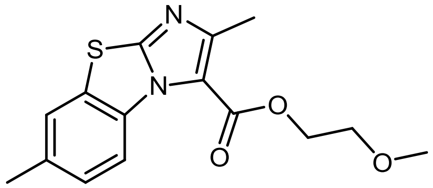

2-Methoxyethyl 2,7-dimethylbenzo[*d*]imidazo[2,1-*b*]thiazole-3-carboxylate (3o). Obtained as a yellow-orange solid (268 mg, 88%); mp 110-112 °C; ^{1}H NMR (300 MHz, $CDCl_3$) δ 8.76 (d, J = 8.7 Hz, 1H), 7.41 (s, 1H), 7.19 (d, J = 8.4 Hz, 1H), 4.50 (t, J = 4.7 Hz, 2H), 3.74 (t, J = 4.7 Hz, 2H), 3.43 (s, 3H), 2.64 (s, 3H), 2.42 (s, 3H); ^{13}C NMR (75 MHz, $CDCl_3$) δ 160.5, 154.4, 151.5, 135.0, 131.9, 129.7, 127.3, 123.5, 118.0, 117.2, 70.5, 63.5, 58.9, 21.1, 16.9. HRMS (ESI) calcd for $C_{15}H_{17}N_2O_3S$ $[M+H]^+$: 305.0954; found 305.0955.

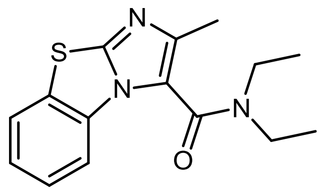

***N,N*-Diethyl 2-methylbenzo[*d*]imidazo[2,1-*b*]thiazole-3-carboxamide (3p).** Obtained as a yellow oil (256 mg, 89%); ^{1}H NMR (300 MHz, $CDCl_3$) δ 7.62 (t, J = 8.0 Hz, 2H), 7.33 (t, J = 7.8 Hz, 1H), 7.24 (t, J = 7.7 Hz, 1H), 3.52 (broad, 4H), 2.33 (s, 3H), 1.20 (broad, 6H); ^{13}C NMR (75 MHz, $CDCl_3$) δ 162.1, 148.1, 143.2, 132.2, 129.8, 126.1, 124.5, 123.9, 118.9, 113.9, 42.9, 39.7, 14.4, 13.8. HRMS (ESI) calcd for $C_{15}H_{18}N_3OS$ $[M+H]^+$: 288.1165; found 288.1168.

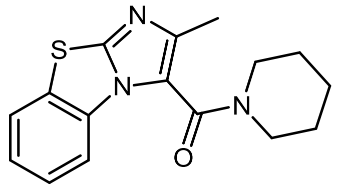

(2-Methylbenzo[*d*]imidazo[2,1-*b*]thiazol-3-yl)(piperidin-1-yl)methanone (3q). Obtained as a yellow-orange oil (263 mg, 88%); ^{1}H NMR (300 MHz, $CDCl_3$) δ 7.78 (d, J = 8.1 Hz, 1H), 7.64 (d, J = 8.1 Hz, 1H), 7.37 (t, J = 7.7 Hz, 1H), 7.28 (t, J = 7.7 Hz, 1H), 3.47 (broad, 4H), 2.36 (s, 3H), 1.67 (broad, 6H); ^{13}C NMR (75 MHz, $CDCl_3$) δ 161.3, 148.8, 144.2, 132.5, 129.9, 126.2, 124.6, 123.9, 118.7, 114.5, 47.4, 44.3, 26.4, 24.5, 14.7. HRMS (ESI) calcd for $C_{16}H_{18}N_3OS$ $[M+H]^+$: 300.1165; found 300.1167.

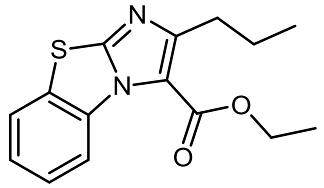

Ethyl 2-propylbenzo[*d*]imidazo[2,1-*b*]thiazole-3-carboxylate (3r). Obtained as a yellow solid (238 mg, 83%); mp 58-61 °C; ^{1}H NMR (300 MHz, $CDCl_3$) δ 8.90 (d, J = 8.4 Hz, 1H), 7.62 (d, J = 7.8 Hz, 1H), 7.40 (t, J = 7.8 Hz, 1H), 7.29 (t, J = 7.7 Hz, 1H), 4.42 (q, J = 7.2 Hz, 2H), 2.97 (t, J = 7.5 Hz, 2H), 1.83-1.69 (m, 2H), 1.43 (t, J = 7.2 Hz, 3H), 1.00 (t, J = 7.5 Hz, 3H); ^{13}C NMR (75 MHz, $CDCl_3$) δ 160.6, 158.3, 151.6, 134.0, 129.7, 126.1, 124.8, 123.5, 118.2, 117.6, 60.6, 32.3, 22.8, 14.3, 13.9. HRMS (ESI) calcd for $C_{15}H_{17}N_2O_2S$ $[M+H]^+$: 289.1005; found 289.1008.

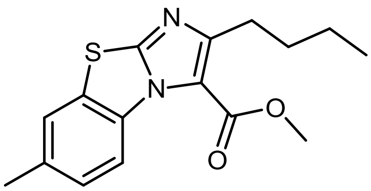

Methyl 2-butyl-7-methylbenzo[*d*]imidazo[2,1-*b*]thiazole-3-carboxylate (3s). Obtained as a yellow semi-solid (251 mg, 83%); ^{1}H NMR (300 MHz, $CDCl_3$) δ 8.67 (d, J = 8.7 Hz, 1H), 7.34 (s, 1H), 7.13 (d, J = 8.7 Hz, 1H), 3.91 (s, 3H), 2.94 (t, J = 7.7 Hz, 2H), 2.36 (s, 3H), 1.74-1.64 (m, 2H), 1.45-1.33 (m, 2H), 0.92 (t, J = 7.4 Hz, 3H); ^{13}C NMR (75 MHz, $CDCl_3$) δ 161.0, 158.3, 151.5, 134.8, 131.9, 129.7, 127.1, 123.3, 117.6, 117.0, 51.3, 31.5, 29.8, 22.5, 20.9, 13.8. HRMS (ESI) calcd for $C_{16}H_{19}N_2O_2S$ $[M+H]^+$: 303.1161; found 303.1164.

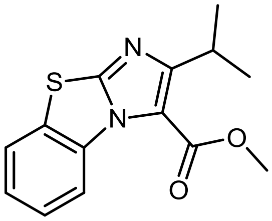

Methyl 2-isopropylbenzo[*d*]imidazo[2,1-*b*]thiazole-3-carbox ylate (3t). Obtained as a yellow semi-solid (190 mg, 69%); ^{1}H NMR (300 MHz, $CDCl_3$) δ 8.80 (d, J = 8.4 Hz, 1H), 7.59 (d, J = 7.5 Hz, 1H), 7.37 (t, J = 8.0 Hz, 1H), 7.26 (t, J = 7.7 Hz, 1H), 3.95 (s, 3H), 3.77-3.61 (m, 1H), 1.32 (d, J = 6.9 Hz, 6H); ^{13}C NMR (75 MHz, $CDCl_3$) δ 163.4, 161.1, 151.9, 133.9, 129.8, 126.0, 124.8, 123.4, 117.4, 116.7, 51.5, 28.0, 22.2. HRMS (ESI) calcd for $C_{14}H_{15}N_2O_2S$ $[M+H]^+$: 275.0849; found 275.0852.

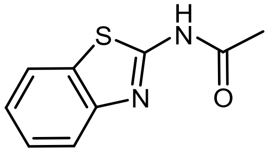

***N*-(benzo[*d*]thiazol-2-yl)acetamide (4)[a]** ^{1}H NMR (300 MHz, $CDCl_3$) δ 12.21 (broad, 1H), 7.84 (d, J = 7.8 Hz, 1H), 7.76 (d, J = 8.1 Hz, 1H), 7.45 (t, J = 7.2 Hz, 1H), 7.33 (t, J = 7.2 Hz, 1H), 2.28 (s, 3H); ^{13}C NMR (75 MHz, $CDCl_3$) δ 169.0, 160.2, 147.5, 131.8, 126.3, 124.0, 121.6, 120.2, 23.4. HRMS (ESI) calcd for $C_9H_9N_2OS$ $[M+H]^+$: 193.0430; found 193.0432.

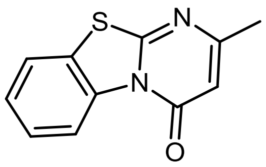

2-Methyl-4*H*-benzo[4,5]thiazolo[3,2-*a*]pyrimidin-4-one (5a)[b] Obtained as a light yellow solid (206 mg, 95%); mp 202-204 °C; ^{1}H NMR (300 MHz, $CDCl_3$) δ 8.94 (d, J = 7.5 Hz, 1H), 7.57 (d, J = 7.2 Hz, 1H), 7.44-7.33 (m, 2H), 6.16 (s, 1H), 2.30 (s, 3H); ^{13}C NMR (75 MHz, $CDCl_3$) δ 162.6, 161.1, 160.8, 135.8, 126.7, 126.6, 123.8, 121.5, 119.7, 106.9, 23.5. HRMS (ESI) calcd for $C_{11}H_9N_2OS$ $[M+H]^+$: 217.0430; found 217.0432.

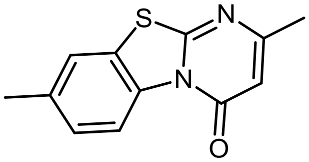

2,8-Dimethyl-4*H*-benzo[4,5]thiazolo[3,2-*a*]pyrimidin-4-one (5b). Obtained as a white solid (228 mg, 99%); mp 193-195 °C; ^{1}H NMR (300 MHz, $CDCl_3$) δ 8.79 (d, J = 8.7 Hz, 1H), 7.35 (s, 1H), 7.19 (d, J = 8.7 Hz, 1H), 6.15 (s, 1H), 2.39 (s, 3H), 2.31 (s, 3H); ^{13}C NMR (75 MHz, $CDCl_3$) δ 162.5, 161.2, 160.8, 137.1, 133.7, 127.7, 126.6, 123.8, 121.5, 119.4, 106.8, 23.5, 21.2. HRMS (ESI) calcd for $C_{12}H_{11}N_2OS$ $[M+H]^+$: 231.0586; found 231.0587.

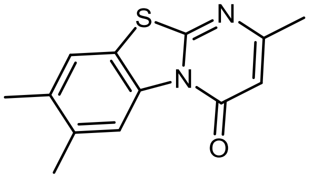

2,7,8-Trimethyl-4*H*-benzo[4,5]thiazolo[3,2-*a*]pyrimidin-4-one (5c). Obtained as a light yellow solid (237 mg, 97%); mp 178-180 °C; ^{1}H NMR (300 MHz, $CDCl_3$) δ 8.55 (s, 1H), 7.15 (s, 1H), 6.05 (s, 1H), 2.24 (s, 3H), 2.20 (s, 3H), 2.17 (s, 3H); ^{13}C NMR (75 MHz, $CDCl_3$) δ 162.1, 161.3, 160.5, 135.8, 135.6, 133.8, 121.4, 120.5, 119.9, 106.5, 23.4, 19.9, 19.7. HRMS (ESI) calcd for $C_{13}H_{13}N_2OS$ $[M+H]^+$: 245.0743; found 245.0744.

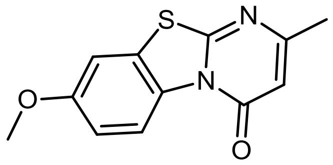

8-Methoxy-2-methyl-4*H*-benzo[4,5]thiazolo[3,2-*a*]pyrimidin-4-one (5d). Obtained as a yellow solid (222 mg, 90%); mp 175-177 °C; ^{1}H NMR (300 MHz, $CDCl_3$) δ 8.90 (d, J = 9.3 Hz, 1H), 7.09 (s, 1H), 6.99 (d, J = 9.3 Hz, 1H), 6.20 (s, 1H), 3.84 (s, 3H), 2.34 (s, 3H); ^{13}C NMR (75 MHz, $CDCl_3$) δ 162.4, 161.0, 160.7, 158.4, 129.8, 125.4, 120.8, 113.5, 106.9, 106.0, 55.7, 23.6. HRMS (ESI) calcd for $C_{12}H_{11}N_2O_2S$ $[M+H]^+$: 247.0535; found 247.0538.

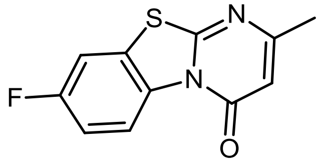

8-Fluoro-2-methyl-4*H*-benzo[4,5]thiazolo[3,2-*a*]pyrimidin-4-one (5e). Obtained as a white solid (222 mg, 95%); mp 200-202 °C; ^{1}H NMR (300 MHz, $CDCl_3$) δ 9.02-8.95 (m, 1H), 7.33 (d, J = 7.8 Hz, 1H), 7.16 (t, J = 8.9 Hz, 1H), 6.20 (s, 1H), 2.34 (s, 3H); ^{13}C NMR (75 MHz, $CDCl_3$) δ 162.8, 162.3, 160.9, 160.6, 159.0, 132.3, 125.7, 125.5, 121.3, 121.2, 114.5, 114.2, 108.9, 108.6, 107.2, 23.6. HRMS (ESI) calcd for $C_{11}H_8FN_2OS$ $[M+H]^+$: 235.0336; found 235.0336.

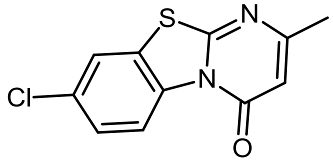

8-Chloro-2-methyl-4*H*-benzo[4,5]thiazolo[3,2-*a*]pyrimidin-4-one (5f). Obtained as a white solid (231 mg, 92%); mp 212-214 °C; ^{1}H NMR (300 MHz, $CDCl_3$) δ 8.96 (d, J = 9.0 Hz, 1H), 7.62 (s, 1H), 7.45 (d, J = 9.0 Hz, 1H), 6.24 (s, 1H), 2.37 (s, 3H); ^{13}C NMR (75 MHz, $CDCl_3$) δ 163.1, 160.8, 160.7, 134.5, 132.7, 127.3, 125.6, 121.5, 120.8, 107.3, 23.7. HRMS (ESI) calcd for $C_{11}H_8ClN_2OS$ $[M+H]^+$: 251.0040; found 251.0042.

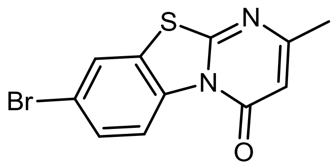

8-Bromo-2-methyl-4*H*-benzo[4,5]thiazolo[3,2-*a*]pyrimidin-4-one (5g). Obtained as a yellow solid (272 mg, 92%); mp 227-229 °C; ^{1}H NMR (300 MHz, $CDCl_3$) δ 8.89 (d, J = 9.0 Hz, 1H), 7.77 (s, 1H), 7.58 (d, J = 9.0 Hz, 1H), 6.23 (s, 1H), 2.36 (s, 3H); ^{13}C NMR (75 MHz, $CDCl_3$) δ 163.1, 160.7, 160.7, 134.9, 130.1, 125.9, 124.3, 121.0, 120.2, 107.3, 23.7. HRMS (ESI) calcd for $C_{11}H_8BrN_2OS$ $[M+H]^+$: 294.9535; found 294.9532.

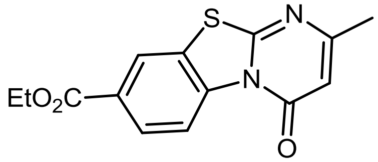

Ethyl 2-methyl-4-oxo-4*H*-benzo[4,5]thiazolo[3,2-*a*]pyrimidine-8-carboxylate (5h). Obtained as a white solid (246 mg, 85%); mp 196-198 °C; ^{1}H NMR (300 MHz, $CDCl_3$) δ 9.09 (d, J = 9.0 Hz, 1H), 8.35 (s, 1H), 8.16 (d, J = 9.0 Hz, 1H), 6.26 (s, 1H), 4.42 (q, J = 7.1 Hz, 2H), 2.38 (s, 3H), 1.42 (t, J = 7.1 Hz, 3H); ^{13}C NMR (75 MHz, $CDCl_3$) δ 165.0, 163.2, 161.5, 160.9, 129.2, 128.9, 128.3, 124.3, 123.2, 119.5, 107.3, 61.6, 23.7, 14.3. HRMS (ESI) calcd for $C_{14}H_{13}N_2O_3S$ $[M+H]^+$: 289.0642; found 289.0645.

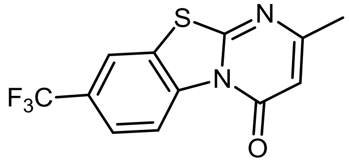

2-Methyl-8-(trifluoromethyl)-4*H*-benzo[4,5]thiazolo[3,2-*a*]py rimidin-4-one (5i). Obtained as a white solid (258 mg, 91%); mp 179-181 °C; ^{1}H NMR (300 MHz, $CDCl_3$) δ 9.08 (d, J = 8.7 Hz, 1H), 7.90 (s, 1H), 7.69 (d, J = 8.7 Hz, 1H), 6.20 (s, 1H), 2.34 (s, 3H); ^{13}C NMR (75 MHz, $CDCl_3$) δ 163.2, 161.0, 160.6, 138.1, 129.8, 129.4, 128.9, 128.7, 128.5, 125.1, 124.9, 124.0, 123.9, 123.9, 123.8, 121.5, 119.9, 119.1, 119.0, 119.0, 118.9, 117.9, 107.3, 23.6. HRMS (ESI) calcd for $C_{12}H_8F_3N_2OS$ $[M+H]^+$: 285.0304; found 285.0306.

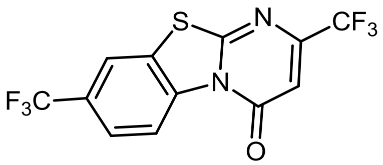

2,8-Bis(trifluoromethyl)-4*H*-benzo[4,5]thiazolo[3,2-*a*]pyrimidin-4-one (5j). Obtained as a yellow solid (321 mg, 95%); mp 178-180 °C; ^{1}H NMR (300 MHz, CDCl$_3$) δ 9.18 (d, J = 8.7 Hz, 1H), 8.04 (s, 1H), 7.83 (d, J = 8.7 Hz, 1H), 6.79 (s, 1H); ^{13}C NMR (75 MHz, CDCl$_3$) δ 163.6, 159.9, 151.7, 151.2, 150.7, 150.2, 137.5, 131.0, 130.5, 130.1, 129.6, 128.6, 125.7, 125.3, 125.0, 124.7, 124.6, 124.6, 122.1, 121.4, 120.4, 119.5, 119.5, 119.4, 119.4, 118.4, 117.8, 114.8, 107.2, 107.2, 107.2, 107.1, 107.1. HRMS (ESI) calcd for $C_{12}H_5F_6N_2OS$ [M+H]$^+$: 339.0021; found 339.0023.

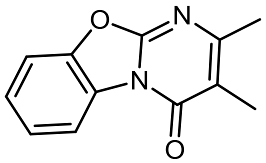

2,3-Dimethyl-*4*H-benzo[4,5]oxazolo[3,2-*a*]pyrimidin-4-one (5k). Obtained as a white solid (170 mg, 79%); mp 155-157 °C; ^{1}H NMR (300 MHz, CDCl$_3$) δ 8.37 (d, J = 6.9 Hz, 1H), 7.50-7.36 (m, 3H), 2.40 (s, 3H), 2.14 (s, 3H); ^{13}C NMR 75 MHz, CDCl$_3$) δ 159.8, 159.4, 152.5, 144.6, 126.9, 126.3, 125.0, 116.3, 113.6, 110.7, 22.4, 11.0. HRMS (ESI) calcd for $C_{12}H_{11}N_2O_2$ [M+H]$^+$: 215.0815; found 215.0818.

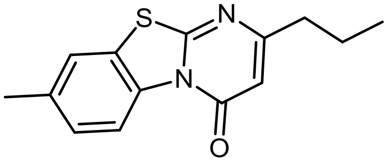

8-Methyl-2-propyl-4*H*-benzo[4,5]thiazolo[3,2-*a*]pyrimidin-4-one (5l). Obtained as a light yellow solid (226 mg, 88%); mp 95-97 °C; ^{1}H NMR (300 MHz, CDCl$_3$) δ 8.71 (d, J = 8.4 Hz, 1H), 7.27 (s, 1H), 7.11 (d, J = 8.4 Hz, 1H), 6.09 (s, 1H), 2.46 (t, J = 7.7 Hz, 2H), 2.31 (s, 3H), 1.72-1.58 (m, 2H), 0.92 (t, J = 7.4 Hz, 3H); ^{13}C NMR (75 MHz, CDCl$_3$) δ 165.9, 161.0, 160.8, 136.9, 133.5, 122.4, 123.8, 121.3, 119.1, 106.1, 39.0, 21.1, 21.1, 13.5. HRMS (ESI) calcd for $C_{14}H_{15}N_2OS$ [M+H]$^+$: 259.0900; found 259.0903.

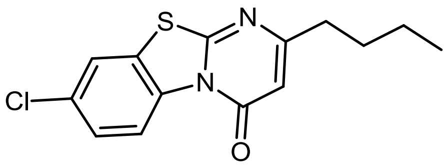

2-Butyl-8-chloro-4*H*-benzo[4,5]thiazolo[3,2-*a*]pyrimidin-4-one (5m). Obtained as a light yellow solid (252 mg, 86%); mp 113-115 °C; ^{1}H NMR (300 MHz, $CDCl_3$) δ 8.62 (d, J = 9.0 Hz, 1H), 7.36 (s, 1H), 7.14 (d, J = 8.7 Hz, 1H), 5.99 (s, 1H), 2.40 (t, J = 7.7 Hz, 2H), 1.58-1.46 (m, 2H), 1.32-1.18 (m, 2H), 0.80 (t, J = 7.2 Hz, 3H); ^{13}C NMR (75 MHz, $CDCl_3$) δ 166.3, 160.2, 160.2, 133.9, 132.0, 126.5, 125.2, 120.9, 120.0, 106.1, 36.6, 29.7, 21.9, 13.5. HRMS (ESI) calcd for $C_{14}H_{14}ClN_2OS$ $[M+H]^+$: 293.0510; found 293.0513.

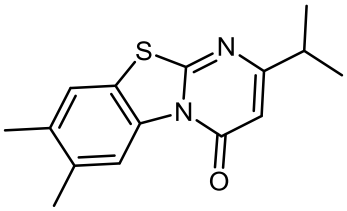

2-Isopropyl-7,8-dimethyl-4*H*-benzo[4,5]thiazolo[3,2-*a*]pyrimidin-4-one (5n). Obtained as a yellow solid (210 mg, 77%); mp 145-147 °C; ^{1}H NMR (300 MHz, $CDCl_3$) δ 8.72 (s, 1H), 7.28 (s, 1H), 6..20 (s, 1H), 2.86-2.72 (m, 1H), 2.31 (s, 3H), 2.27 (s, 3H), 1.25 (d, J = 6.6 Hz, 6H); ^{13}C NMR (75 MHz, $CDCl_3$) δ 171.2, 161.5, 161.4, 136.0, 135.7, 134.0, 121.6, 120.9, 120.2, 104.1, 36.3, 21.1, 20.0, 19.8. HRMS (ESI) calcd for $C_{15}H_{17}N_2OS$ $[M+H]^+$: 273.1056; found 273.1018.

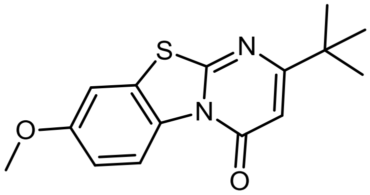

2-(*tert*-Butyl)-8-methoxy-4*H*-benzo[4,5]thiazolo[3,2-*a*]pyrimidin-4-one (5o). Obtained as a yellow solid (202 mg, 70%); mp 196-198 °C; ^{1}H NMR (300 MHz, $CDCl_3$) δ 8.93 (d, J = 9.3 Hz, 1H), 7.12 (s, 1H), 7.00 (d, J = 9.3 Hz, 1H), 6.35 (s, 1H), 3.86 (s, 3H), 1.30 (s, 9H); ^{13}C NMR (75 MHz, $CDCl_3$) δ 173.4, 161.7, 160.3, 158.3, 129.8, 125.7, 120.7, 113.3, 106.1, 103.4, 55.7, 37.2, 28.8. HRMS (ESI) calcd for $C_{15}H_{17}N_2O_2S$ $[M+H]^+$: 289.1005; found 289.1008.

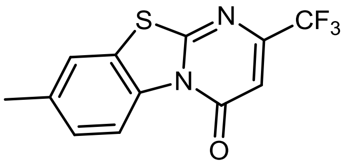

8-Methyl-2-(trifluoromethyl)-4*H*-benzo[4,5]thiazolo[3,2-*a*]pyrimidin-4-one (5p). Obtained as a yellow solid (281 mg, 99%); mp 192-194 °C; ^{1}H NMR (300 MHz, $CDCl_3$) δ 8.80 (d, J = 8.4 Hz, 1H), 7.46 (s, 1H), 7.28 (d, J = 8.7 Hz, 1H), 6.66 (s, 1H), 2.46 (s, 3H); ^{13}C NMR (75 MHz, $CDCl_3$) δ 163.5, 159.8, 151.0, 150.6, 150.1, 149.6, 138.4, 133.0, 128.3, 125.9, 124.3, 122.1, 121.8, 119.6, 118.6, 114.9, 106.4, 106.3, 106.3, 106.2, 21.3. HRMS (ESI) calcd for $C_{12}H_8F_3N_2OS$ $[M+H]^+$: 285.0305; found 285.0305.

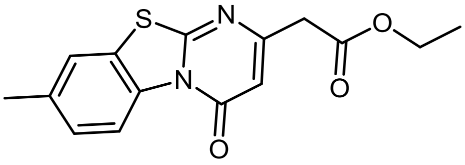

Ethyl 2-(8-methyl-4-oxo-4*H*-benzo[4,5]thiazolo[3,2-*a*]pyrimi din-2-yl)acetate (5q). Obtained as a light yellow solid (278 mg, 92%); mp 119-121 °C; ^{1}H NMR (300 MHz, $CDCl_3$) δ 8.67 (d, J = 8.7 Hz, 1H), 7.28 (s, 1H), 7.11 (d, J = 8.7 Hz, 1H), 6.23 (s, 1H), 4.12 (q, J = 7.2 Hz, 2H), 3.53 (s, 2H), 2.31 (s, 3H), 1.20 (t, J = 7.2 Hz, 3H),; ^{13}C NMR (75 MHz, $CDCl_3$) δ 168.8, 161.4, 160.3, 157.8, 137.2, 133.2, 127.5, 123.8, 121.4, 119.1, 108.0, 61.0, 42.5, 21.0, 13.9. HRMS (ESI) calcd for $C_{15}H_{15}N_2O_3S$ $[M+H]^+$: 303.0798; found 303.0799.

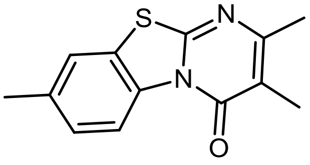

2,3,8-Trimethyl-4*H*-benzo[4,5]thiazolo[3,2-*a*]pyrimidin-4-one (5r). Obtained as a light yellow solid (230 mg, 94%); mp 156-158 °C; ^{1}H NMR (300 MHz, $CDCl_3$) δ 8.79 (d, J = 8.4 Hz, 1H), 7.30 (s, 1H), 7.16 (d, J = 8.7 Hz, 1H), 2.36 (s, 3H), 2.30 (s, 3H), 2.09 (s, 3H); ^{13}C NMR (75 MHz, $CDCl_3$) δ 161.3, 157.8, 157.2, 136.8, 133.7, 127.4, 124.1, 121.5, 119.1, 114.3, 21.8, 21.2, 11.1. HRMS (ESI) calcd for $C_{13}H_{13}N_2OS$ $[M+H]^+$: 245.0743; found 245.0745.

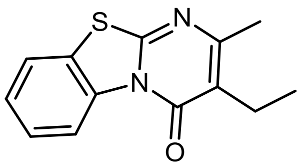

3-Ethyl-2-methyl-4*H*-benzo[4,5]thiazolo[3,2-*a*]pyrimidin-4-one (5s). Obtained as a light yellow solid (220 mg, 90%); mp 130-132 °C; ^{1}H NMR (300 MHz, $CDCl_3$) δ 8.21 (d, J = 7.8 Hz, 1H), 7.30 (d, J = 7.8 Hz, 1H), 7.18-7.06 (m, 2H), 2.40 (q, J = 7.4 Hz, 2H), 2.14 (s, 3H), 0.96 (t, J = 7.4 Hz, 3H); ^{13}C NMR (75 MHz, $CDCl_3$) δ 160.5, 157.1, 156.8, 135.5, 126.0, 125.9, 123.7, 121.0, 119.8, 119.0, 20.8, 18.7, 12.3. HRMS (ESI) calcd for $C_{13}H_{13}N_2OS$ $[M+H]^+$: 245.0743; found 245.0746.

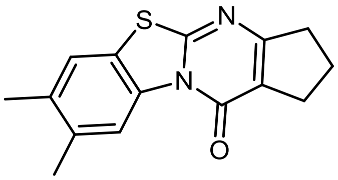

7,8-Dimethyl-2,3-dihydrobenzo[4,5]thiazolo[3,2-*a*]cyclopenta [*d*]pyrimidin-11(1*H*)-one (5t). Obtained as a yellow solid (238 mg, 88%); mp 167-169 °C; ^{1}H NMR (300 MHz, $CDCl_3$) δ 8.68 (s, 1H), 7.20 (s, 1H), 2.87-2.79 (m, 4H), 2.25 (s, 3H), 2.21 (s, 3H), 2.15-2.01 (m, 2H); ^{13}C NMR (75 MHz, $CDCl_3$) δ 167.4, 161.4, 159.1, 135.7, 135.5, 134.0, 121.5, 121.0, 120.1, 118.6, 34.5, 27.0, 21.4, 20.0, 19.7. HRMS (ESI) calcd for $C_{15}H_{15}N_2OS$ $[M+H]^+$: 271.0900; found 271.0898.

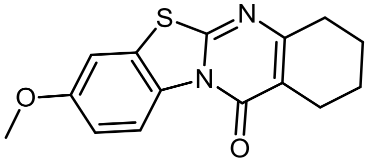

8-Methoxy-3,4-dihydro-1*H*-benzo[4,5]thiazolo[2,3-*b*]quinazo lin-12(2*H*)-one (5u). Obtained as a yellow solid (249 mg, 87%); mp 181-183 °C; ^{1}H NMR (300 MHz, $CDCl_3$) δ 8.81 (d, J = 9.0 Hz, 1H), 7.00 (s, 1H), 6.89 (d, J = 9.3 Hz, 1H), 3.78 (s, 3H), 2.65-2.52 (m, 4H), 1.81-1.70 (m, 4H); ^{13}C NMR (75 MHz, $CDCl_3$) δ 160.9, 158.8, 158.0, 157.2, 129.7, 125.4, 120.0, 115.9, 113.0, 106.0, 55.6, 31.6, 22.1, 22.1, 21.8. HRMS (ESI) calcd for $C_{15}H_{15}N_2O_2S$ $[M+H]^+$: 287.0849; found 287.0851.

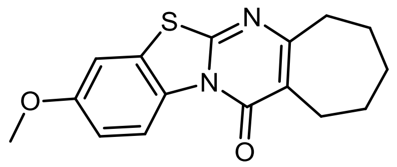

3-Methoxy-8,9,10,11-tetrahydrobenzo[4,5]thiazolo[3,2-*a*]cyclohepta[*d*]pyrimidin-12(7*H*)-one (5v). Obtained as a light yellow solid (265 mg, 88%); mp 174-176 °C; ^{1}H NMR (300 MHz, $CDCl_3$) δ 8.93 (d, J = 9.3 Hz, 1H), 7.06 (s, 1H), 6.96 (d, J = 9.0 Hz, 1H), 3.83 (s, 3H), 2.87-2.80 (m, 4H), 1.89-1.79 (m, 2H), 1.71-1.55 (m, 4H); ^{13}C NMR (75 MHz, $CDCl_3$) δ 164.9, 161.1, 158.2, 157.2, 130.1, 125.8, 120.7, 120.5, 113.2, 106.0, 55.6, 38.2, 32.3, 26.5, 25.4, 24.0. HRMS (ESI) calcd for $C_{16}H_{17}N_2O_2S$ $[M+H]^+$: 301.1005; found 301.1008.

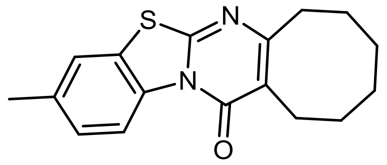

3-Methyl-9,10,11,12-tetrahydro-7*H*-benzo[4,5]thiazolo[3,2-*a*]cycloocta[*d*]pyrimidin-13(8*H*)-one (5w). Obtained as a light yellow solid (251 mg, 84%); mp 172-174 °C; ^{1}H NMR (300 MHz, $CDCl_3$) δ 8.90 (d, J = 8.7 Hz, 1H), 7.40 (s, 1H), 7.25 (d, J = 8.7 Hz, 1H), 2.83-2.74 (m, 4H), 2.43 (s, 3H), 1.83-1.69 (m, 4H), 1.50-1.39 (m, 4H); ^{13}C NMR (75MHz, $CDCl_3$) δ 162.4, 161.0, 158.1, 137.0, 134.1, 127.6, 124.3, 121.7, 119.4, 118.5, 34.4, 29.6, 29.2, 26.4, 26.1, 23.9, 21.3. HRMS (ESI) calcd for $C_{17}H_{19}N_2OS$ $[M+H]^+$: 299.1213; found 299.1216.

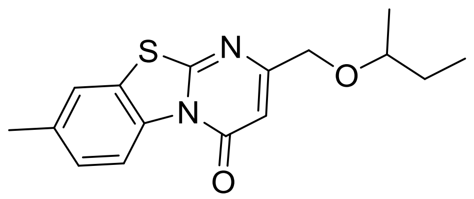

2-(*sec*-Butoxymethyl)-8-methyl-4*H*-benzo[4,5]thiazolo[3,2-*a*]pyrimidin-4-one (5x). Obtained as a yellow semisolid (197 mg, 65%); ^{1}H NMR (300 MHz, $CDCl_3$) δ 7.66 (s, 1H), 7.47-7.38 (m, 2H), 7.20 (d, J = 8.4 Hz, 1H), 5.00-4.85 (m, 1H), 3.77 (s, 2H), 2.44 (s, 3H), 1.68-1.51 (m, 2H), 1.24 (d, J = 6.3 Hz, 3H), 0.90 (t, J = 7.5 Hz, 3H); ^{13}C NMR (75 MHz, $CDCl_3$) δ 170.5, 146.0, 140.6, 134.8, 130.2, 130.1, 127.0, 124.3, 112.2, 109.7, 73.0, 35.4, 28.7, 21.3, 19.4, 9.6. HRMS (ESI) calcd for $C_{16}H_{19}N_2O_2S$ $[M+H]^+$: 303.1162; found 303.1165.

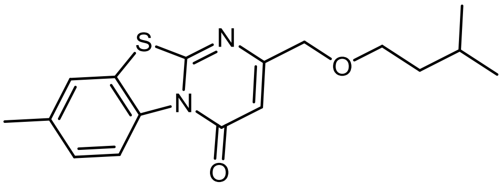

2-((Isopentyloxy)methyl)-8-methyl-4*H*-benzo[4,5]thiazolo[3,2-*a*]pyrimidin-4-one (5y). Obtained as a yellow semisolid (220 mg, 69%); ^{1}H NMR (300 MHz, $CDCl_3$) δ 7.63 (s, 1H), 7.43 (s, 1H), 7.38 (d, *J* = 8.1 Hz, 1H), 7.18 (d, *J* = 8.1 Hz, 1H), 4.17 (t, *J* = 6.9 Hz, 2H), 3.77 (s, 2H), 2.42 (s, 3H), 1.76-1.60 (m, 1H), 1.54 (q, *J* = 6.8 Hz, 2H), 0.90 (d, *J* = 6.6 Hz, 6H); ^{13}C NMR (75 MHz, $CDCl_3$) δ 170.9, 146.9, 140.4, 134.7, 130.1, 130.1, 127.0, 124.2, 112.1, 109.7, 63.7, 37.2, 35.1, 25.0, 22.4, 21.2. HRMS (ESI) calcd for $C_{17}H_{21}N_2O_2S$ $[M+H]^+$: 317.1318; found 317.1319.

References

[a] Rothweiler, U.; Stensen, W.; Brandsdal, B. O.; Isaksson, J.; Leeson, F. A.; Engh, R. A.; Svendsen, J. S. M. *J. Med. Chem.* **2016**, *59*, 9814–9824.

[b] Landreau, C.; Deniaud, D.; Evian, M.; Reliquet, A.; Meslin, J. C. *J. Chem.Soc.*, *Perkin Trans. I* **2002**, 741–745.

NMR Spectra

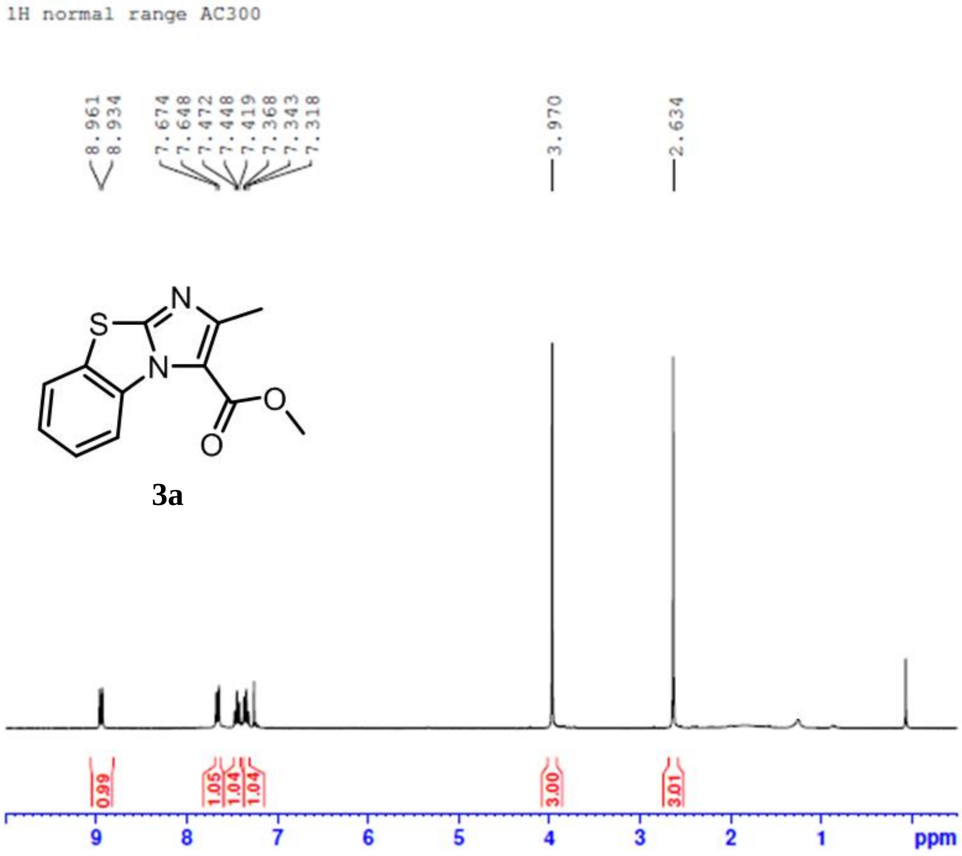

1H normal range AC300
8.961
8.934
7.674
7.648
7.472
7.448
7.419
7.368
7.343
7.318
3.970
2.634
3a
0.99
1.05
1.04
1.04
3.00
3.01
9
8
7
6
5
4
3
2
1
ppm

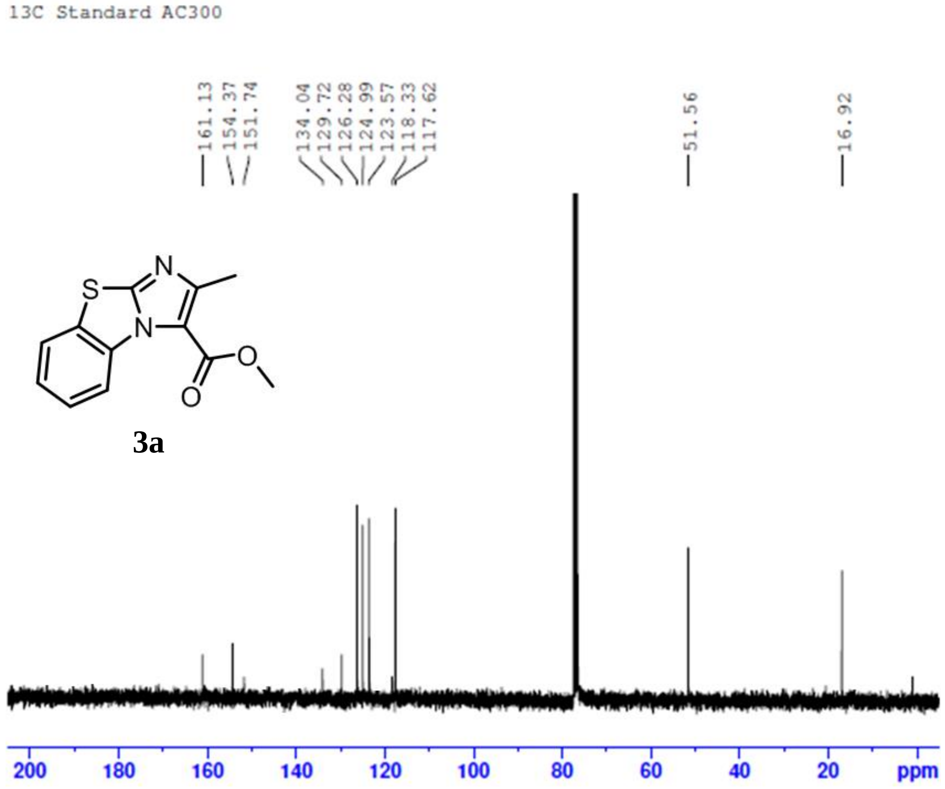

13C Standard AC300
161.13
154.37
151.74
134.04
129.72
126.28
124.99
123.57
118.33
117.62
51.56
16.92
3a
200
180
160
140
120
100
80
60
40
20
ppm

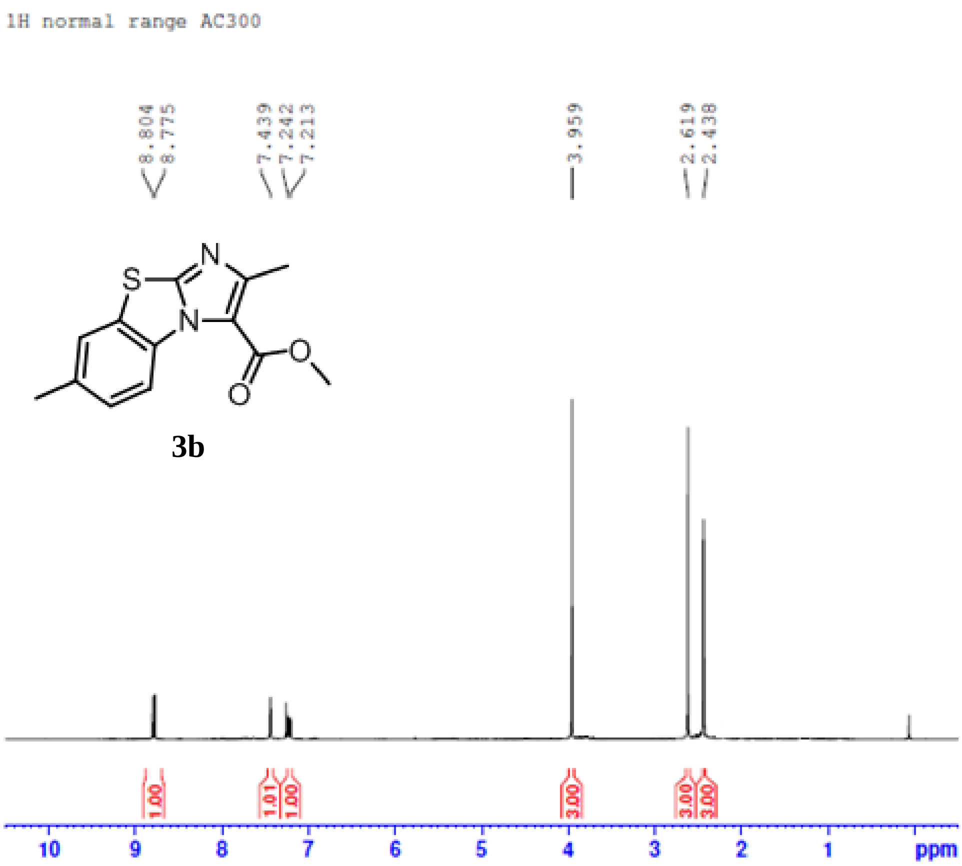
1H normal range AC300
8.804
8.775
7.439
7.242
7.213
3.959
2.619
2.438
S
N
N
O
O
3b
1.00
1.01
1.00
3.00
3.00
3.00
10
9
8
7
6
5
4
3
2
1
ppm

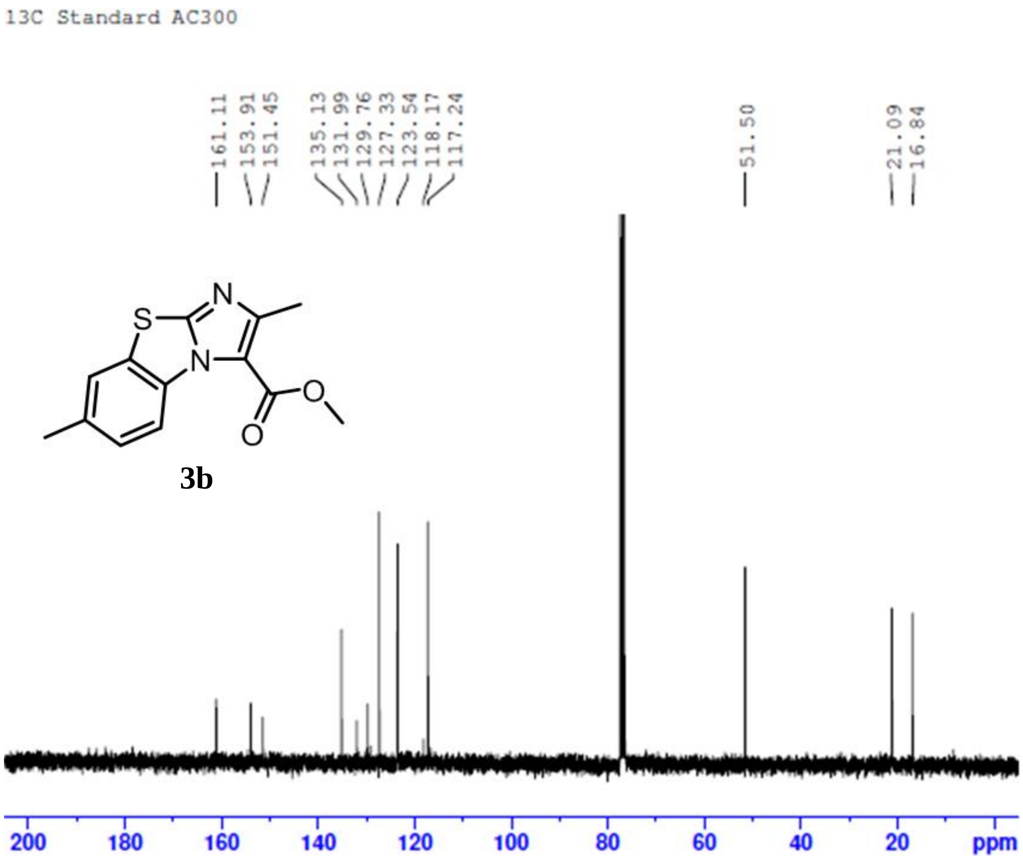
13C Standard AC300
161.11
153.91
151.45
135.13
131.99
129.76
127.33
123.54
118.17
117.24
51.50
21.09
16.84
S
N
N
O
O
3b
200
180
160
140
120
100
80
60
40
20
ppm

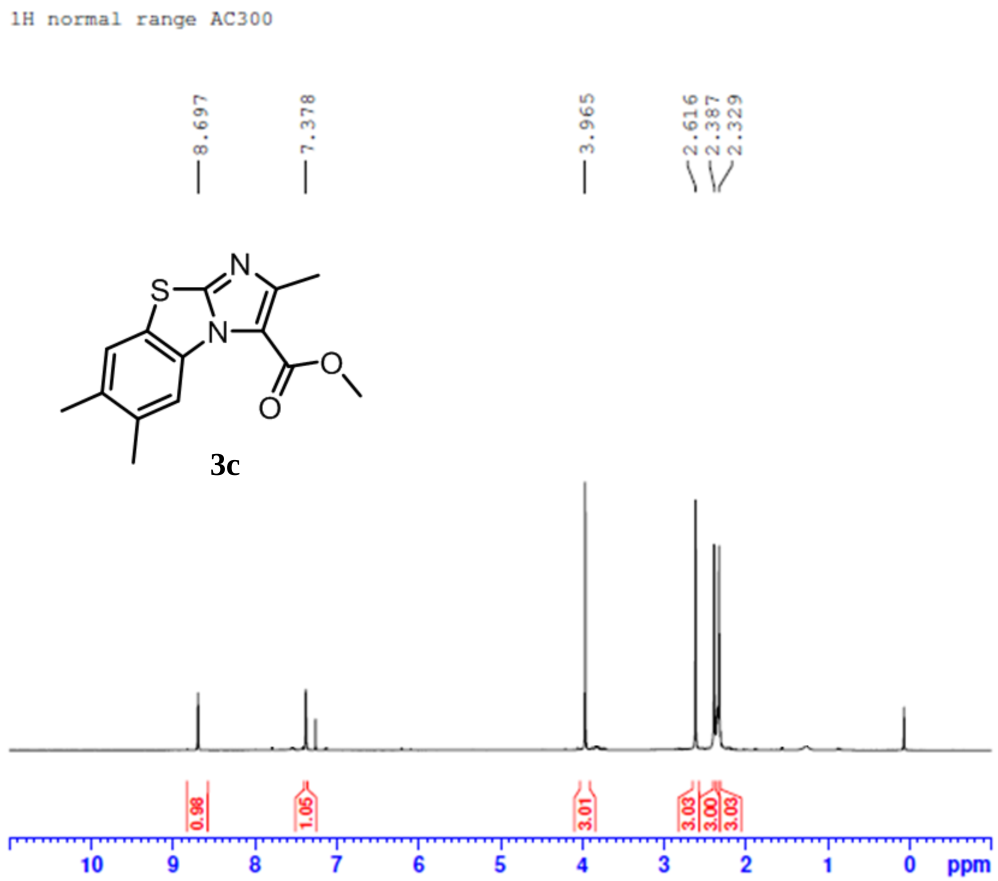
1H normal range AC300
8.697
7.378
3.965
2.616
2.387
2.329
3c
0.98
1.05
3.01
3.03
3.00
3.03
10 9 8 7 6 5 4 3 2 1 0 ppm

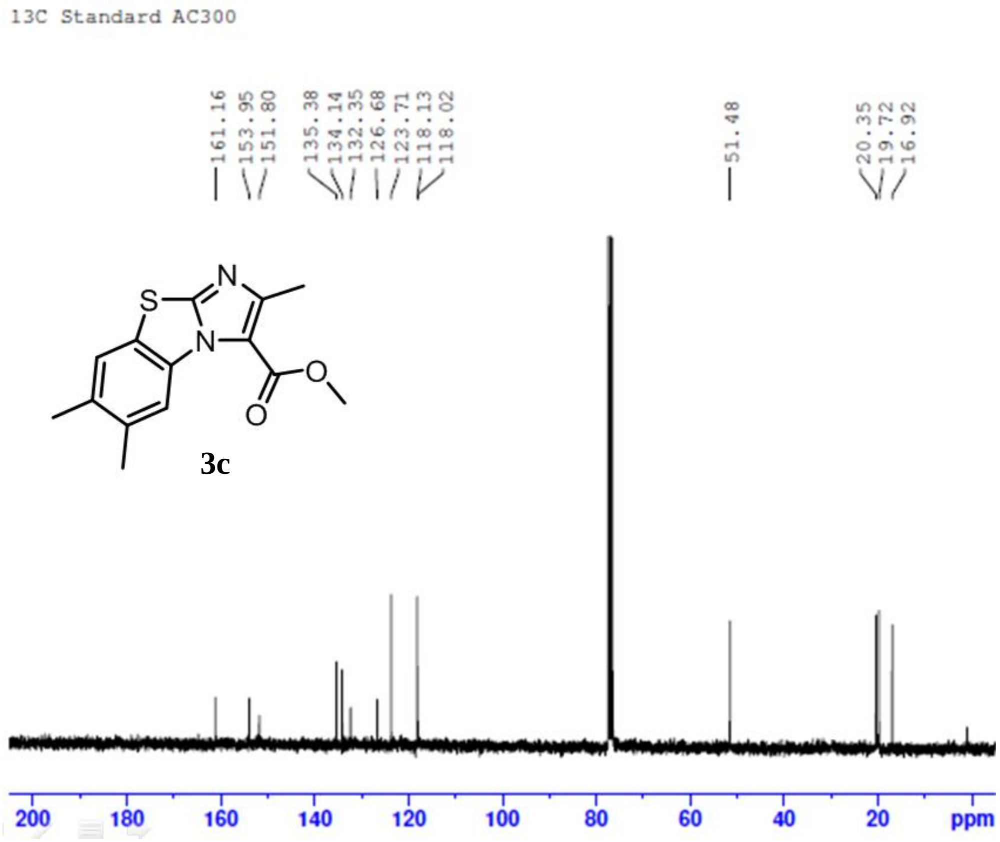
13C Standard AC300
161.16
153.95
151.80
135.38
134.14
132.35
126.68
123.71
118.13
118.02
51.48
20.35
19.72
16.92
3c
200 180 160 140 120 100 80 60 40 20 ppm

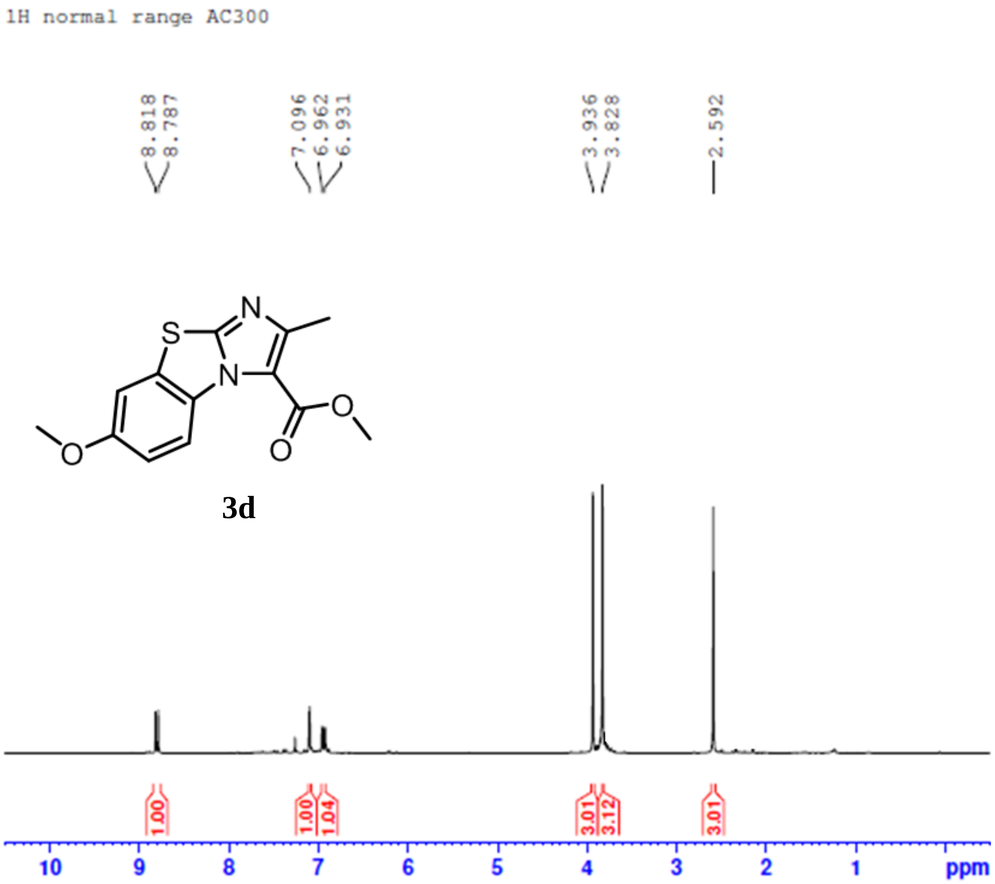
1H normal range AC300
8.818
8.787
7.096
6.962
6.931
3.936
3.828
2.592
3d
1.00
1.00
1.04
3.01
3.12
3.01
10 9 8 7 6 5 4 3 2 1 ppm

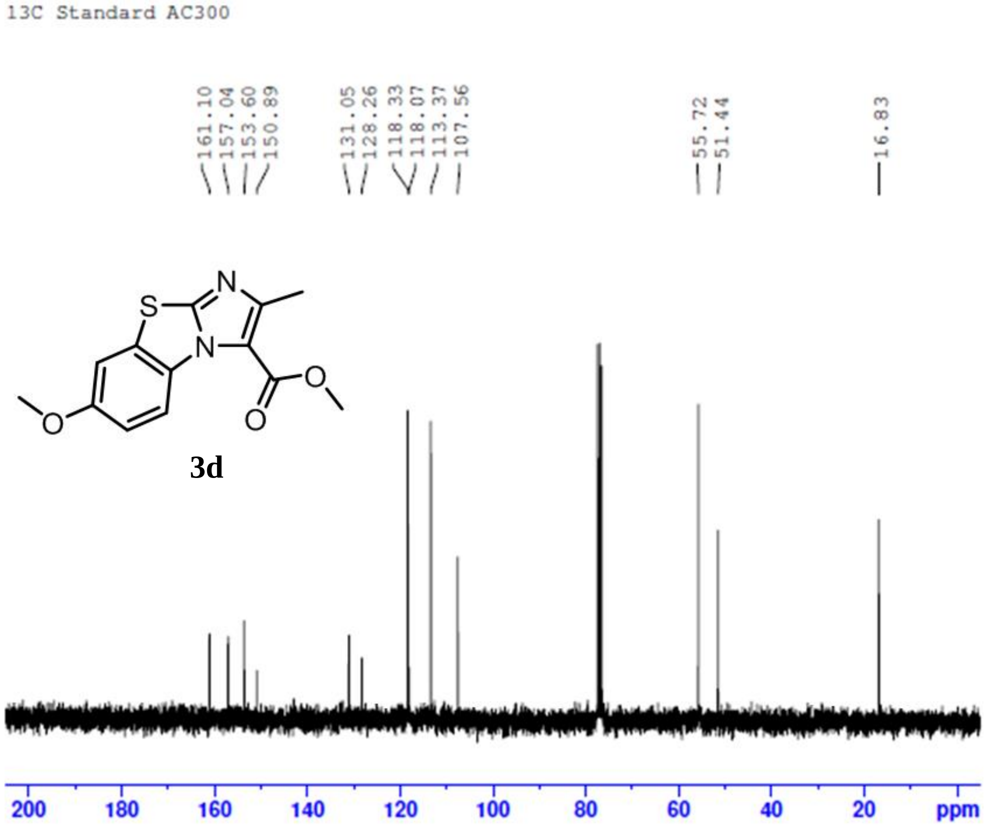
13C Standard AC300
161.10
157.04
153.60
150.89
131.05
128.26
118.33
118.07
113.37
107.56
55.72
51.44
16.83
3d
200 180 160 140 120 100 80 60 40 20 ppm

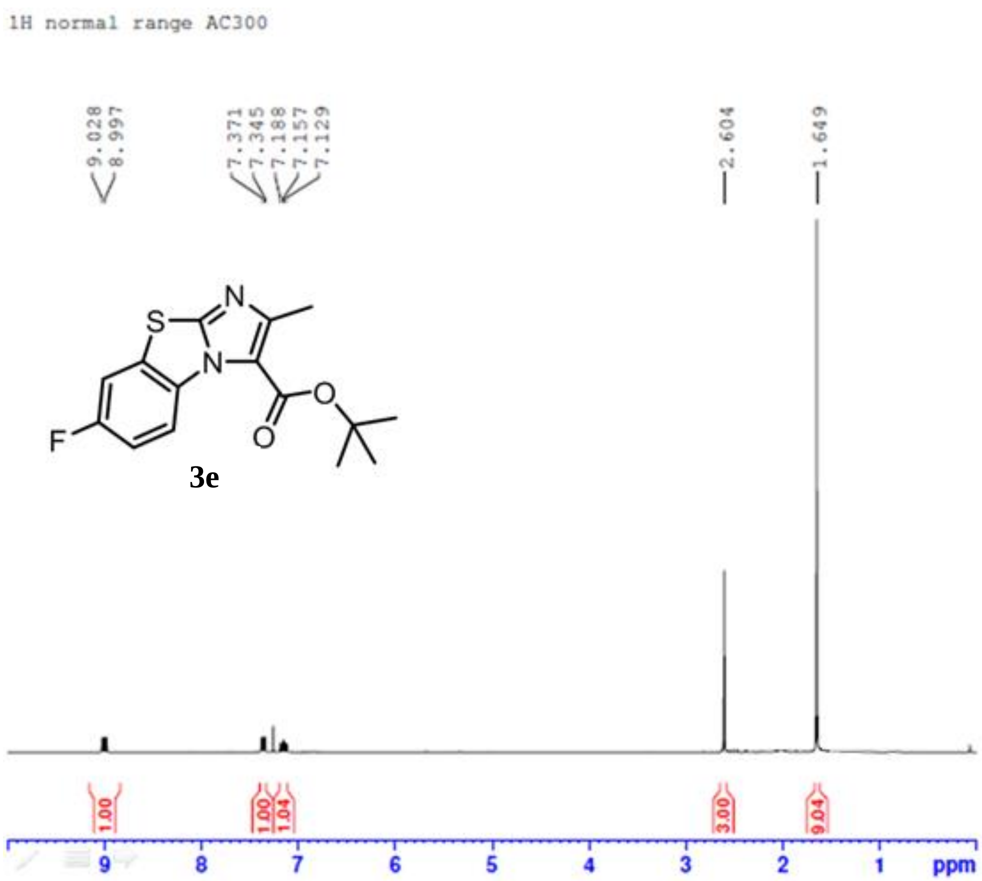
1H normal range AC300
9.028
8.997
7.371
7.345
7.188
7.157
7.129
2.604
1.649
3e
1.00
1.00
1.04
3.00
9.04
9
8
7
6
5
4
3
2
1
ppm

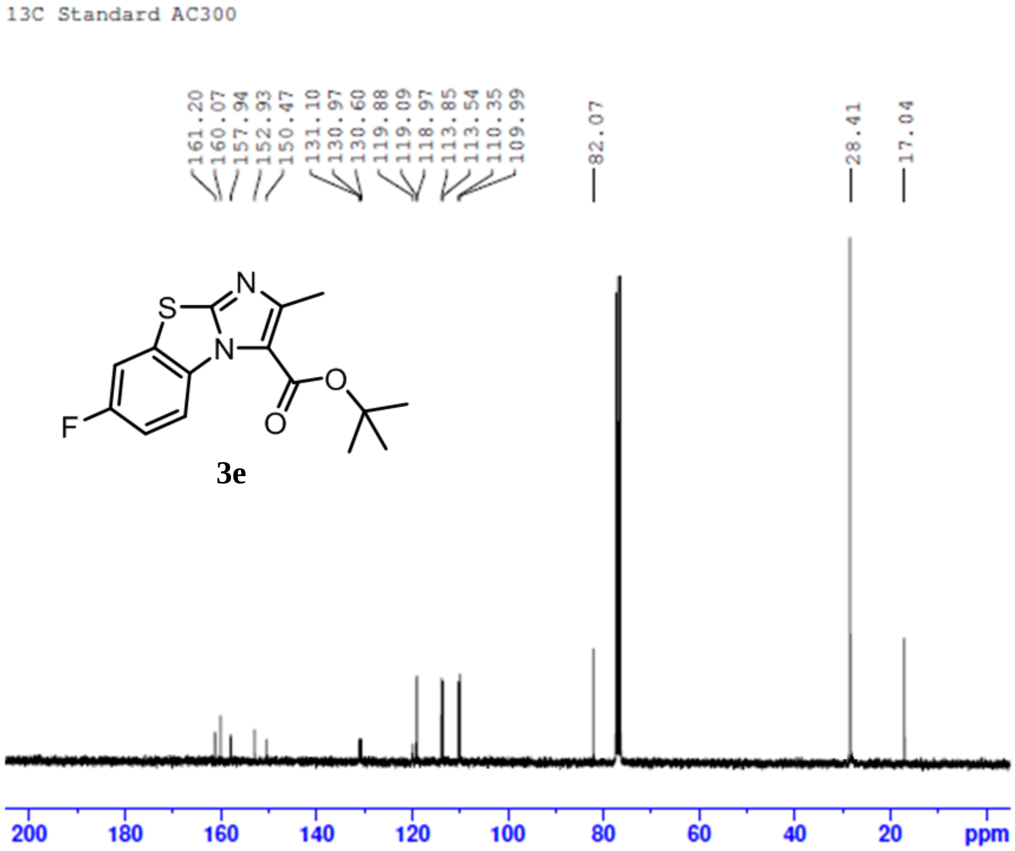
13C Standard AC300
161.20
160.07
157.94
152.93
150.47
131.10
130.97
130.60
119.88
119.09
118.97
113.85
113.54
110.35
109.99
82.07
28.41
17.04
3e
200
180
160
140
120
100
80
60
40
20
ppm

1H normal range AC300

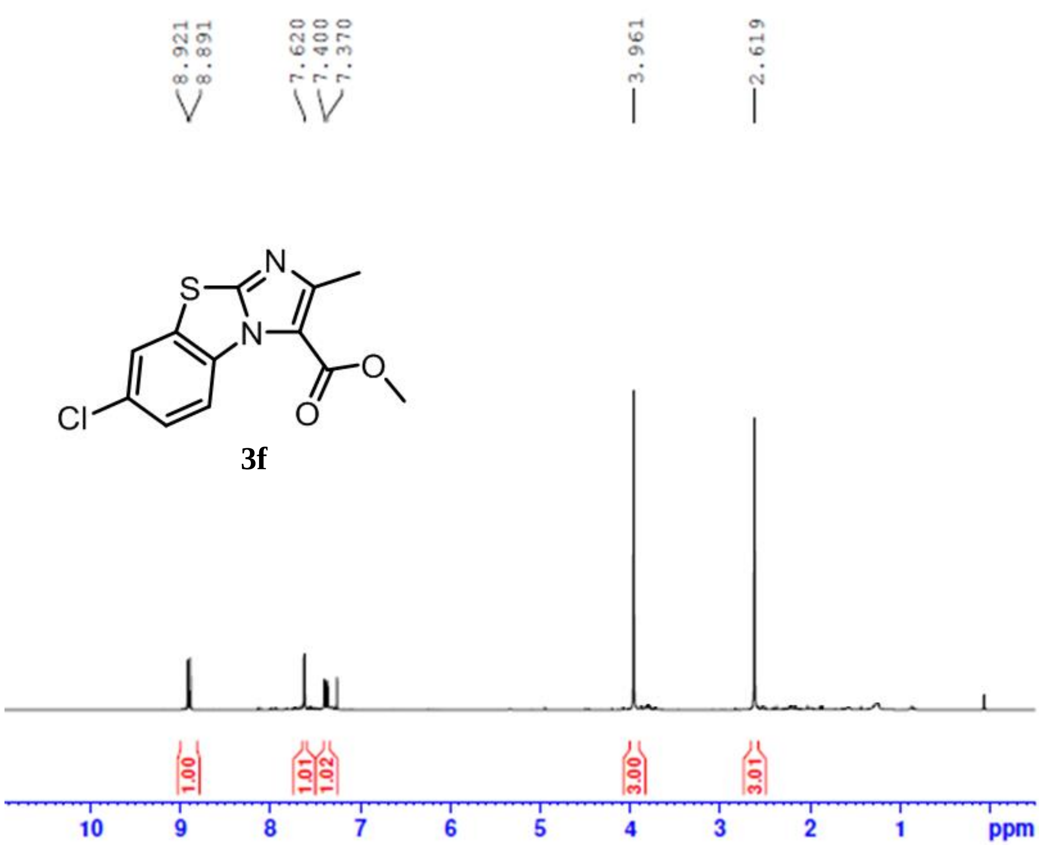

13C Standard AC300

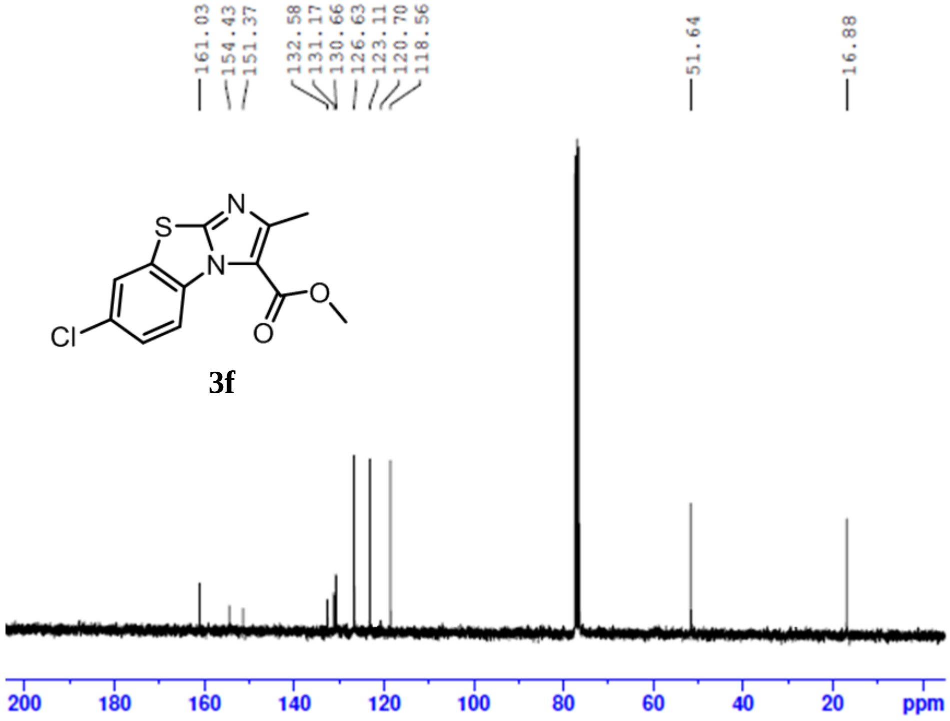

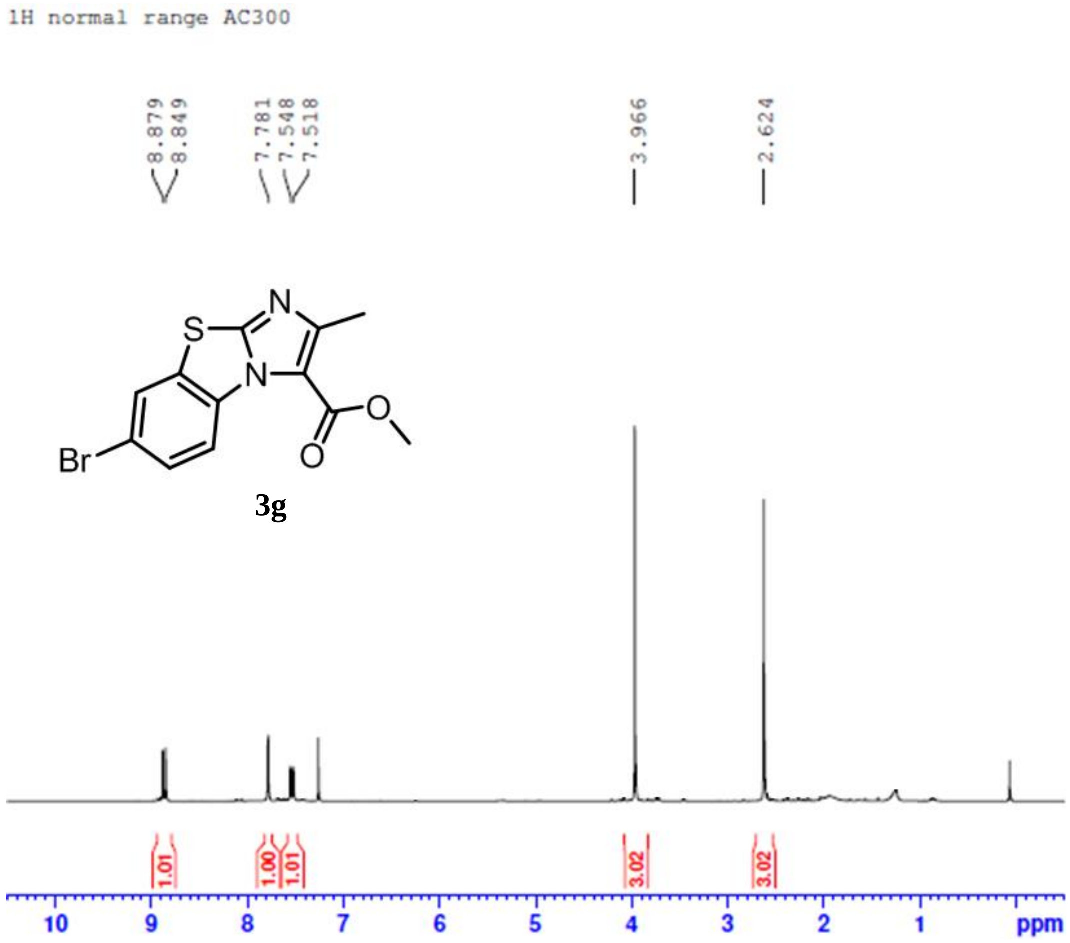
1H normal range AC300
8.879
8.849
7.781
7.548
7.518
3.966
2.624
S
N
N
O
O
Br
3g
1.01
1.00
1.01
3.02
3.02
10
9
8
7
6
5
4
3
2
1
ppm

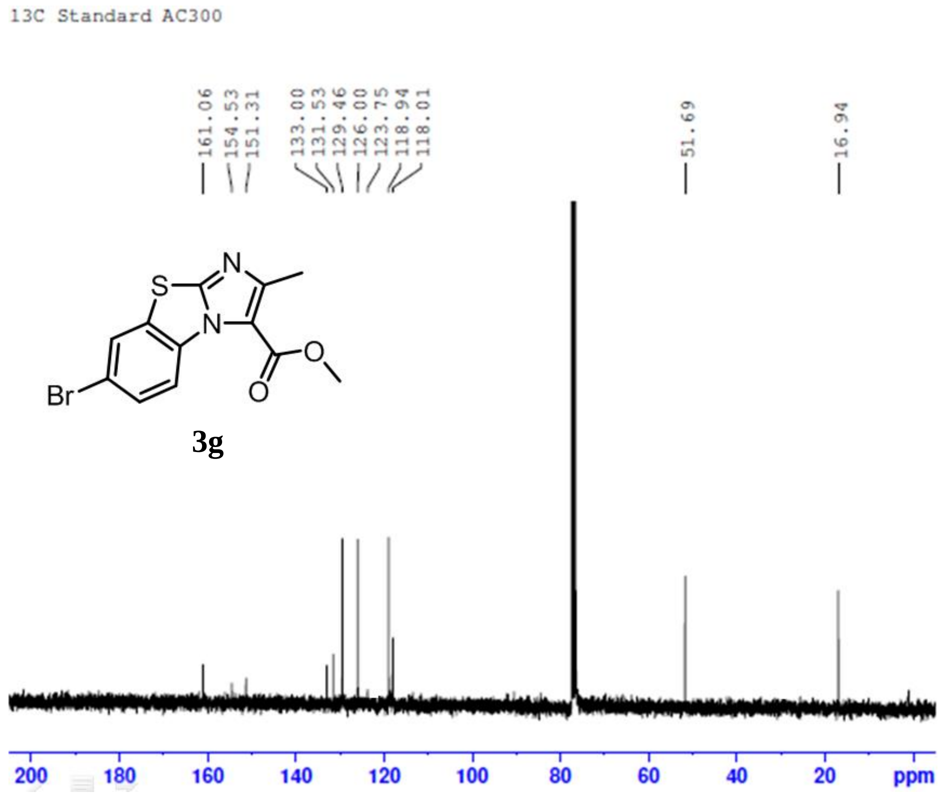
13C Standard AC300
161.06
154.53
151.31
133.00
131.53
129.46
126.00
123.75
118.94
118.01
51.69
16.94
S
N
N
O
O
Br
3g
200
180
160
140
120
100
80
60
40
20
ppm

1H normal range AC300

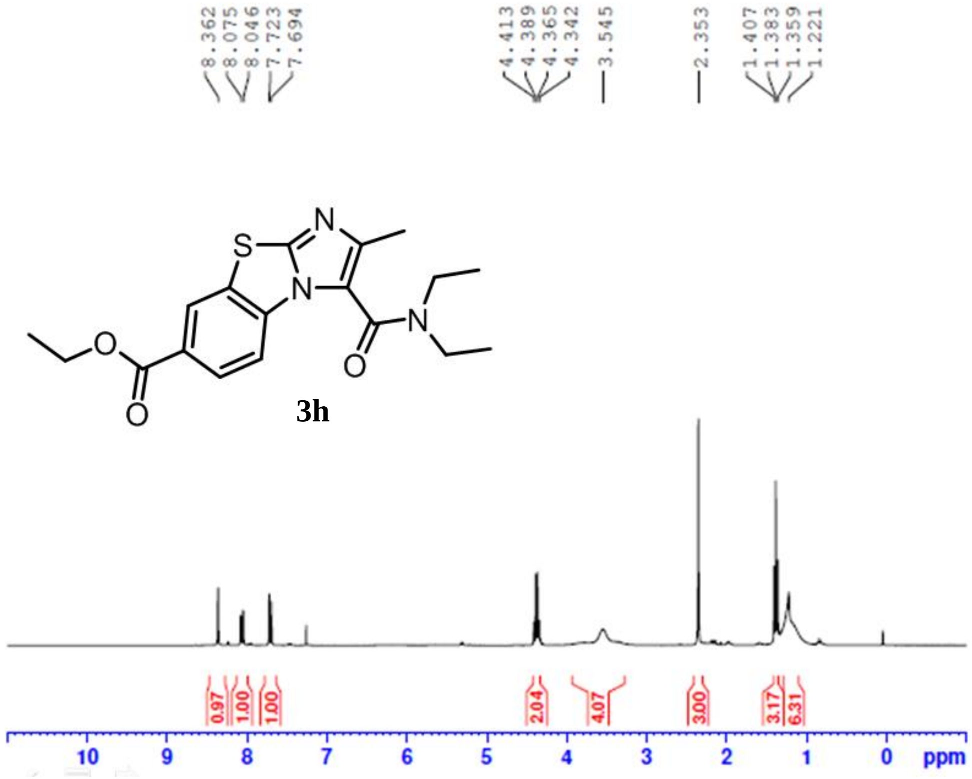

13C Standard AC300

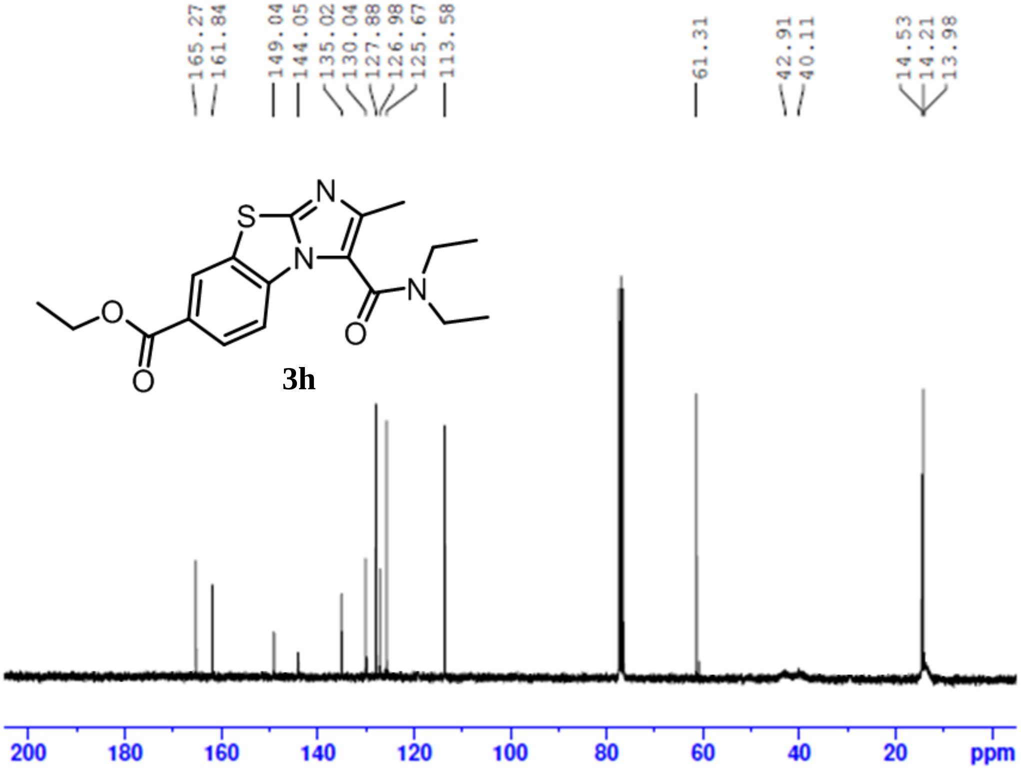

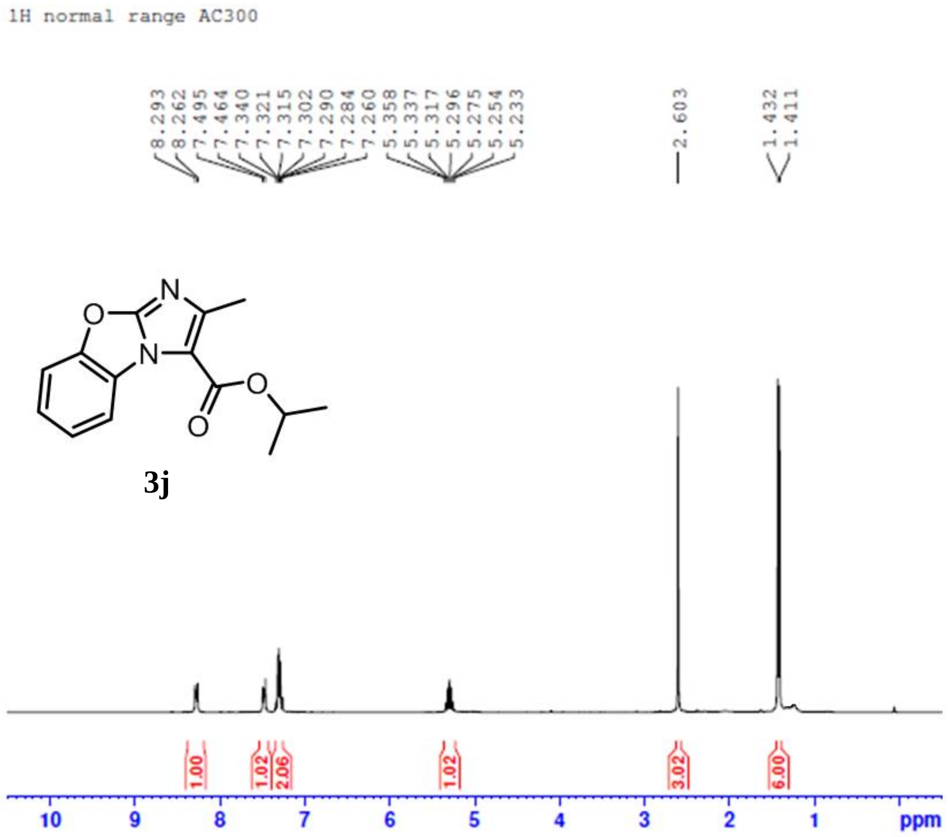
1H normal range AC300
8.293
8.262
7.495
7.464
7.340
7.321
7.315
7.302
7.290
7.284
7.260
5.358
5.337
5.317
5.296
5.275
5.254
5.233
2.603
1.432
1.411
3j
1.00
1.02
2.06
1.02
3.02
6.00
10 9 8 7 6 5 4 3 2 1 ppm

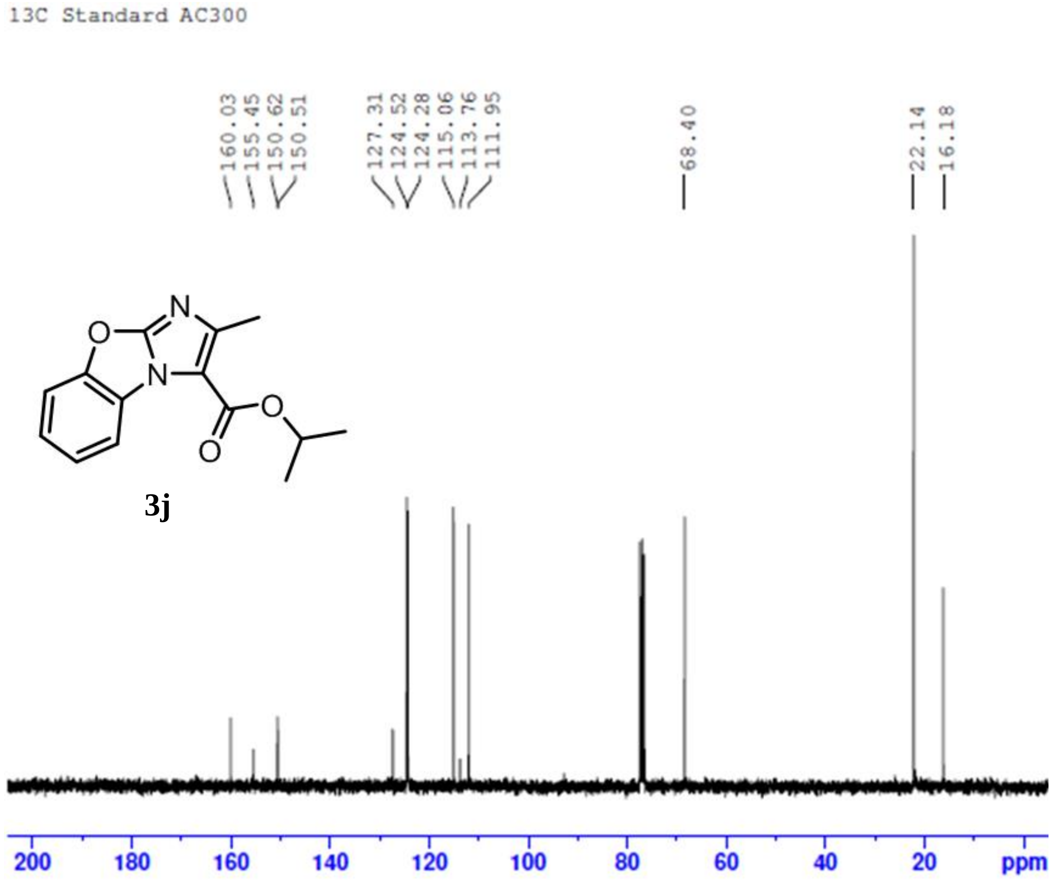
13C Standard AC300
160.03
155.45
150.62
150.51
127.31
124.52
124.28
115.06
113.76
111.95
68.40
22.14
16.18
3j
200 180 160 140 120 100 80 60 40 20 ppm

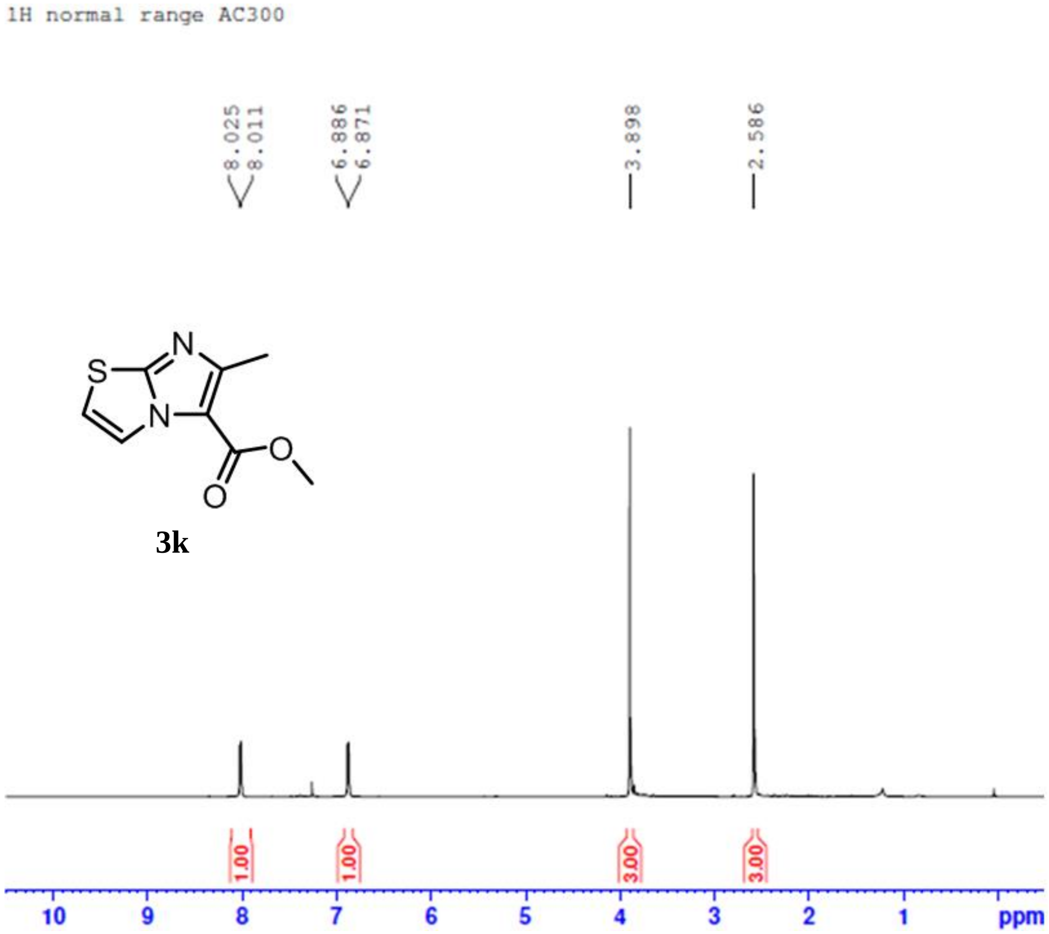
1H normal range AC300
8.025
8.011
6.886
6.871
3.898
2.586
S
N
N
O
O
3k
1.00
1.00
3.00
3.00
10
9
8
7
6
5
4
3
2
1
ppm

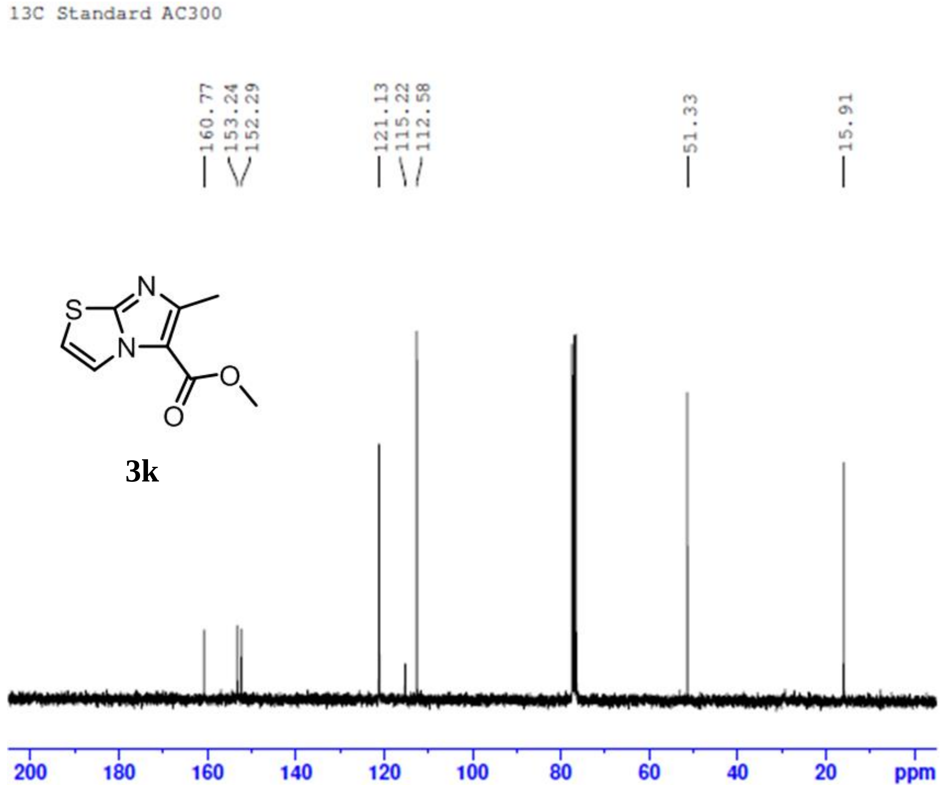
13C Standard AC300
160.77
153.24
152.29
121.13
115.22
112.58
51.33
15.91
S
N
N
O
O
3k
200
180
160
140
120
100
80
60
40
20
ppm

1H normal range AC300

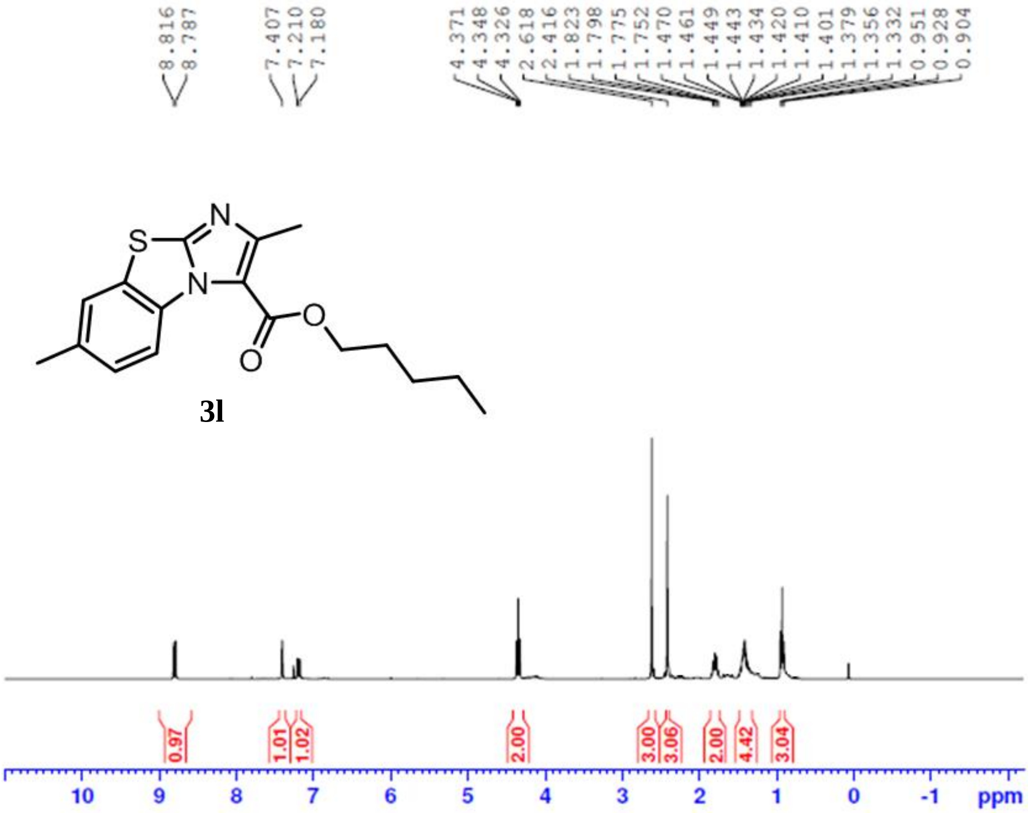

3l

13C Standard AC300

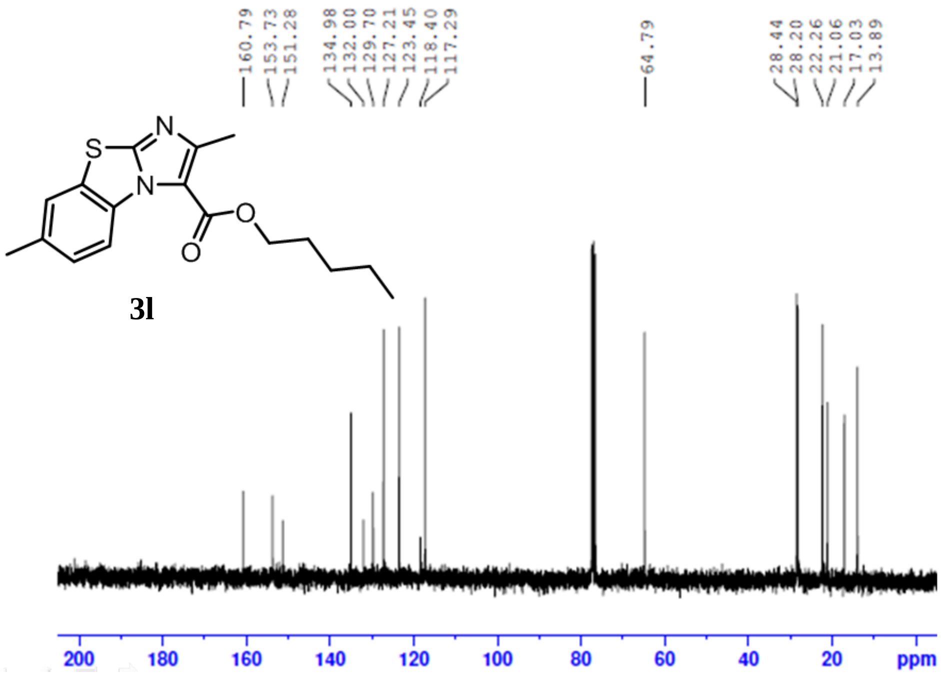

3l

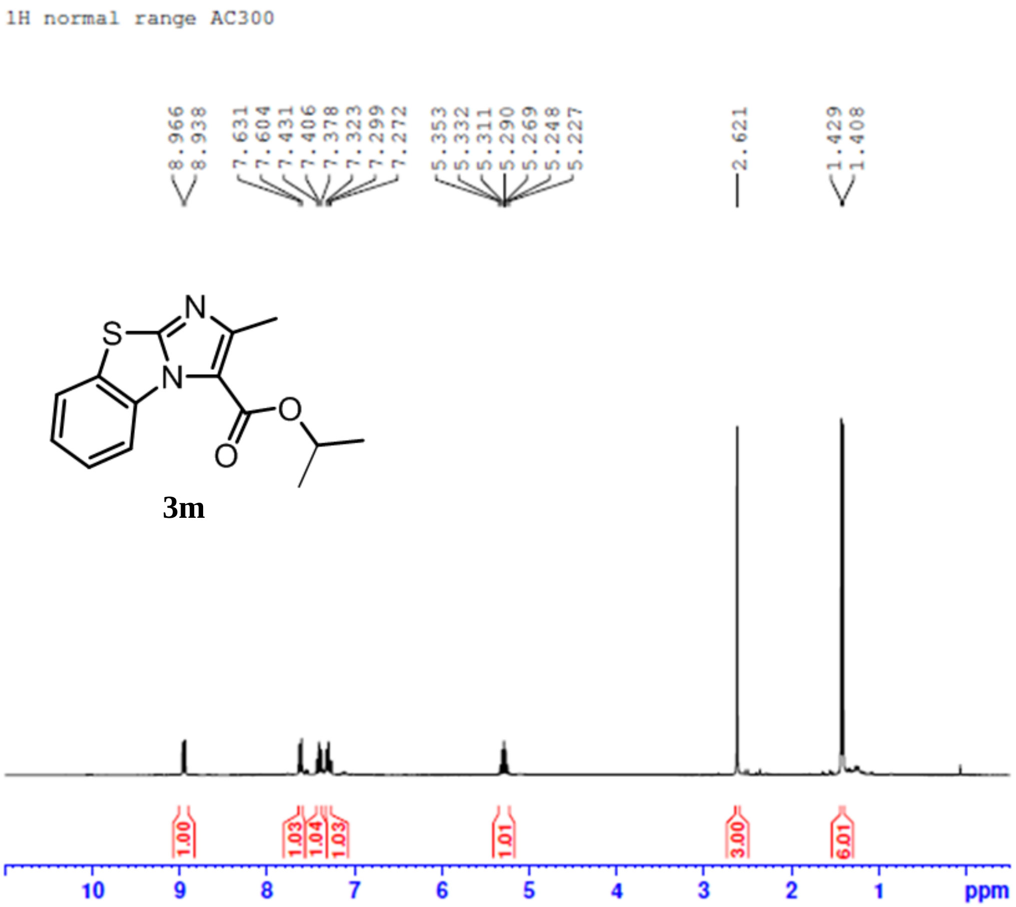
1H normal range AC300
8.966
8.938
7.631
7.604
7.431
7.406
7.378
7.323
7.299
7.272
5.353
5.332
5.311
5.290
5.269
5.248
5.227
2.621
1.429
1.408
3m
1.00
1.03
1.04
1.03
1.01
3.00
6.01
10 9 8 7 6 5 4 3 2 1 ppm

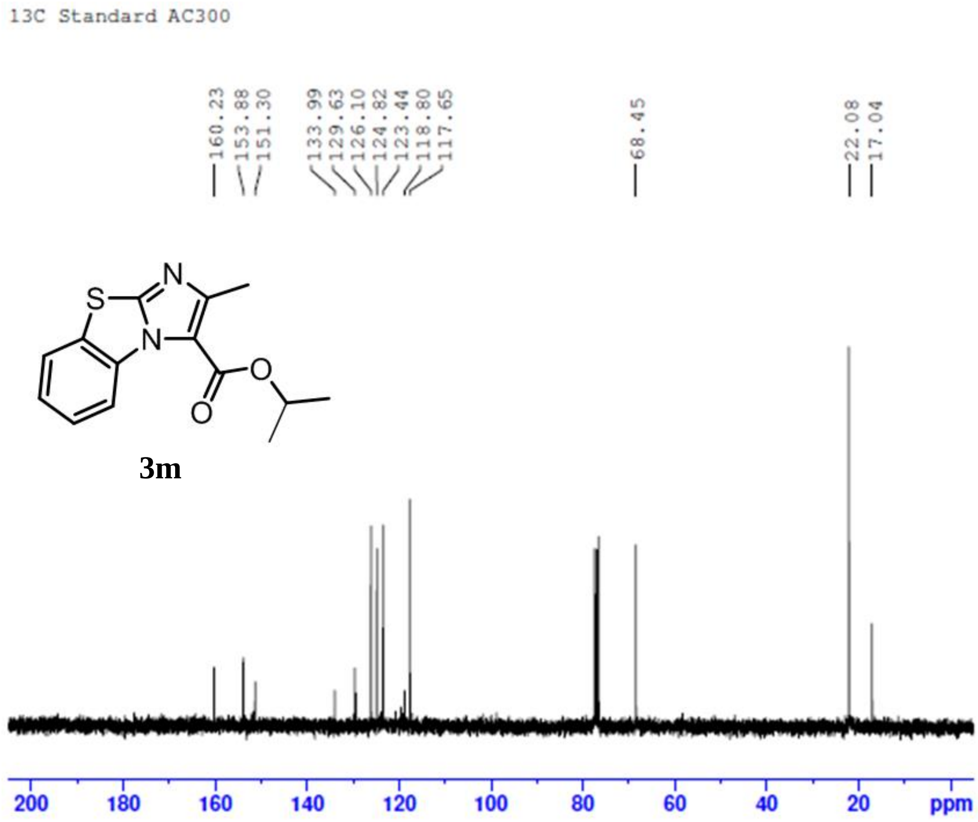
13C Standard AC300
160.23
153.88
151.30
133.99
129.63
126.10
124.82
123.44
118.80
117.65
68.45
22.08
17.04
3m
200 180 160 140 120 100 80 60 40 20 ppm

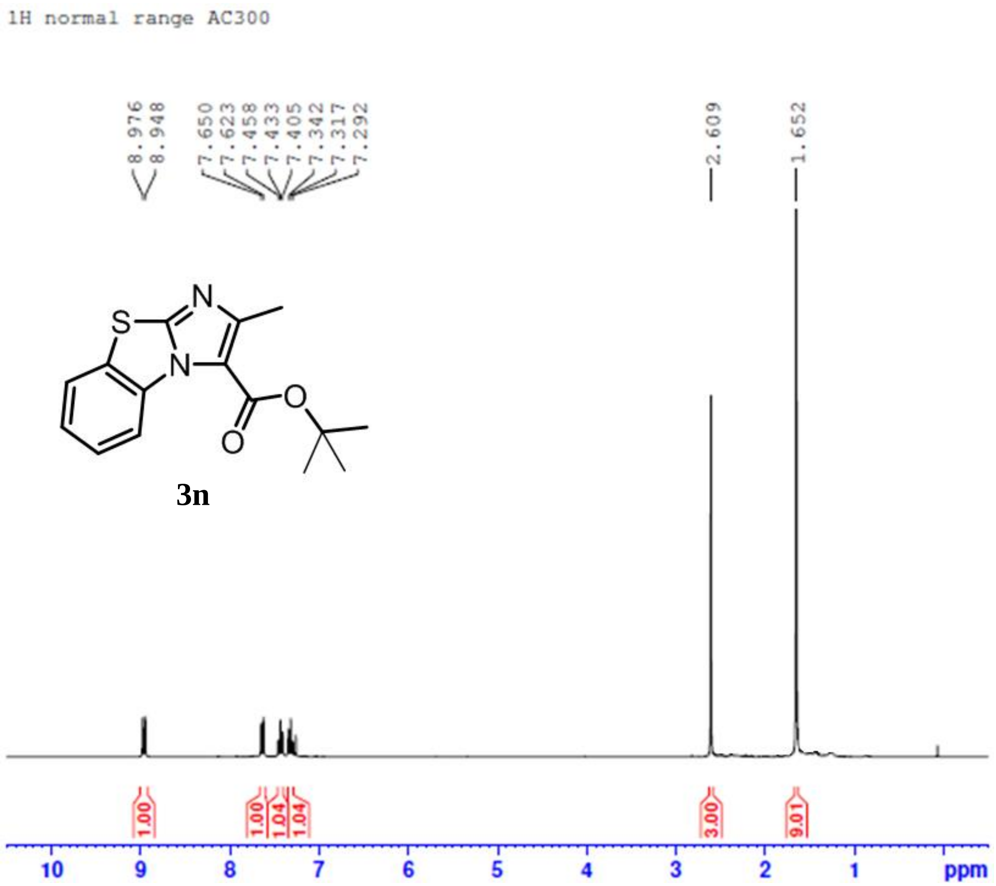
1H normal range AC300
8.976
8.948
7.650
7.623
7.458
7.433
7.405
7.342
7.317
7.292
2.609
1.652
S
N
N
O
O
3n
1.00
1.00
1.04
1.04
3.00
9.01
10 9 8 7 6 5 4 3 2 1 ppm

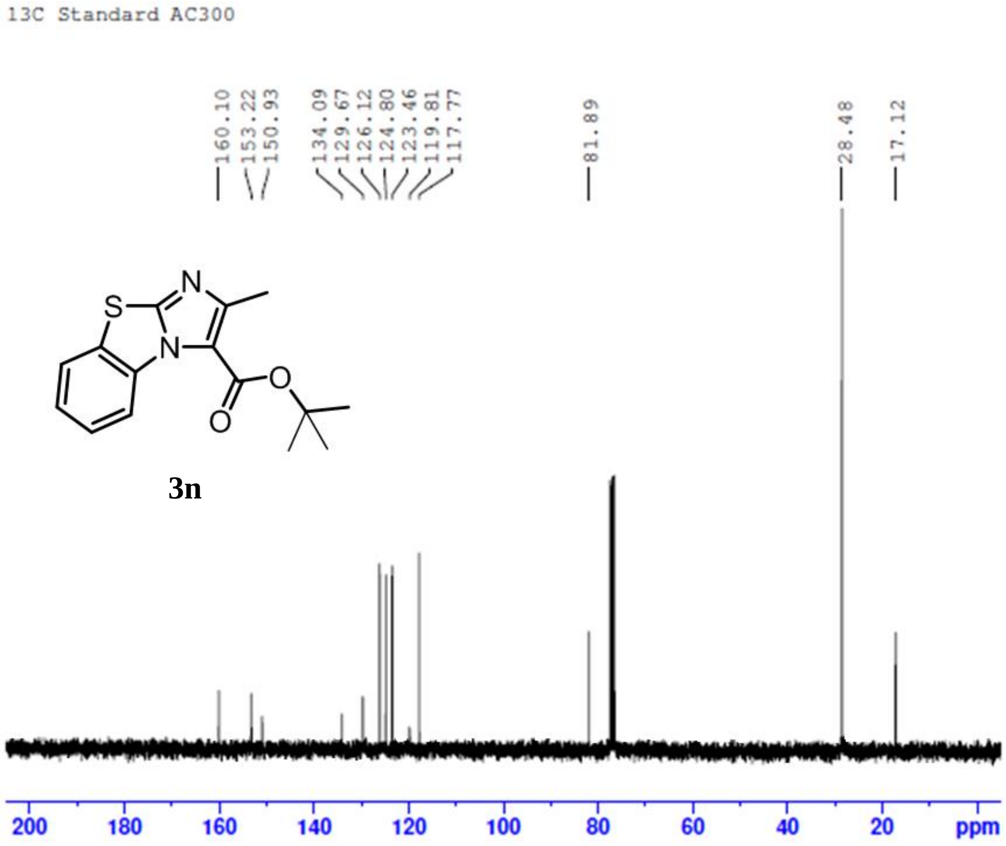
13C Standard AC300
160.10
153.22
150.93
134.09
129.67
126.12
124.80
123.46
119.81
117.77
81.89
28.48
17.12
S
N
N
O
O
3n
200 180 160 140 120 100 80 60 40 20 ppm

1H normal range AC300

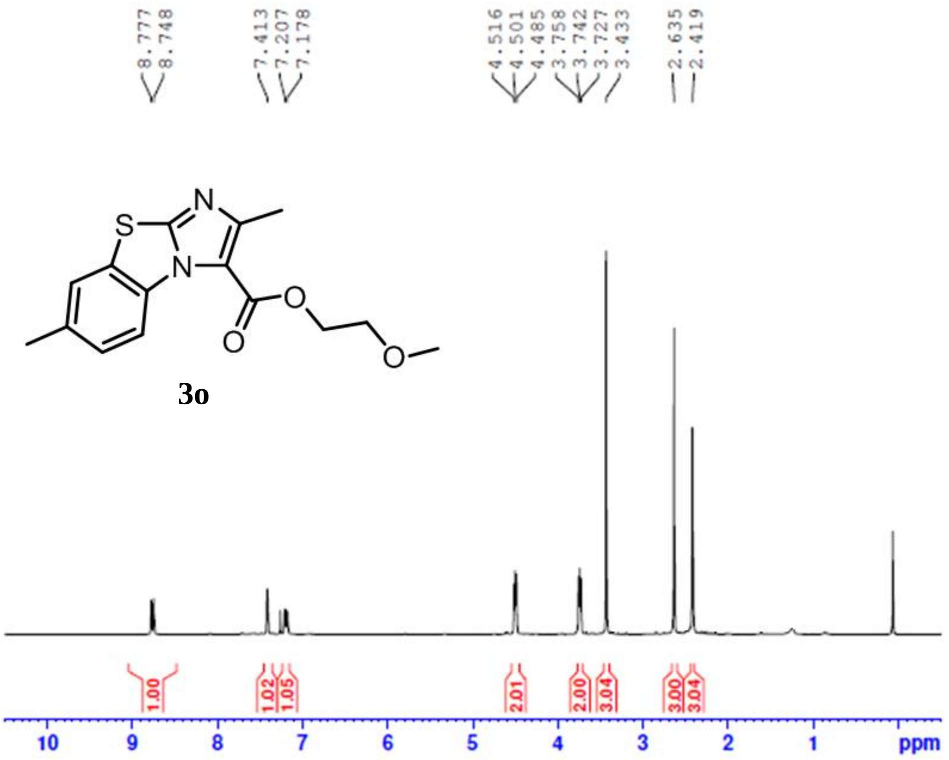

13C Standard AC300

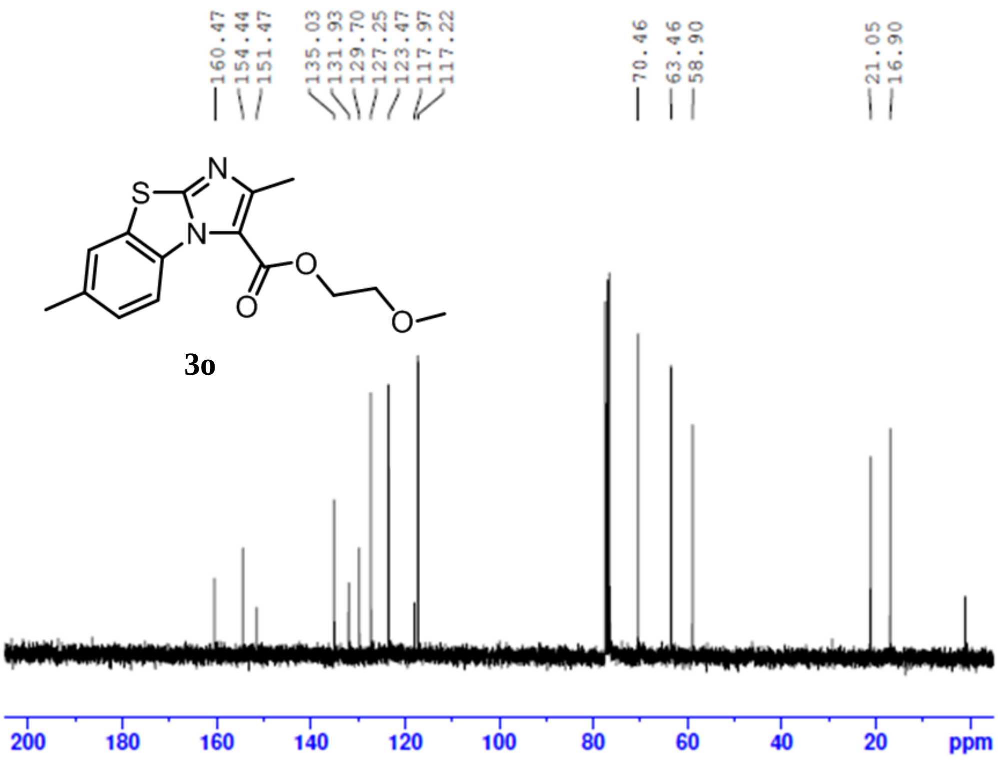

1H normal range AC300

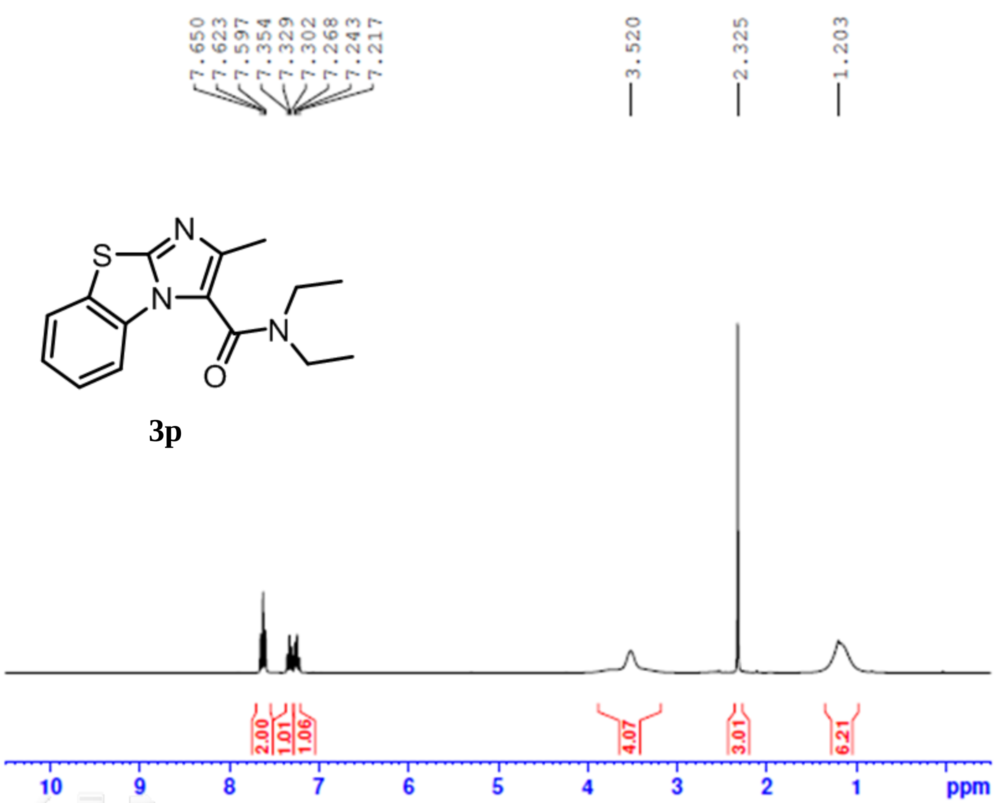

13C Standard AC300

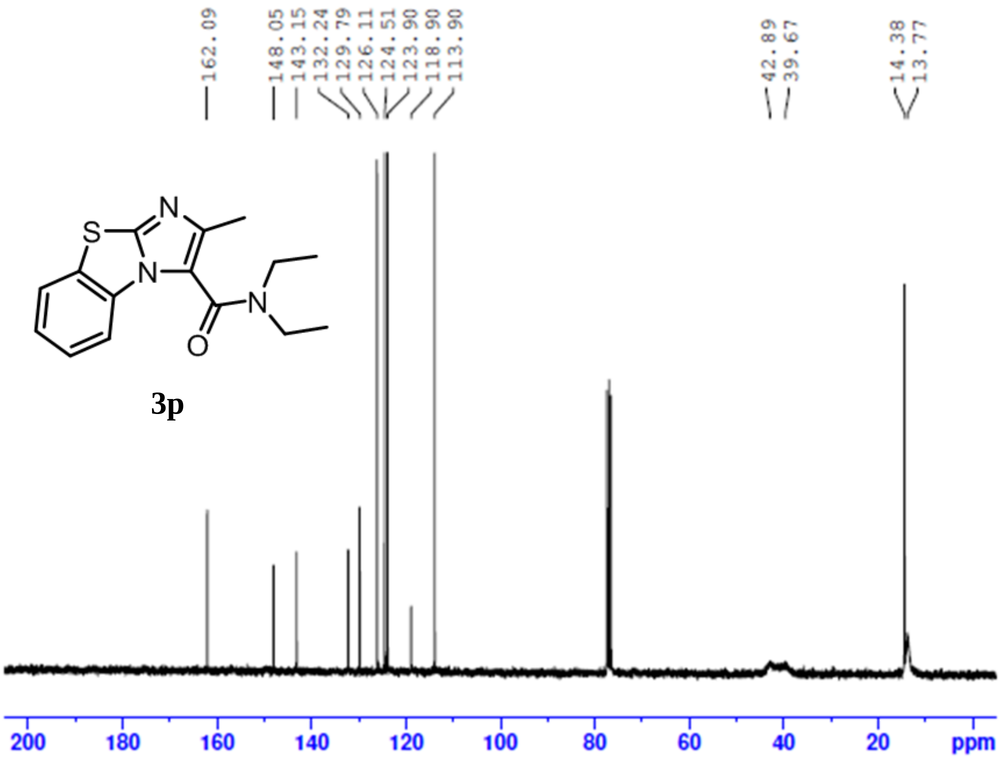

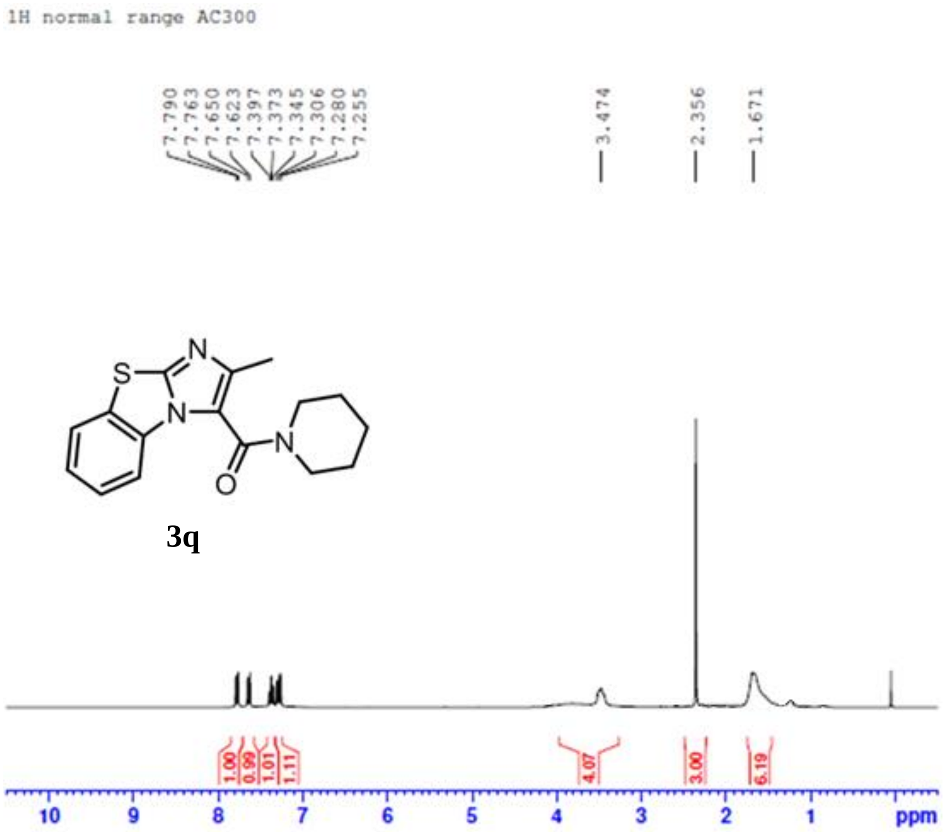
1H normal range AC300
7.790
7.763
7.650
7.623
7.397
7.373
7.345
7.306
7.280
7.255
3.474
2.356
1.671
3q
1.00
0.99
1.01
1.11
4.07
3.00
6.19
10
9
8
7
6
5
4
3
2
1
ppm

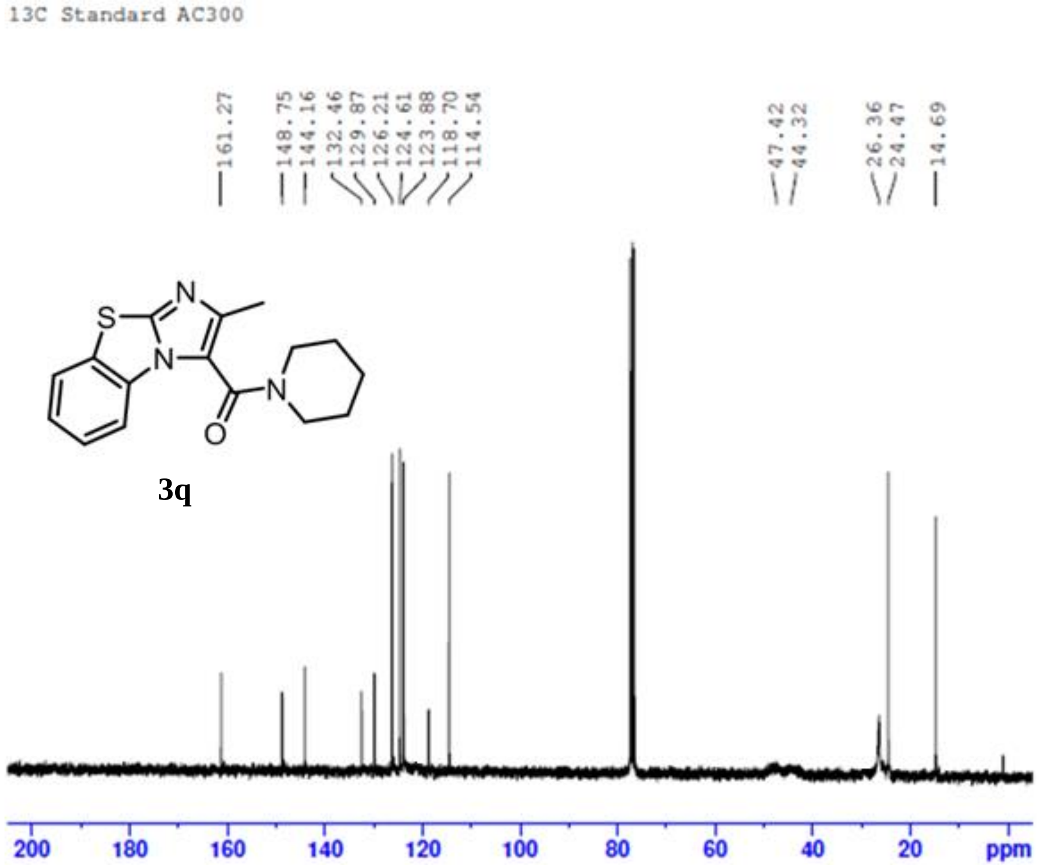
13C Standard AC300
161.27
148.75
144.16
132.46
129.87
126.21
124.61
123.88
118.70
114.54
47.42
44.32
26.36
24.47
14.69
3q
200
180
160
140
120
100
80
60
40
20
ppm

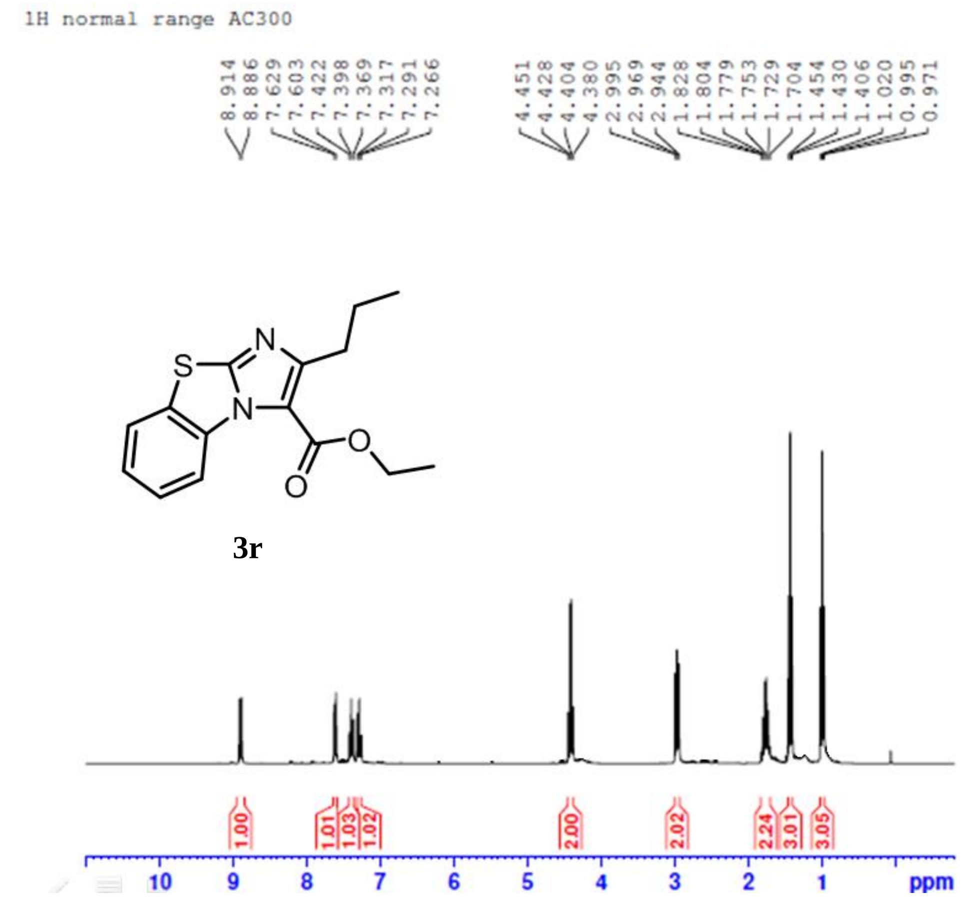
1H normal range AC300
8.914
8.886
7.629
7.603
7.422
7.398
7.369
7.317
7.291
7.266
4.451
4.428
4.404
4.380
2.995
2.969
2.944
1.828
1.804
1.779
1.753
1.729
1.704
1.454
1.430
1.406
1.020
0.995
0.971
S
N
N
O
O
3r
1.00
1.01
1.03
1.02
2.00
2.02
2.24
3.01
3.05
10
9
8
7
6
5
4
3
2
1
ppm

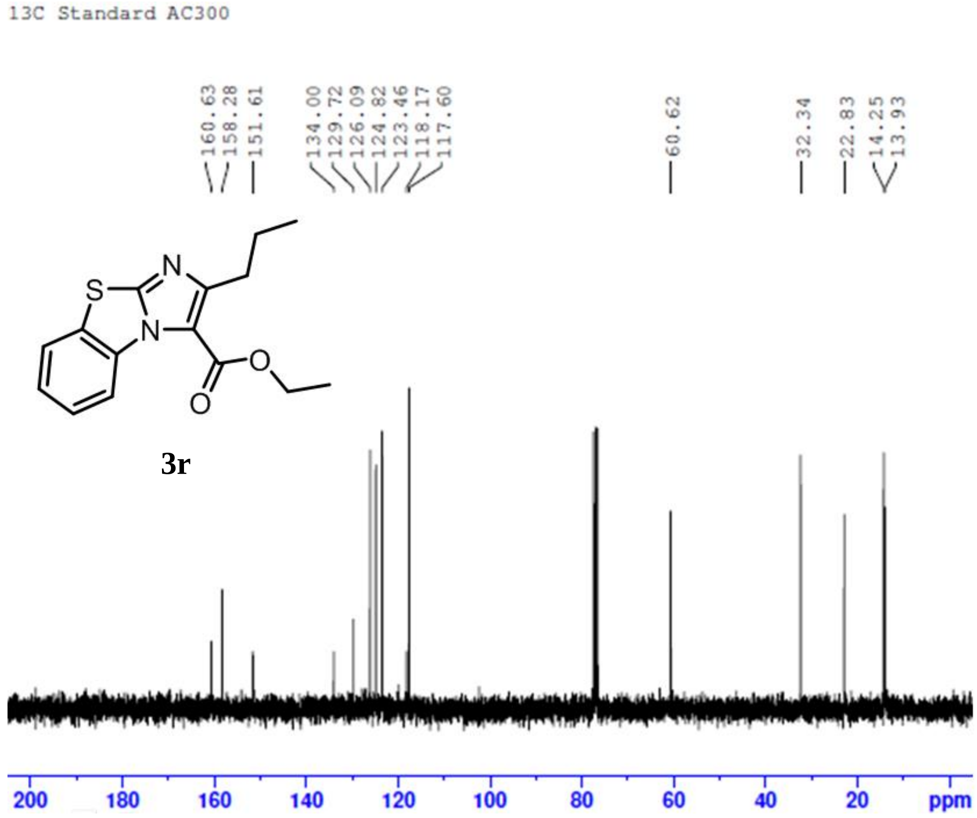
13C Standard AC300
160.63
158.28
151.61
134.00
129.72
126.09
124.82
123.46
118.17
117.60
60.62
32.34
22.83
14.25
13.93
S
N
N
O
O
3r
200
180
160
140
120
100
80
60
40
20
ppm

1H normal range AC300

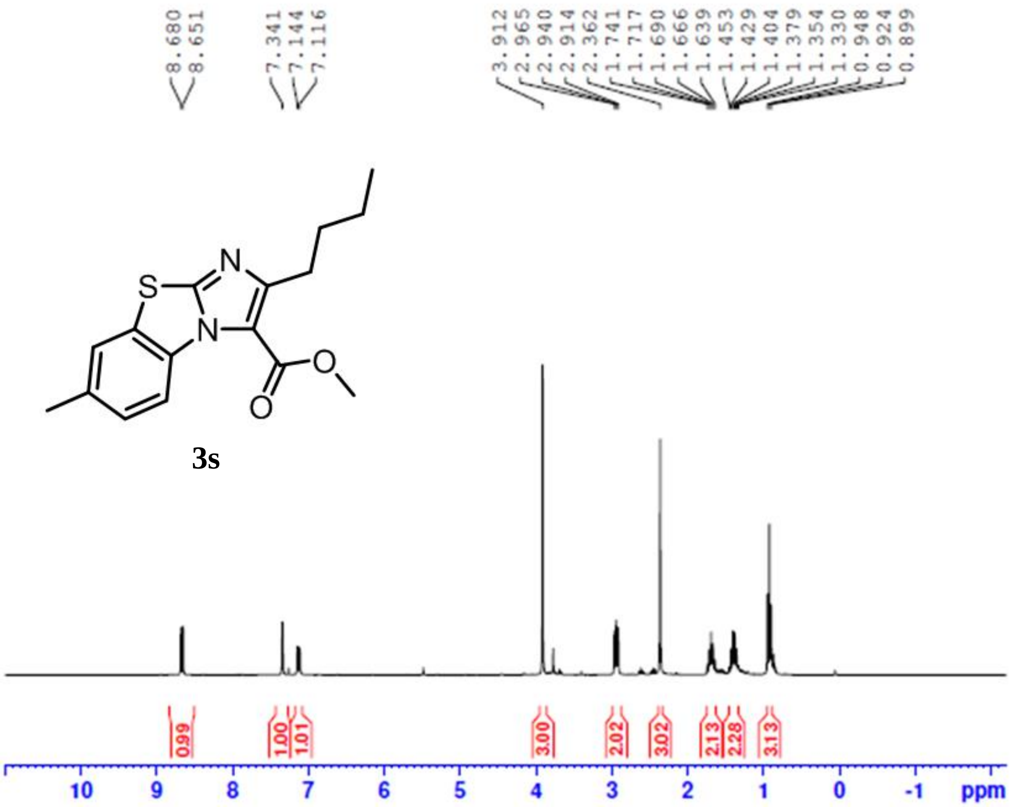

13C Standard AC300

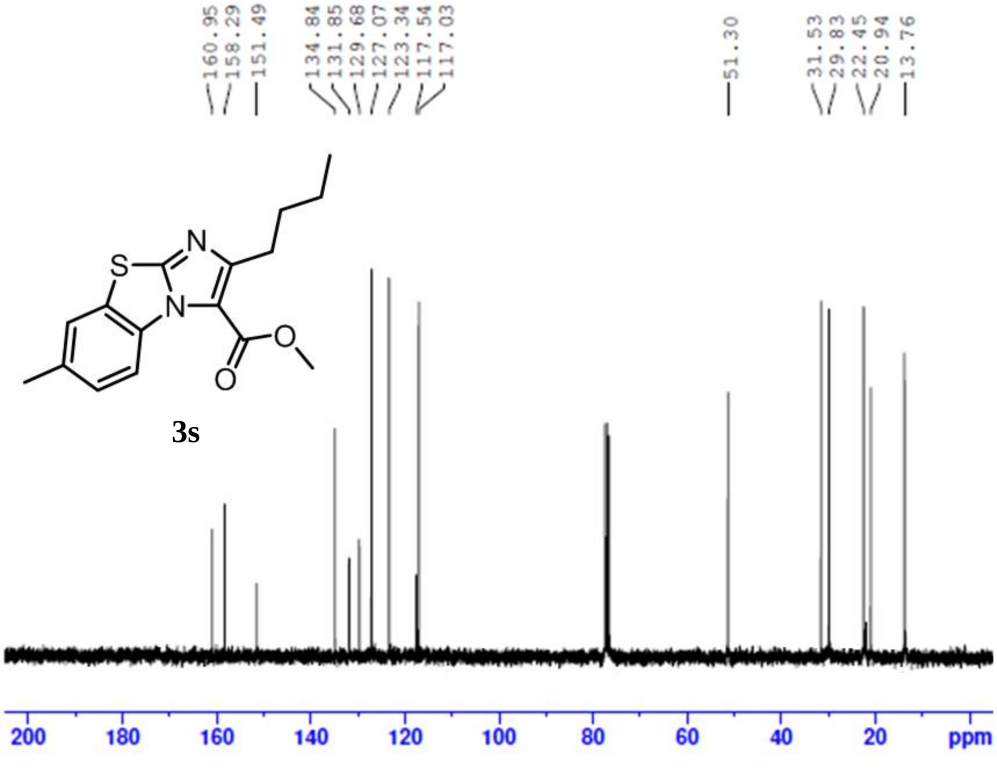

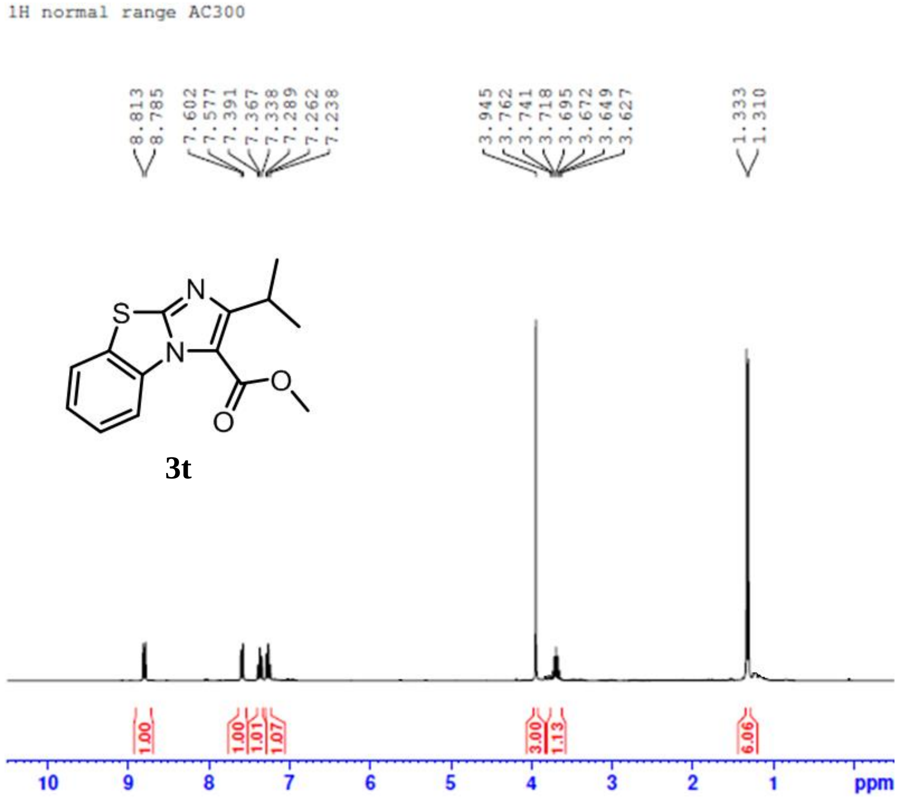
1H normal range AC300
8.813
8.785
7.602
7.577
7.391
7.367
7.338
7.289
7.262
7.238
3.945
3.762
3.741
3.718
3.695
3.672
3.649
3.627
1.333
1.310
S
N
N
O
O
3t
1.00
1.00
1.01
1.07
3.00
1.13
6.06
10
9
8
7
6
5
4
3
2
1
ppm

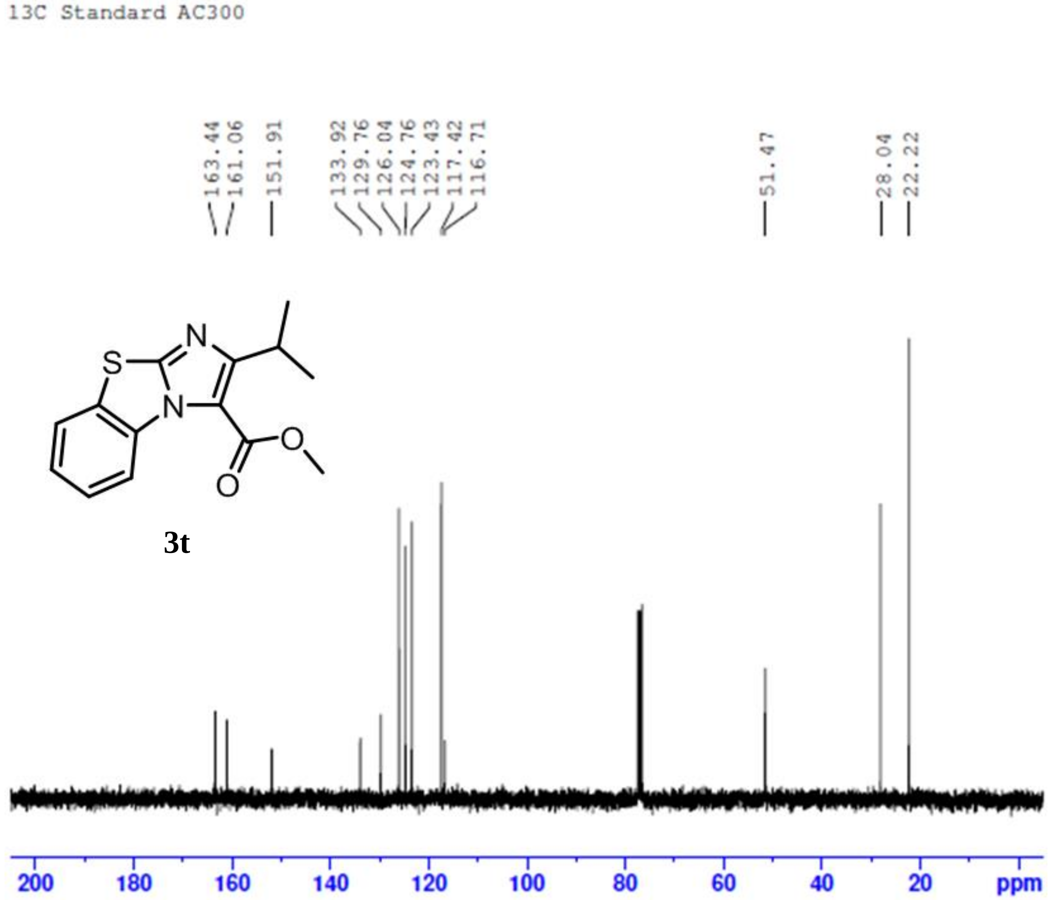
13C Standard AC300
163.44
161.06
151.91
133.92
129.76
126.04
124.76
123.43
117.42
116.71
51.47
28.04
22.22
S
N
N
O
O
3t
200
180
160
140
120
100
80
60
40
20
ppm

1H normal range AC300

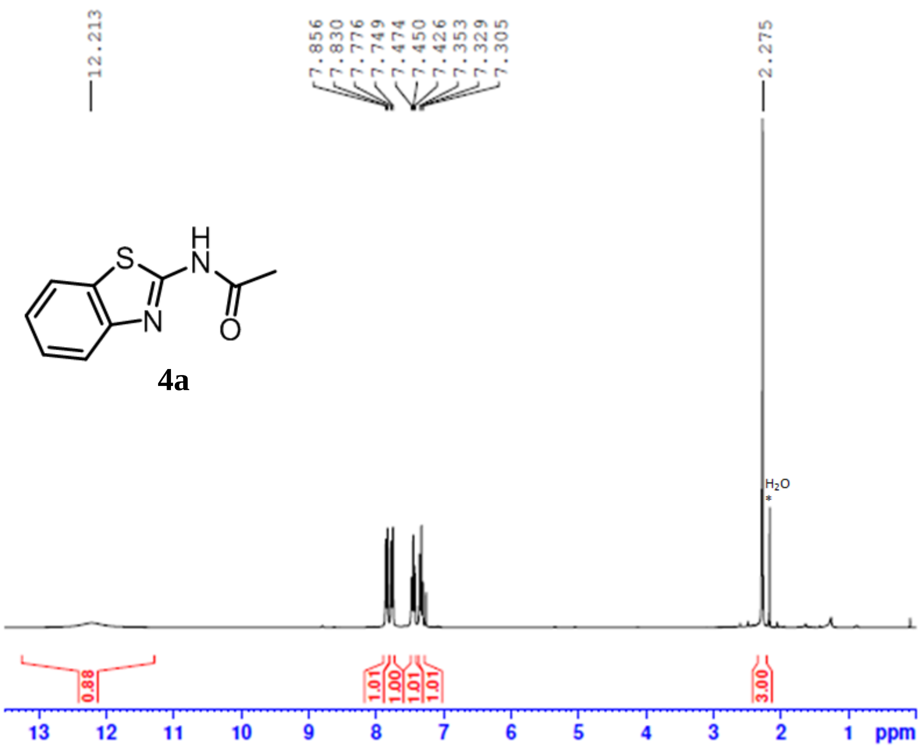

13C Standard AC300

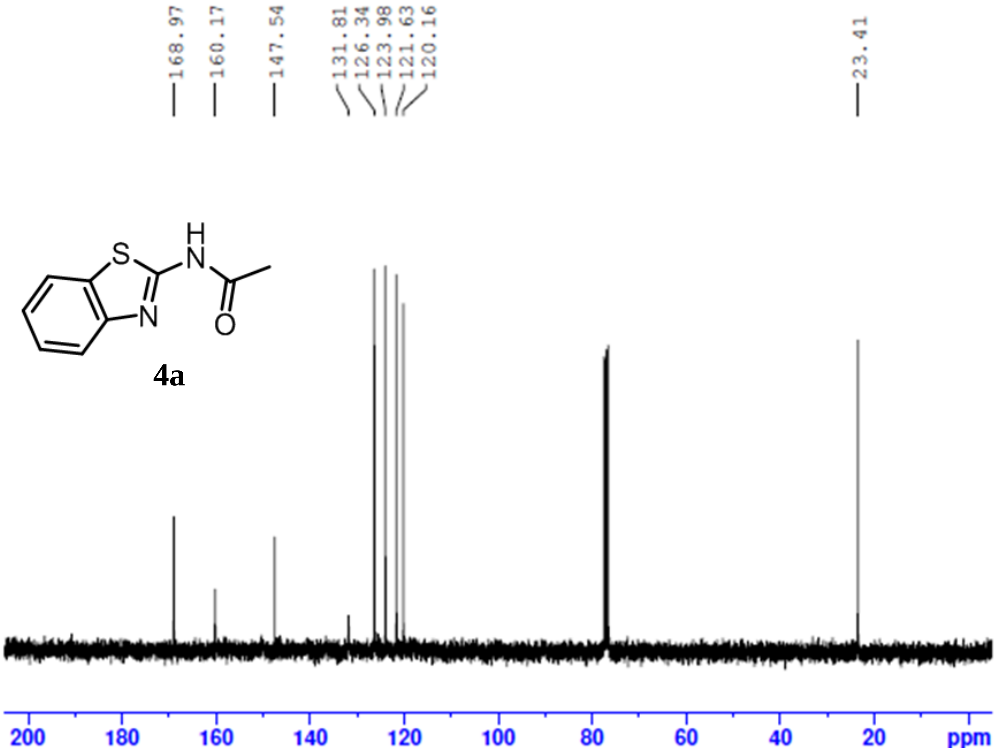

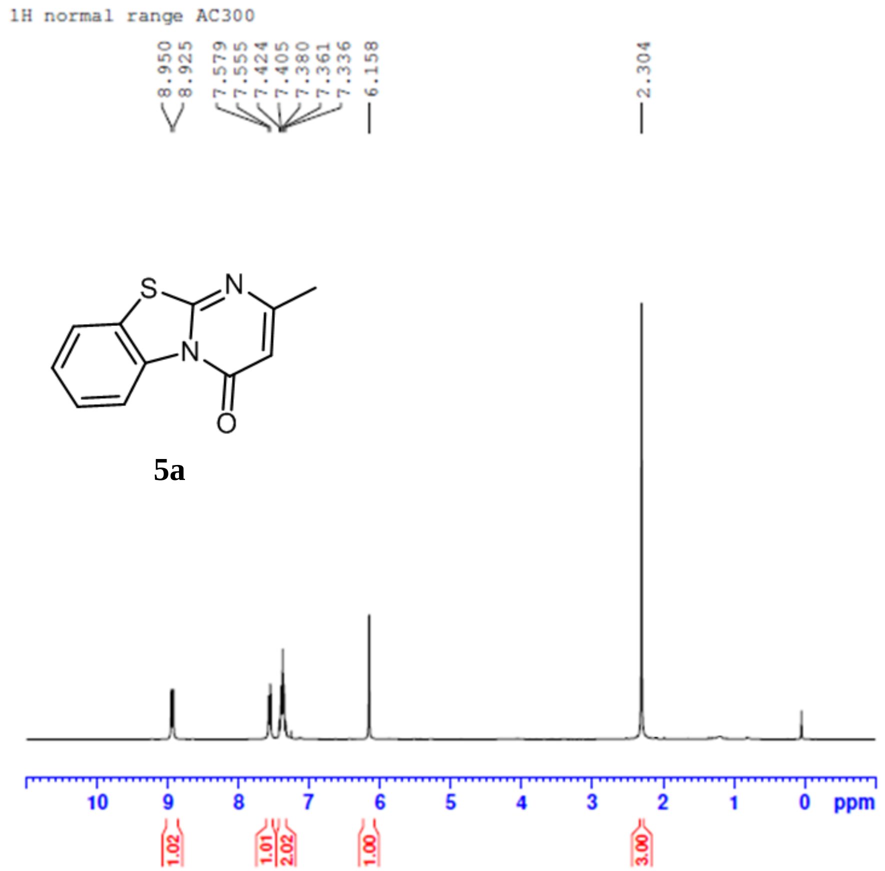
1H normal range AC300
8.950
8.925
7.579
7.555
7.424
7.405
7.380
7.361
7.336
6.158
2.304
S
N
N
O
5a
10
9
8
7
6
5
4
3
2
1
0
ppm
1.02
1.01
2.02
1.00
3.00

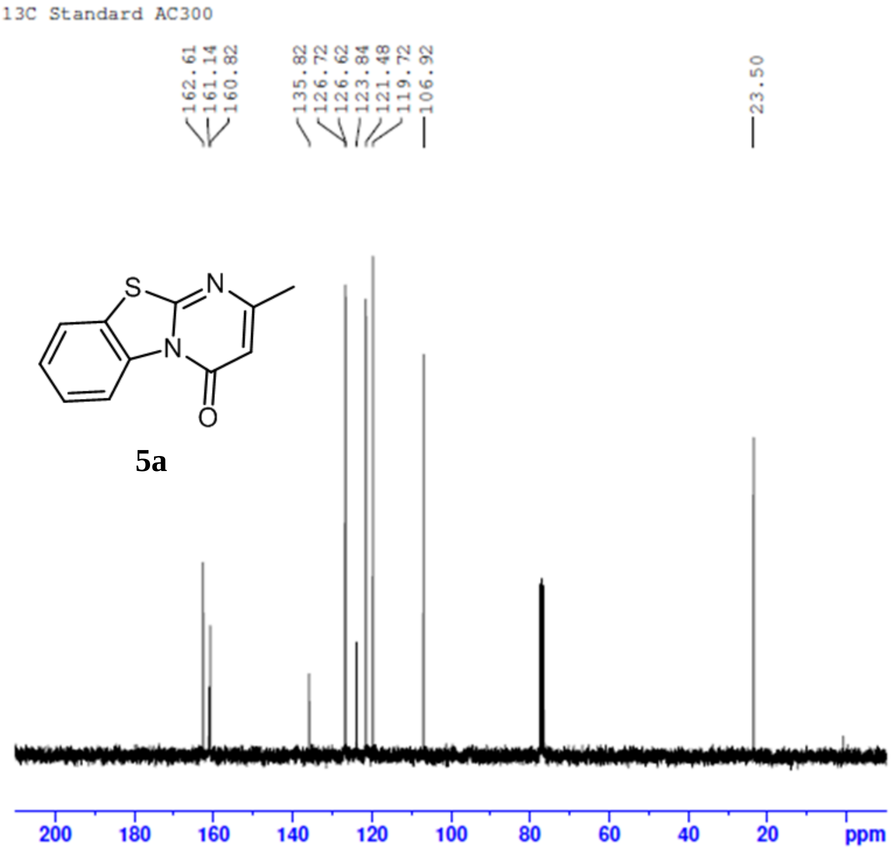
13C Standard AC300
162.61
161.14
160.82
135.82
126.72
126.62
123.84
121.48
119.72
106.92
23.50
S
N
N
O
5a
200
180
160
140
120
100
80
60
40
20
ppm

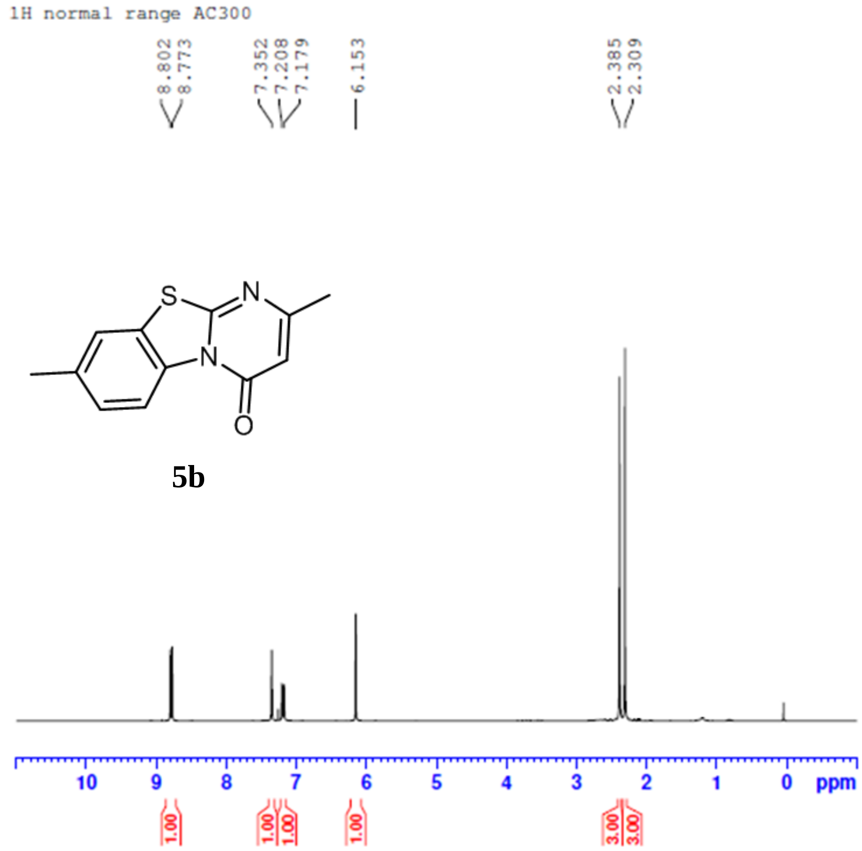
1H normal range AC300
8.802
8.773
7.352
7.208
7.179
6.153
2.385
2.309
S
N
N
O
5b
10
9
8
7
6
5
4
3
2
1
0
ppm
1.00
1.00
1.00
1.00
3.00
3.00

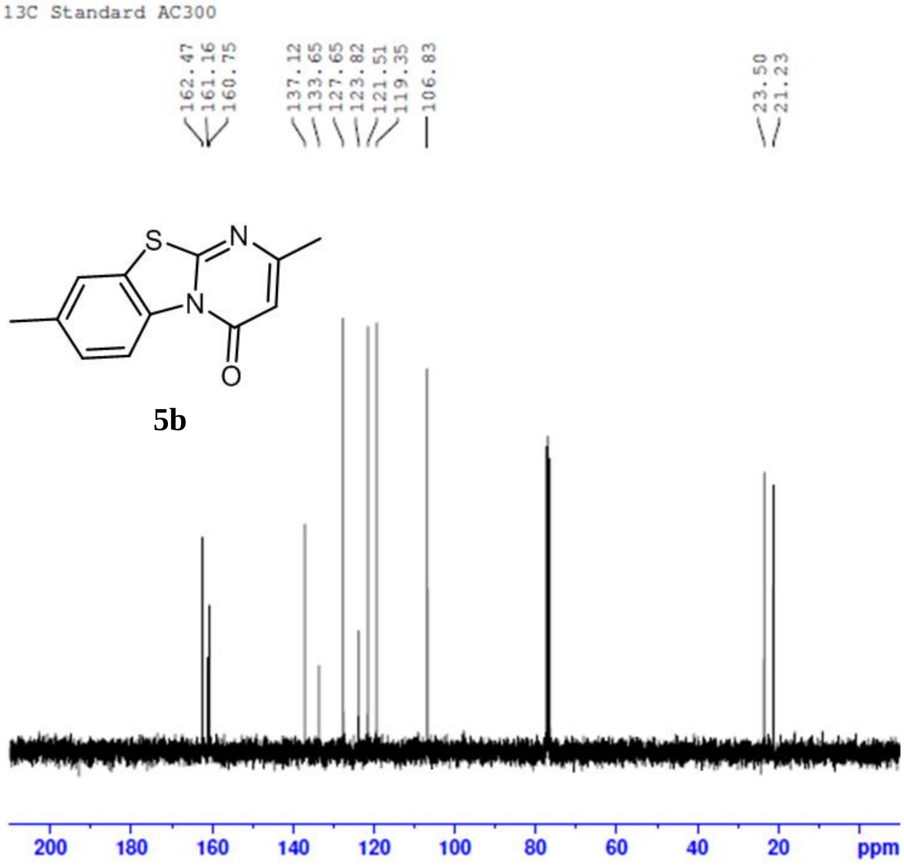
13C Standard AC300
162.47
161.16
160.75
137.12
133.65
127.65
123.82
121.51
119.35
106.83
23.50
21.23
S
N
N
O
5b
200
180
160
140
120
100
80
60
40
20
ppm

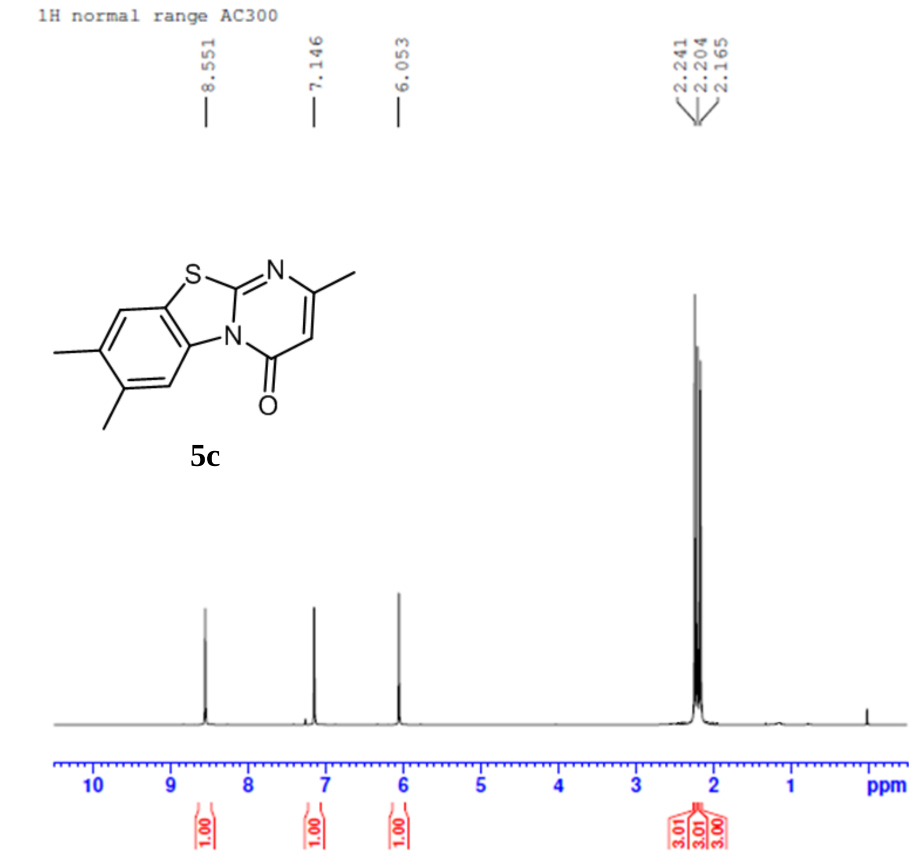
1H normal range AC300
8.551
7.146
6.053
2.241
2.204
2.165
5c
10 9 8 7 6 5 4 3 2 1 ppm
1.00
1.00
1.00
3.01
3.01
3.00

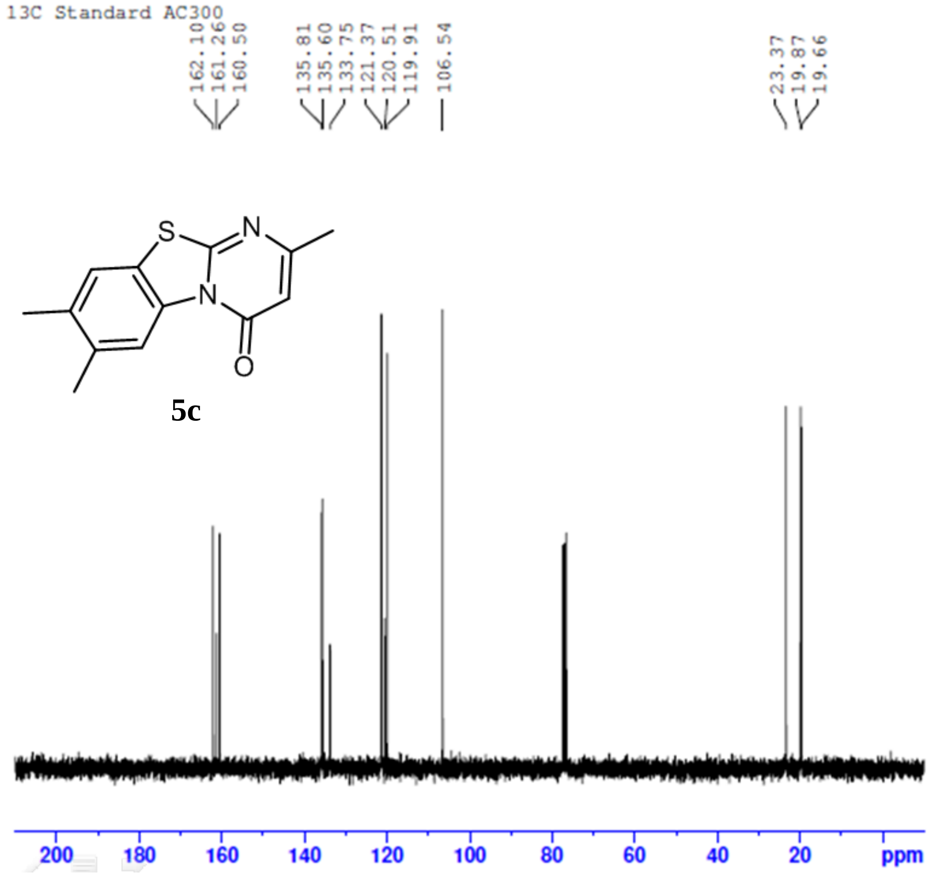
13C Standard AC300
162.10
161.26
160.50
135.81
135.60
133.75
121.37
120.51
119.91
106.54
23.37
19.87
19.66
5c
200 180 160 140 120 100 80 60 40 20 ppm

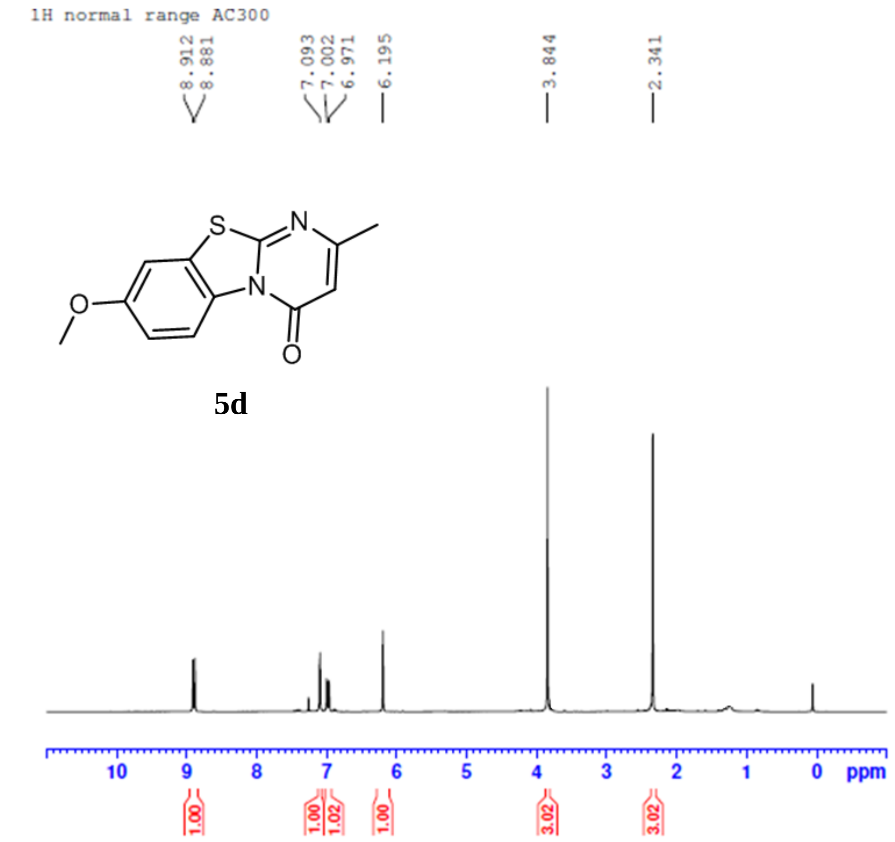
1H normal range AC300
8.912
8.881
7.093
7.002
6.971
6.195
3.844
2.341
5d
10
9
8
7
6
5
4
3
2
1
0
ppm
1.00
1.00
1.02
1.00
3.02
3.02

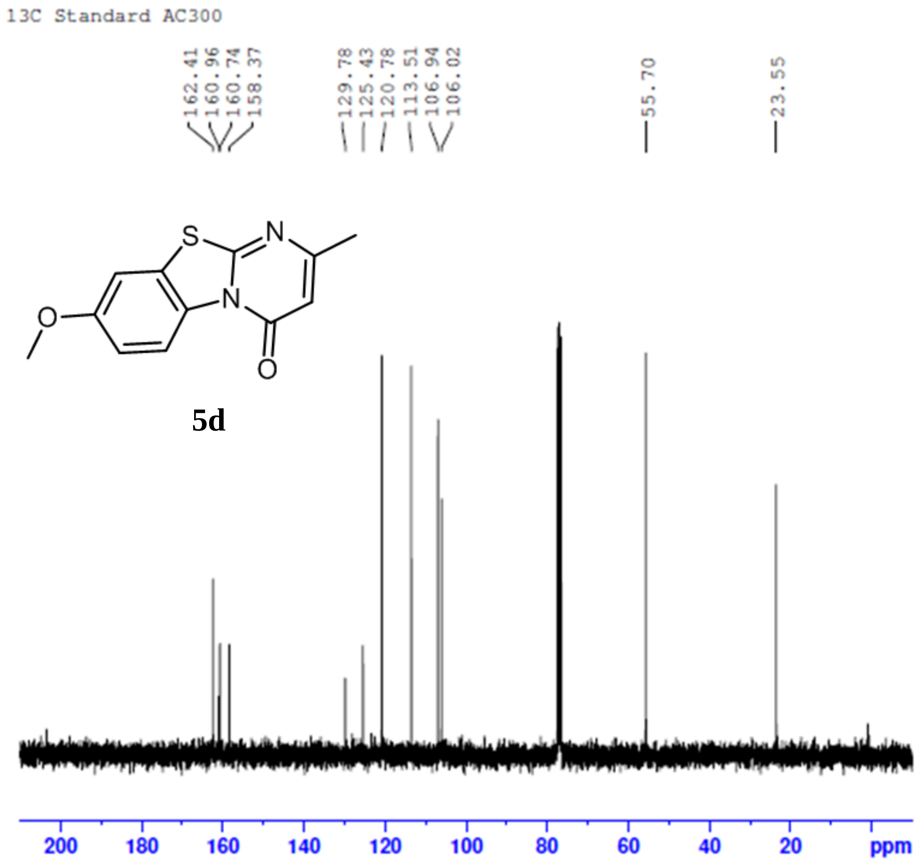
13C Standard AC300
162.41
160.96
160.74
158.37
129.78
125.43
120.78
113.51
106.94
106.02
55.70
23.55
5d
200
180
160
140
120
100
80
60
40
20
ppm

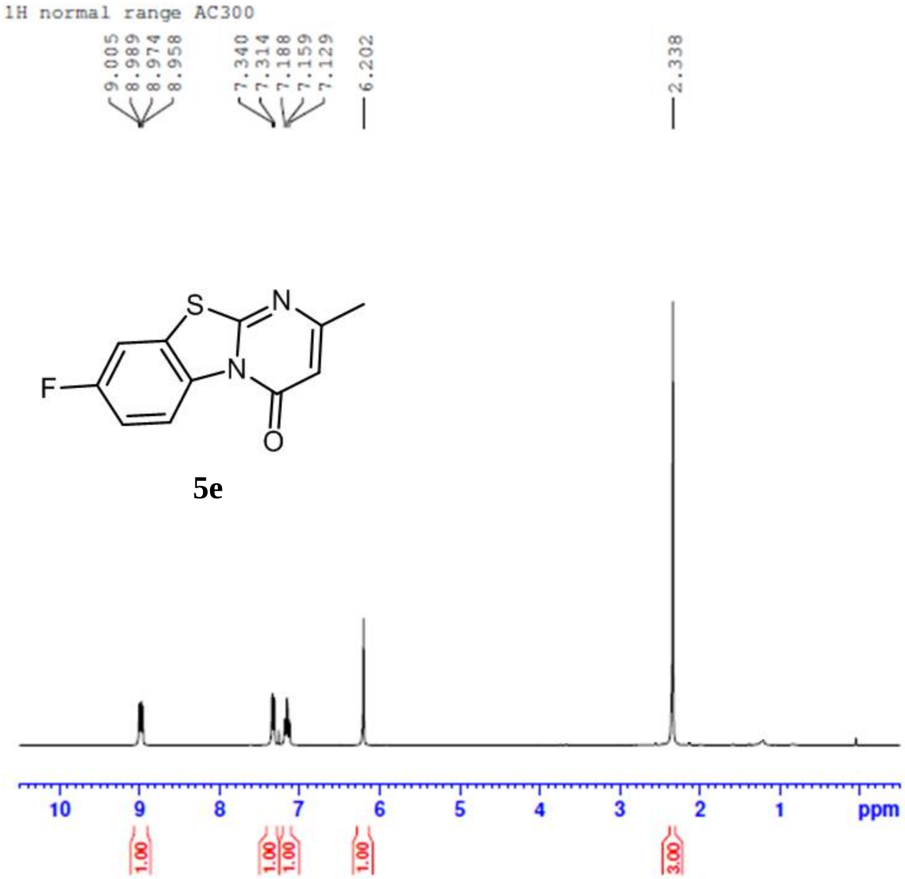
1H normal range AC300
9.005
8.989
8.974
8.958
7.340
7.314
7.188
7.159
7.129
6.202
2.338
F
S
N
N
O
5e
10
9
8
7
6
5
4
3
2
1
ppm
1.00
1.00
1.00
1.00
3.00

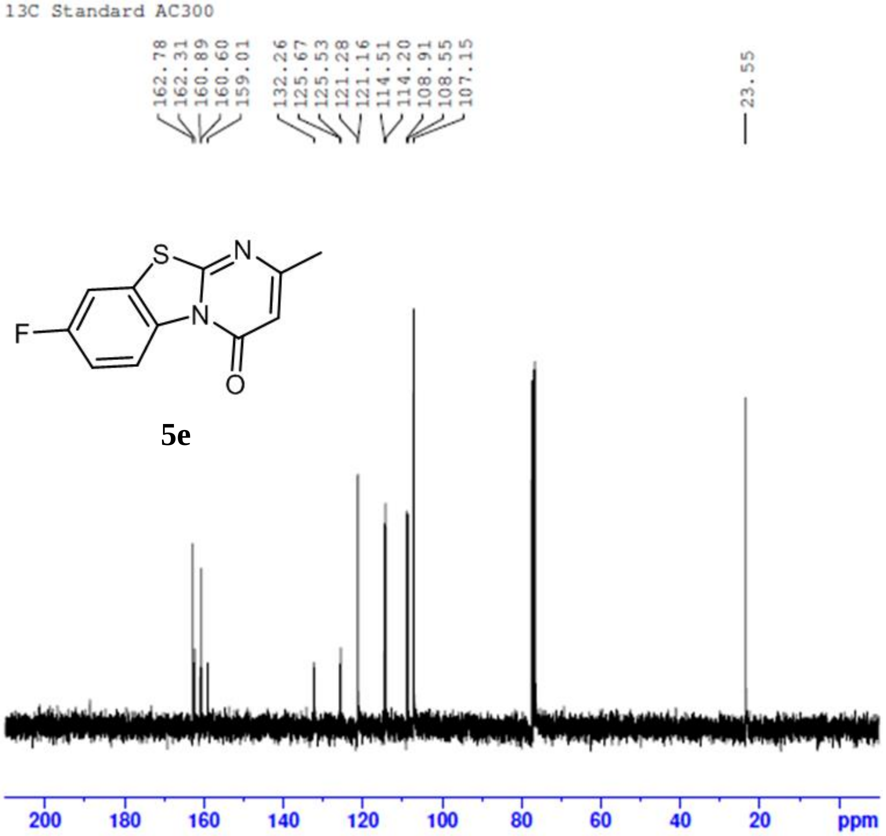
13C Standard AC300
162.78
162.31
160.89
160.60
159.01
132.26
125.67
125.53
121.28
121.16
114.51
114.20
108.91
108.55
107.15
23.55
F
S
N
N
O
5e
200
180
160
140
120
100
80
60
40
20
ppm

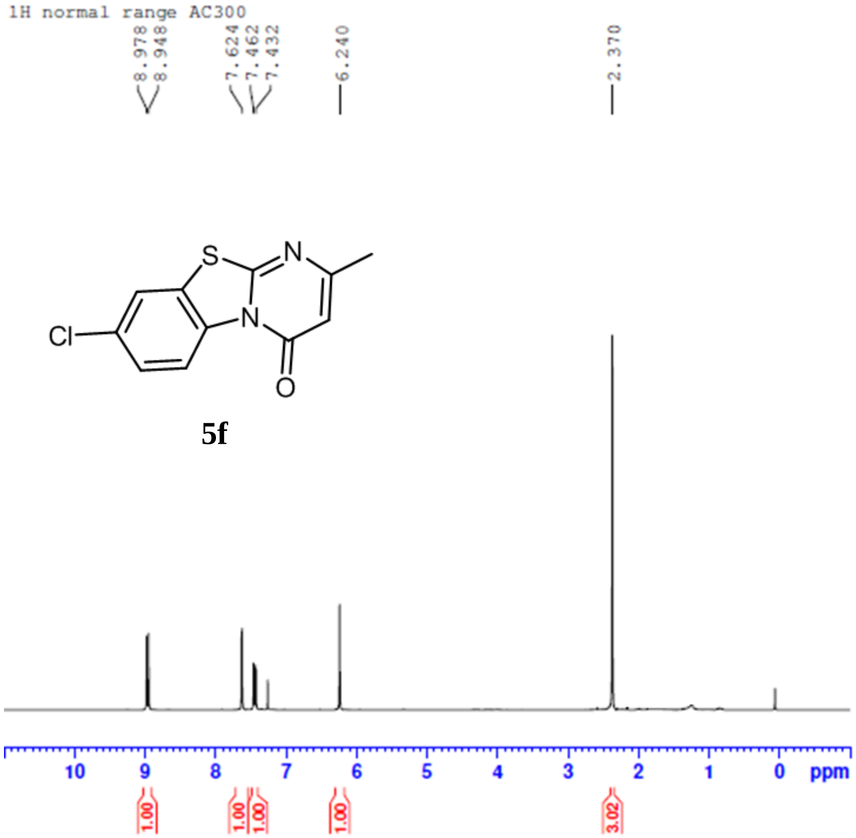
1H normal range AC300
8.978
8.948
7.624
7.462
7.432
6.240
2.370
S
N
Cl
N
O
5f
10 9 8 7 6 5 4 3 2 1 0 ppm
1.00
1.00
1.00
1.00
3.02

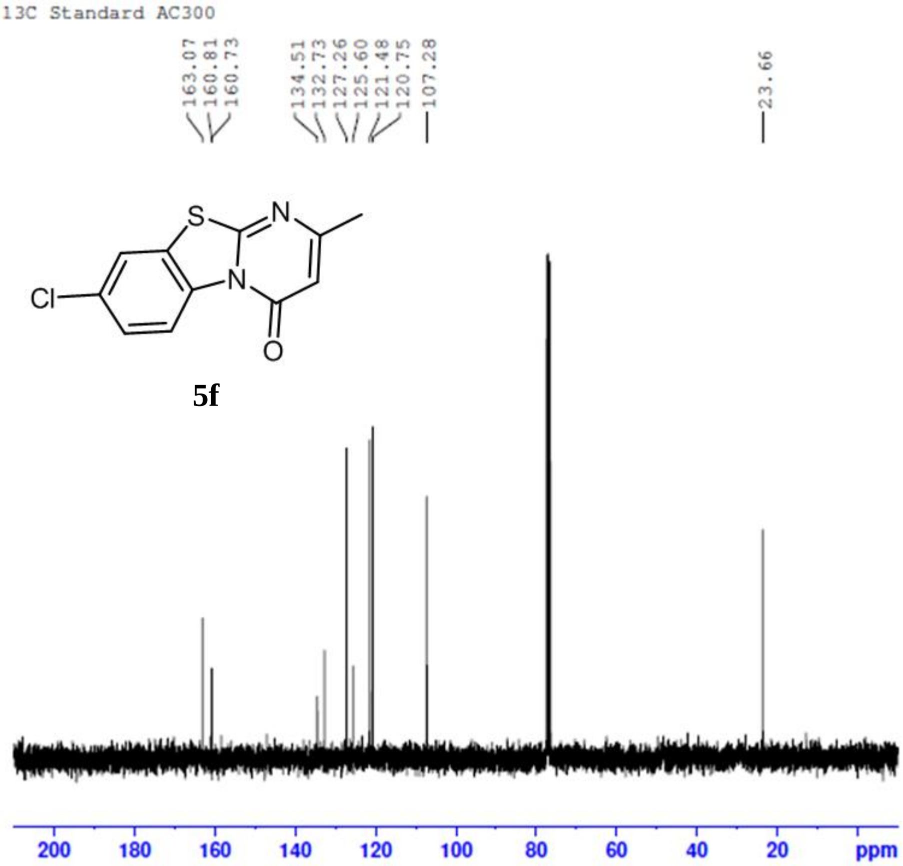
13C Standard AC300
163.07
160.81
160.73
134.51
132.73
127.26
125.60
121.48
120.75
107.28
23.66
S
N
Cl
N
O
5f
200 180 160 140 120 100 80 60 40 20 ppm

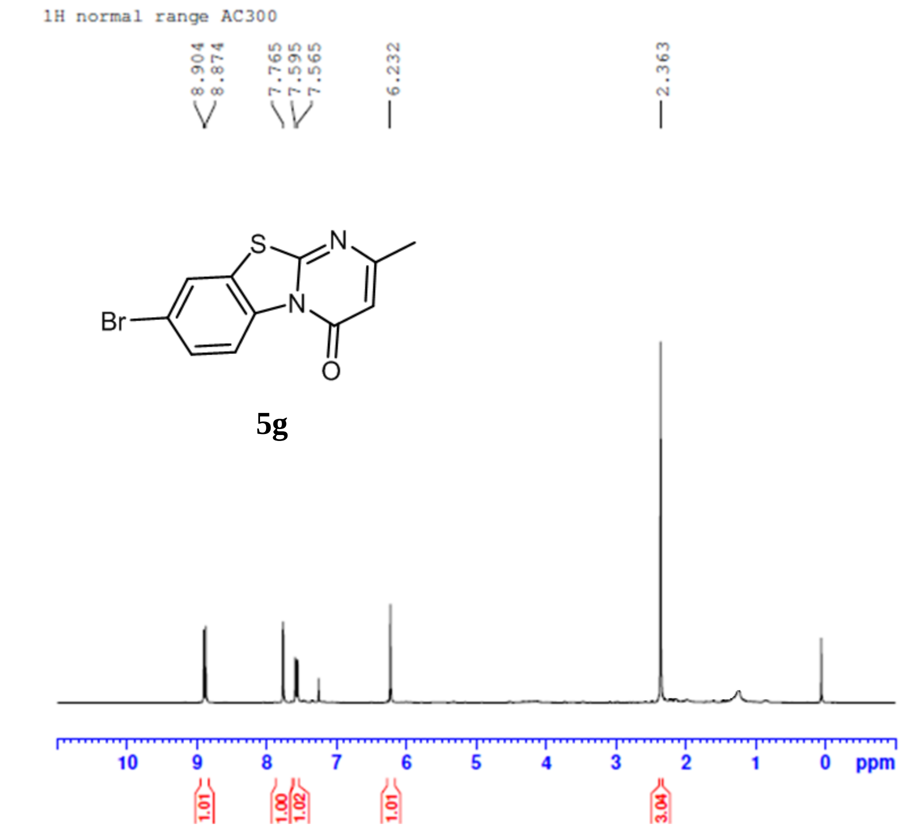
1H normal range AC300
8.904
8.874
7.765
7.595
7.565
6.232
2.363
Br
S
N
N
O
5g
10
9
8
7
6
5
4
3
2
1
0
ppm
1.01
1.00
1.02
1.01
3.04

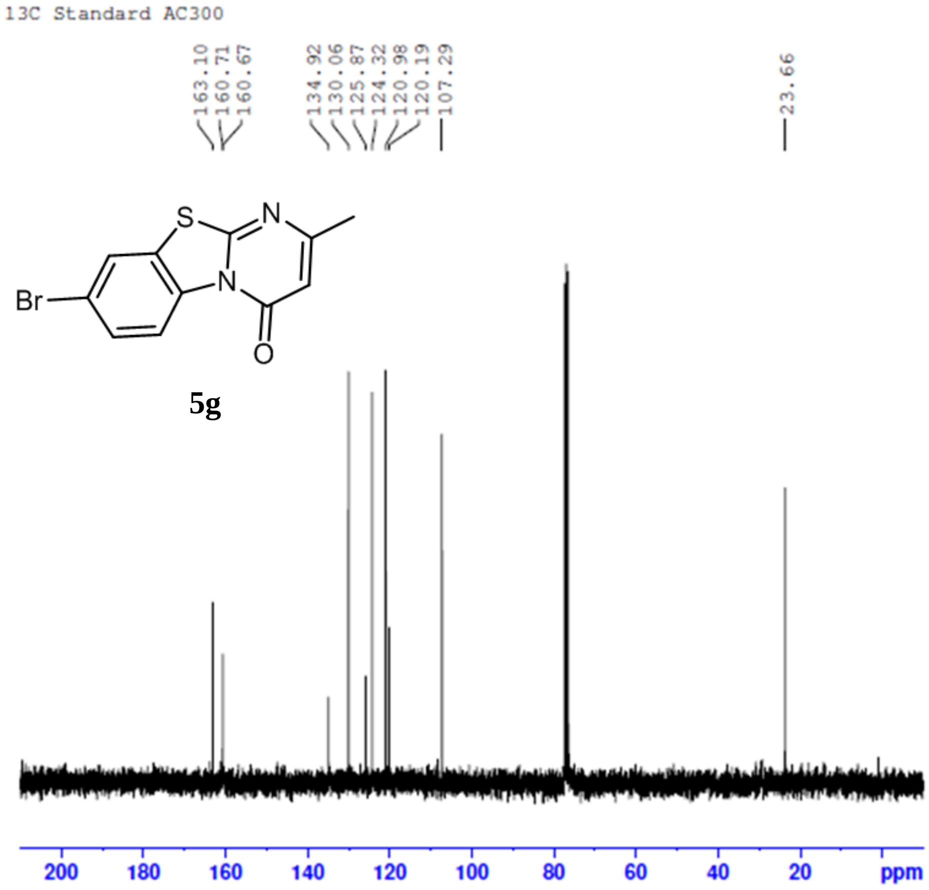
13C Standard AC300
163.10
160.71
160.67
134.92
130.06
125.87
124.32
120.98
120.19
107.29
23.66
Br
S
N
N
O
5g
200
180
160
140
120
100
80
60
40
20
ppm

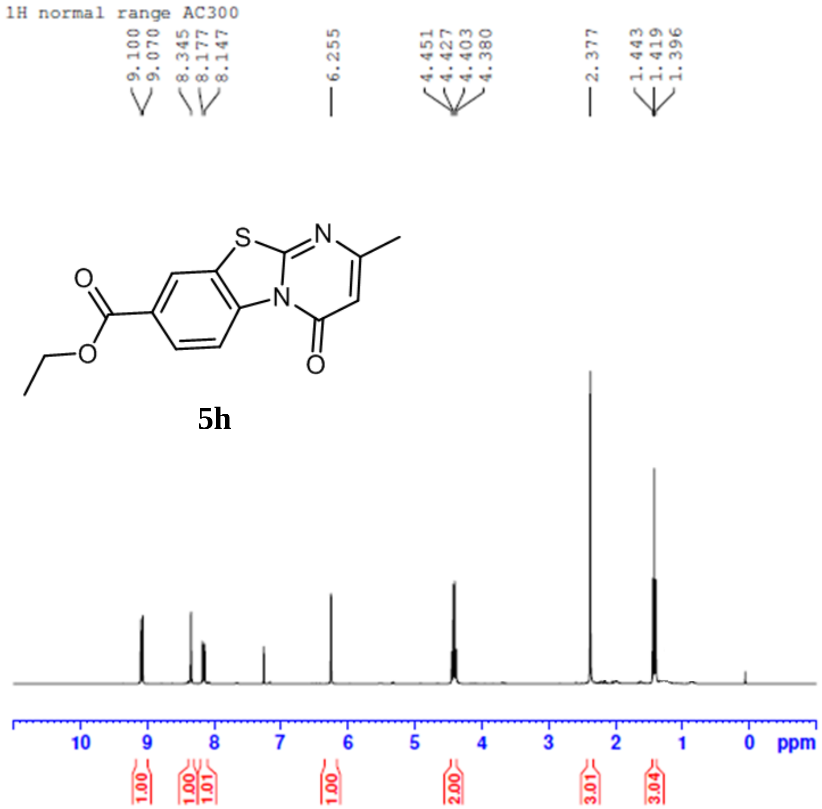
1H normal range AC300
9.100
9.070
8.345
8.177
8.147
6.255
4.451
4.427
4.403
4.380
2.377
1.443
1.419
1.396
S
N
O
O
N
O
5h
10
9
8
7
6
5
4
3
2
1
0
ppm
1.00
1.00
1.01
1.00
2.00
3.01
3.04

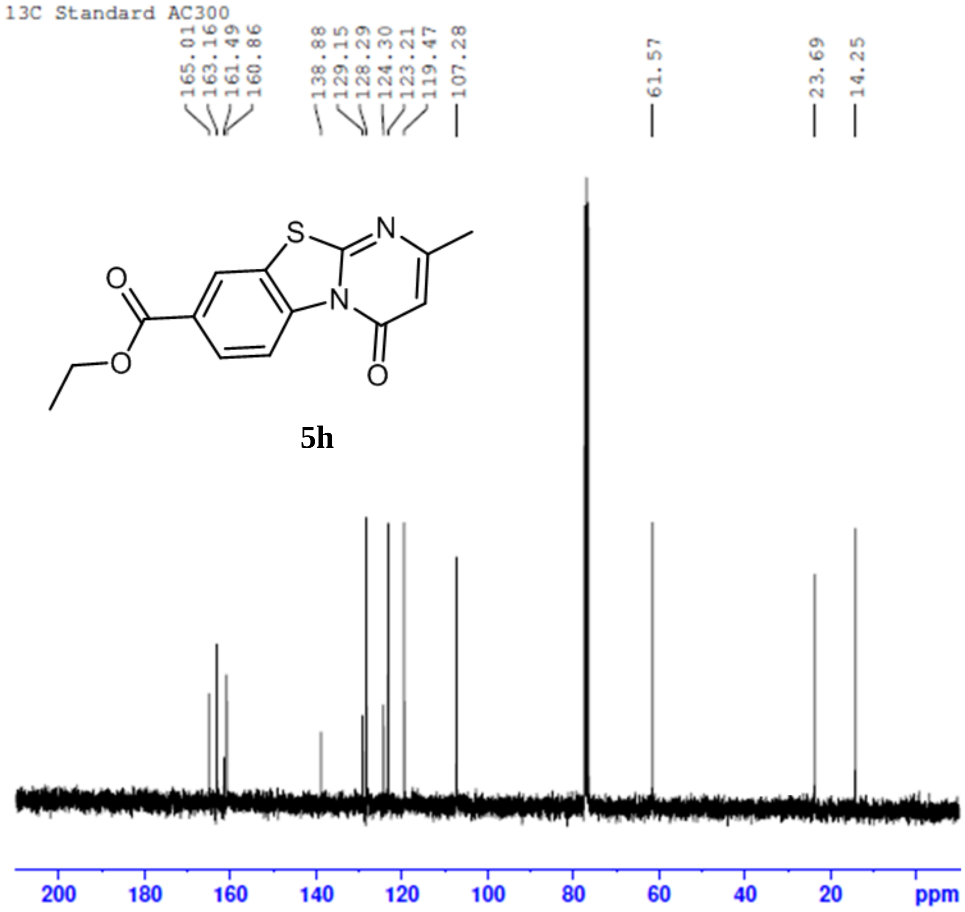
13C Standard AC300
165.01
163.16
161.49
160.86
138.88
129.15
128.29
124.30
123.21
119.47
107.28
61.57
23.69
14.25
S
N
O
O
N
O
5h
200
180
160
140
120
100
80
60
40
20
ppm

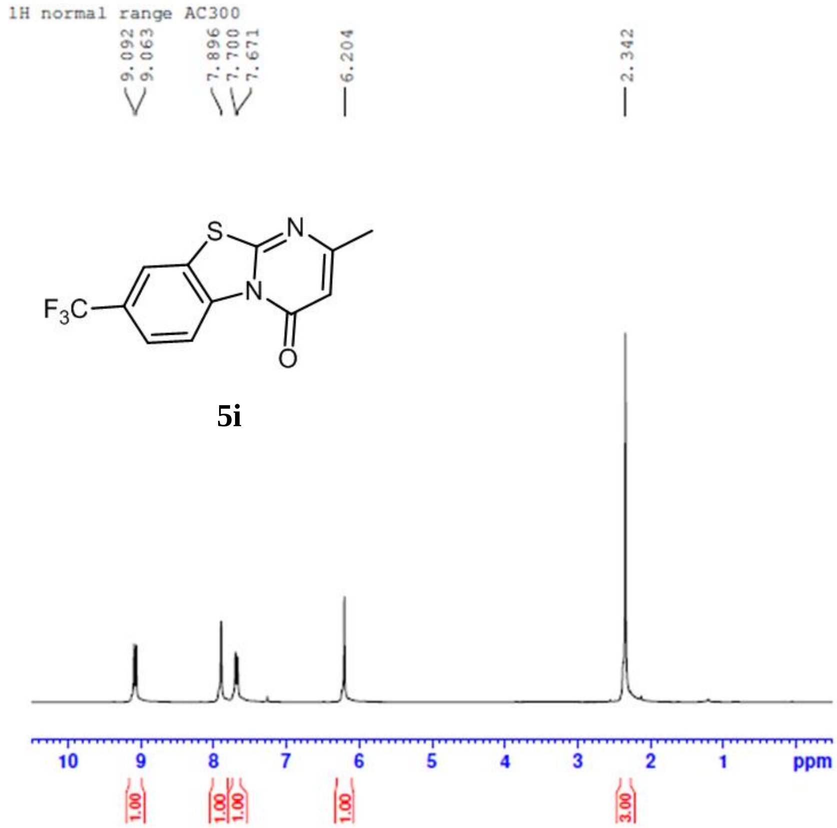

1H normal range AC300
9.092
9.063
7.896
7.700
7.671
6.204
2.342
F3C
S
N
O
10 9 8 7 6 5 4 3 2 1 ppm
1.00
1.00
1.00
1.00
3.00

5i

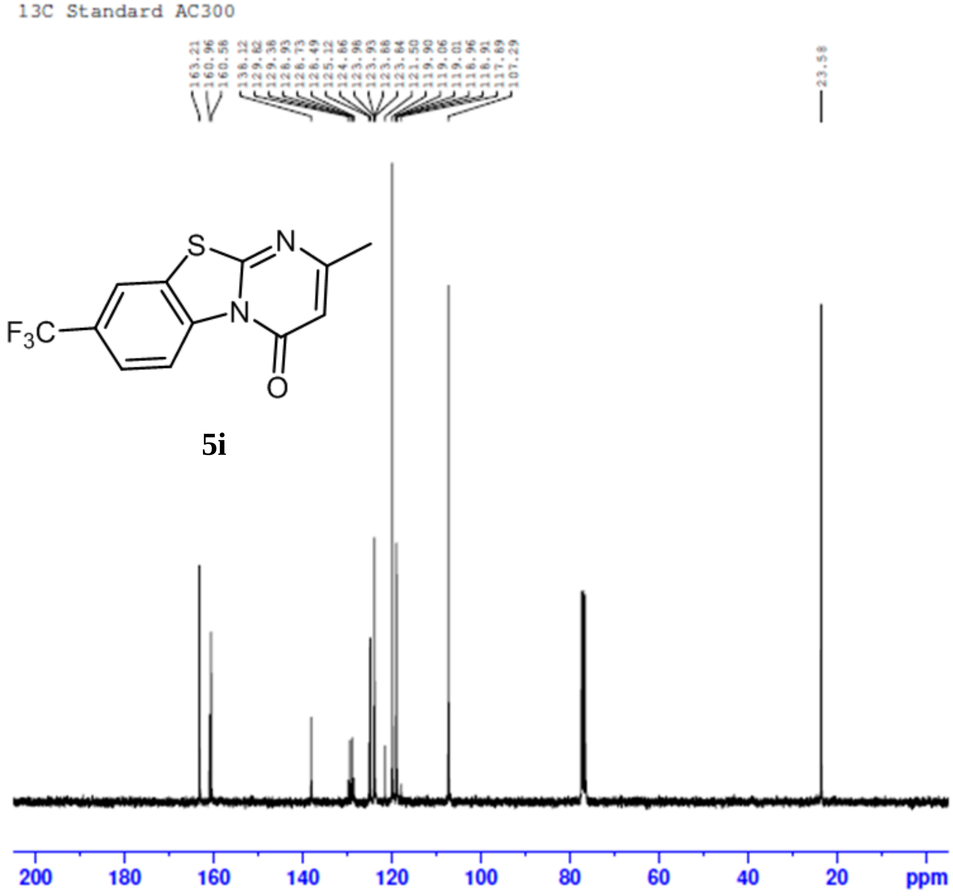

13C Standard AC300
163.21
160.96
160.58
138.12
129.62
129.36
107.29
23.58
F3C
S
N
O
200 180 160 140 120 100 80 60 40 20 ppm

5i

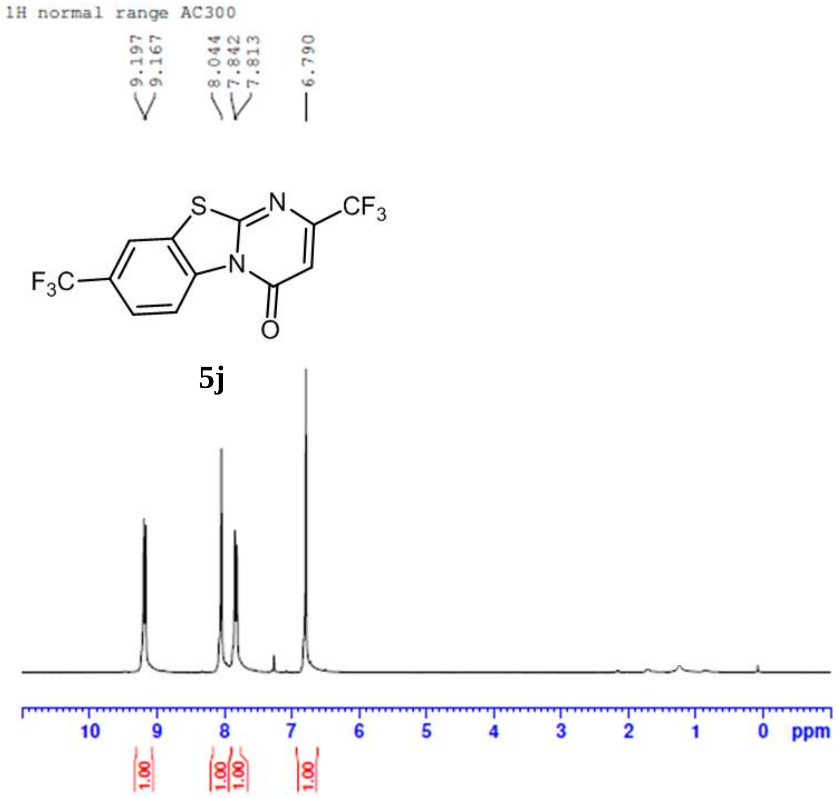
1H normal range AC300
9.197
9.167
8.044
7.842
7.813
6.790
S
N
CF3
F3C
N
O
5j
10
9
8
7
6
5
4
3
2
1
0
ppm
1.00
1.00
1.00
1.00

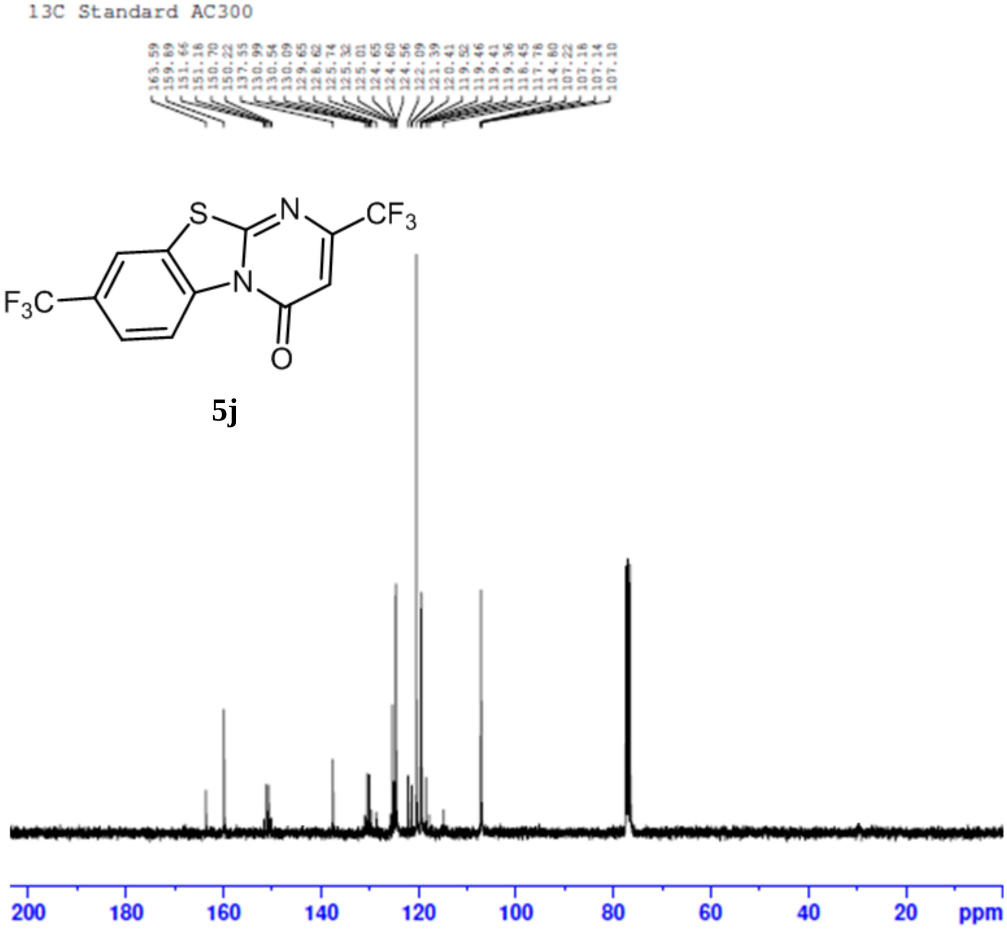
13C Standard AC300
163.59
159.89
151.66
151.16
150.70
150.22
137.55
130.99
130.54
130.09
129.65
128.62
125.74
125.32
125.01
124.65
124.60
124.56
122.09
121.39
120.41
119.52
119.46
119.41
119.36
118.45
117.76
114.80
107.22
107.16
107.14
107.10
S
N
CF3
F3C
N
O
5j
200
180
160
140
120
100
80
60
40
20
ppm

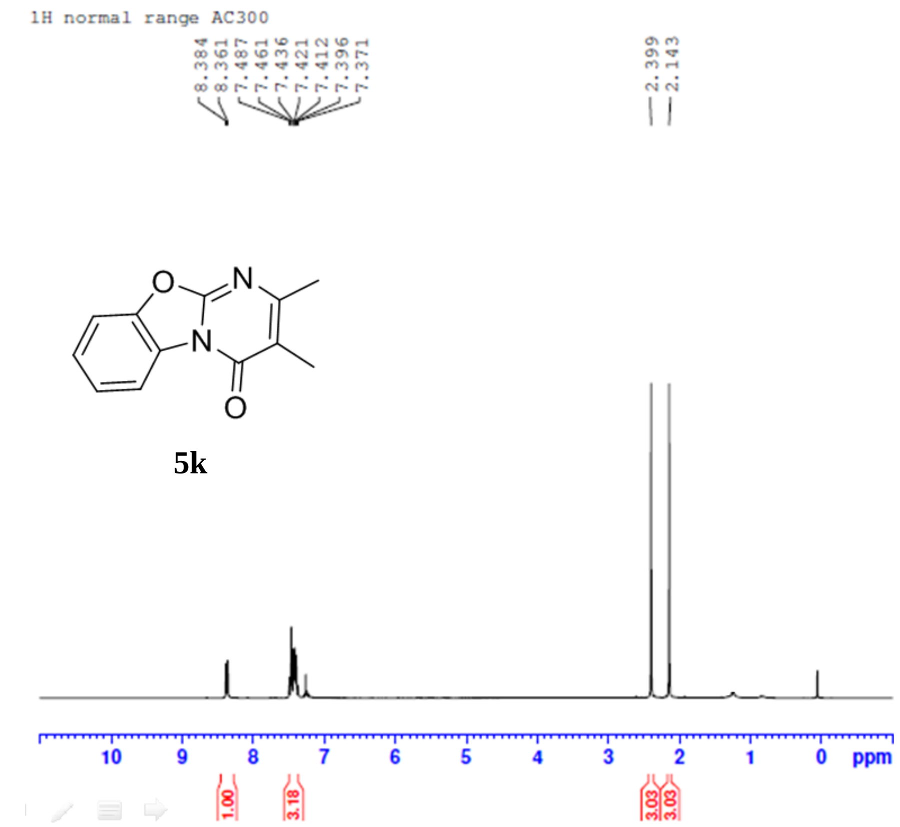
1H normal range AC300
8.384
8.361
7.487
7.461
7.436
7.421
7.412
7.396
7.371
2.399
2.143
5k
10 9 8 7 6 5 4 3 2 1 0 ppm
1.00
3.18
3.03
3.03

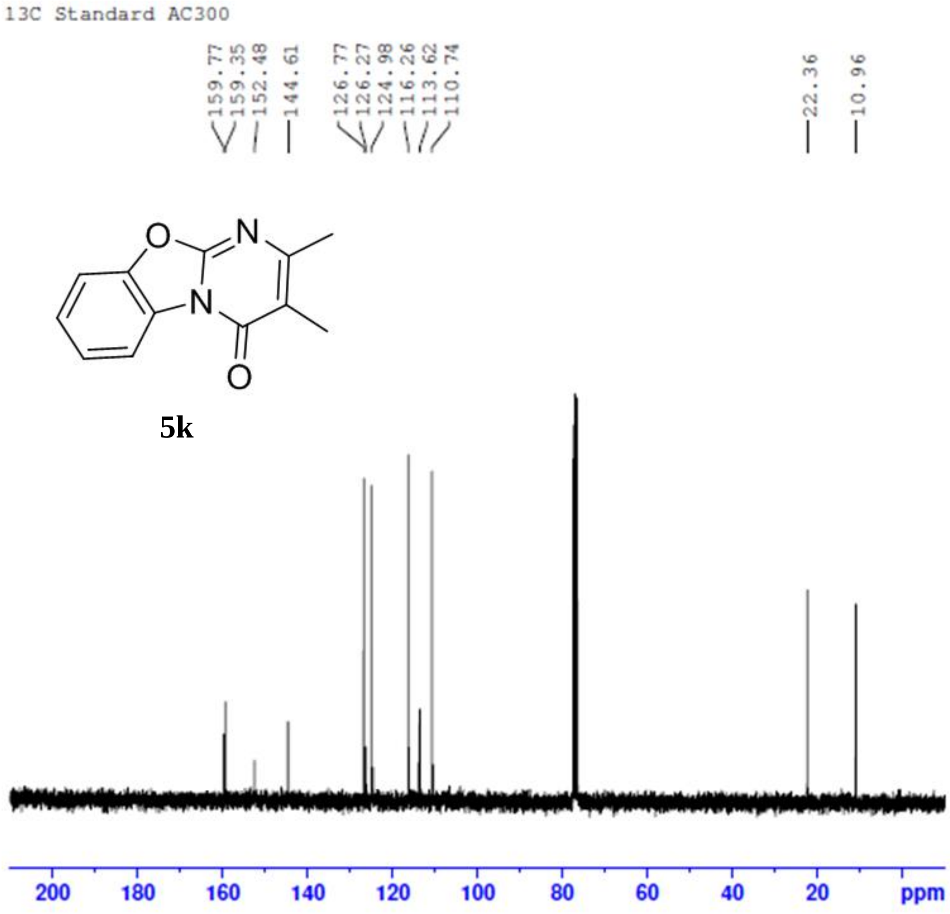
13C Standard AC300
159.77
159.35
152.48
144.61
126.77
126.27
124.98
116.26
113.62
110.74
22.36
10.96
5k
200 180 160 140 120 100 80 60 40 20 ppm

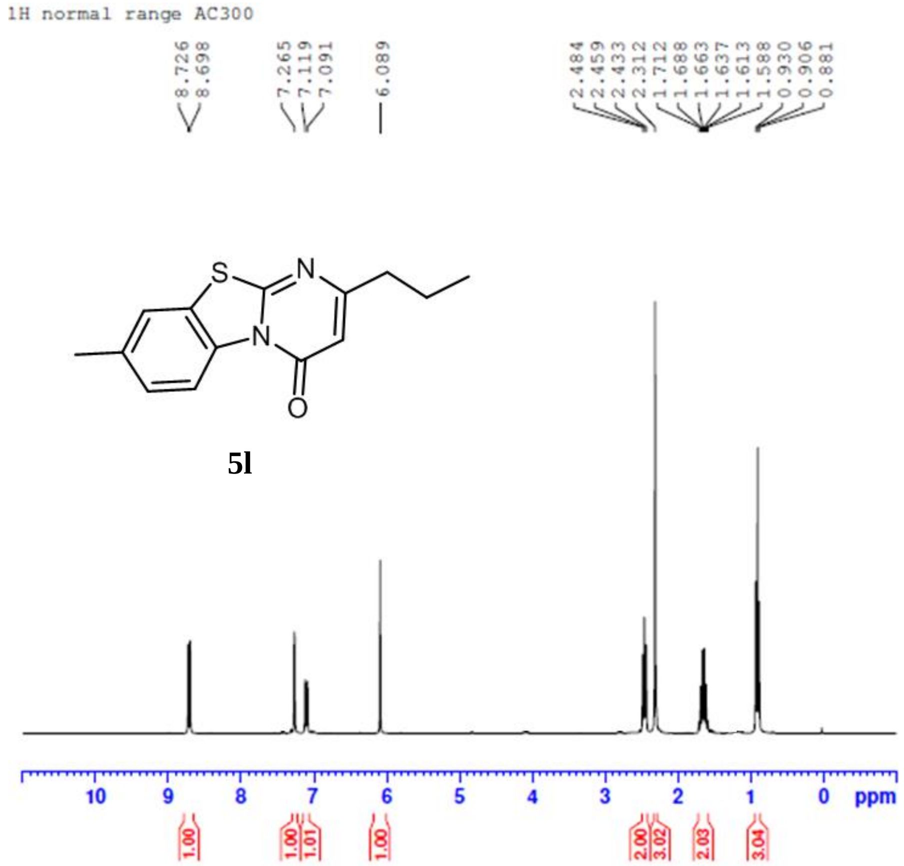
1H normal range AC300
8.726
8.698
7.265
7.119
7.091
6.089
2.484
2.459
2.433
2.312
1.712
1.688
1.663
1.637
1.613
1.588
0.930
0.906
0.881
S
N
N
O
5l
10
9
8
7
6
5
4
3
2
1
0
ppm
1.00
1.00
1.01
1.00
2.00
3.02
2.03
3.04

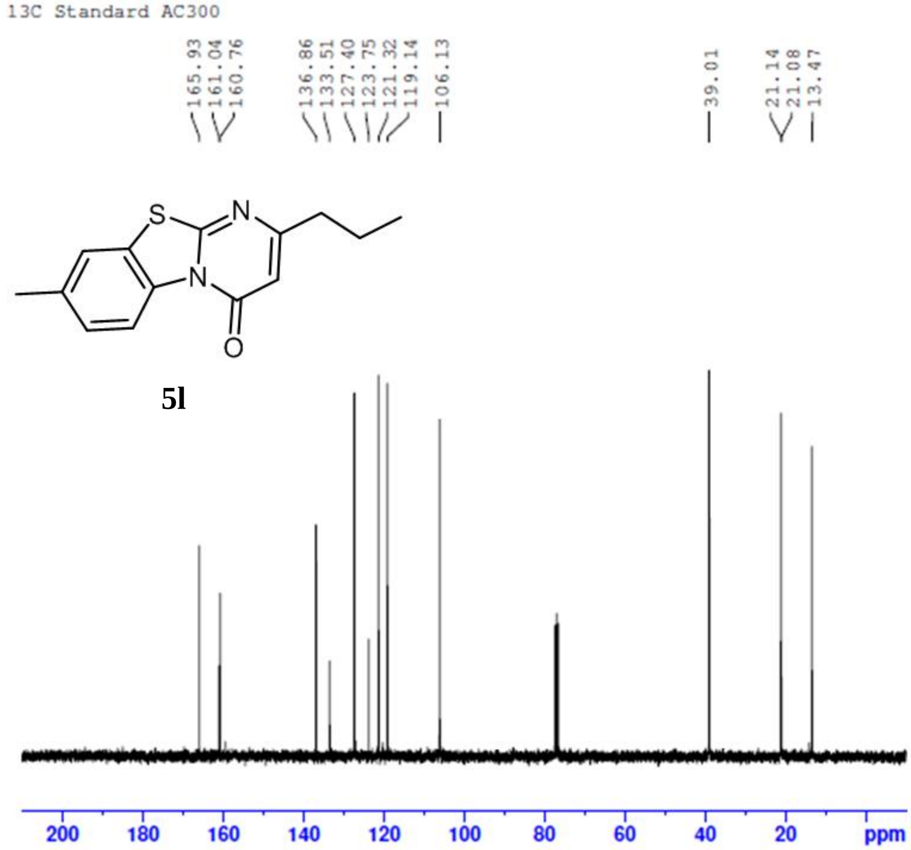
13C Standard AC300
165.93
161.04
160.76
136.86
133.51
127.40
123.75
121.32
119.14
106.13
39.01
21.14
21.08
13.47
S
N
N
O
5l
200
180
160
140
120
100
80
60
40
20
ppm

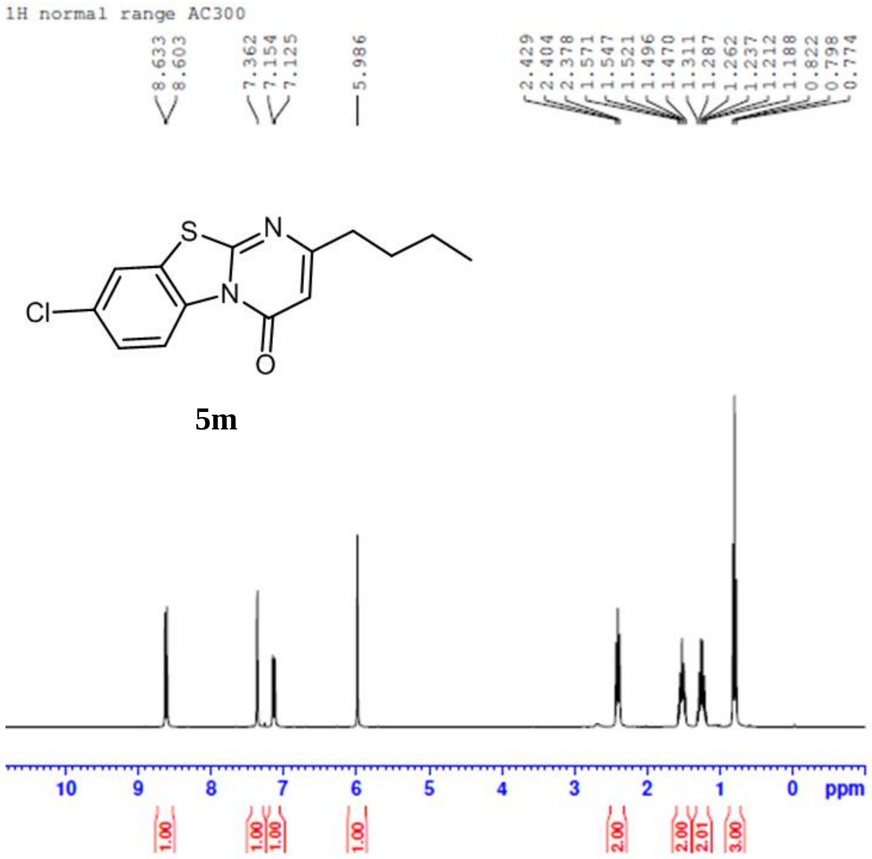
1H normal range AC300
8.633
8.603
7.362
7.154
7.125
5.986
2.429
2.404
2.378
1.571
1.547
1.521
1.496
1.470
1.311
1.287
1.262
1.237
1.212
1.188
0.822
0.798
0.774
5m
10 9 8 7 6 5 4 3 2 1 0 ppm
1.00
1.00
1.00
1.00
2.00
2.00
2.01
3.00

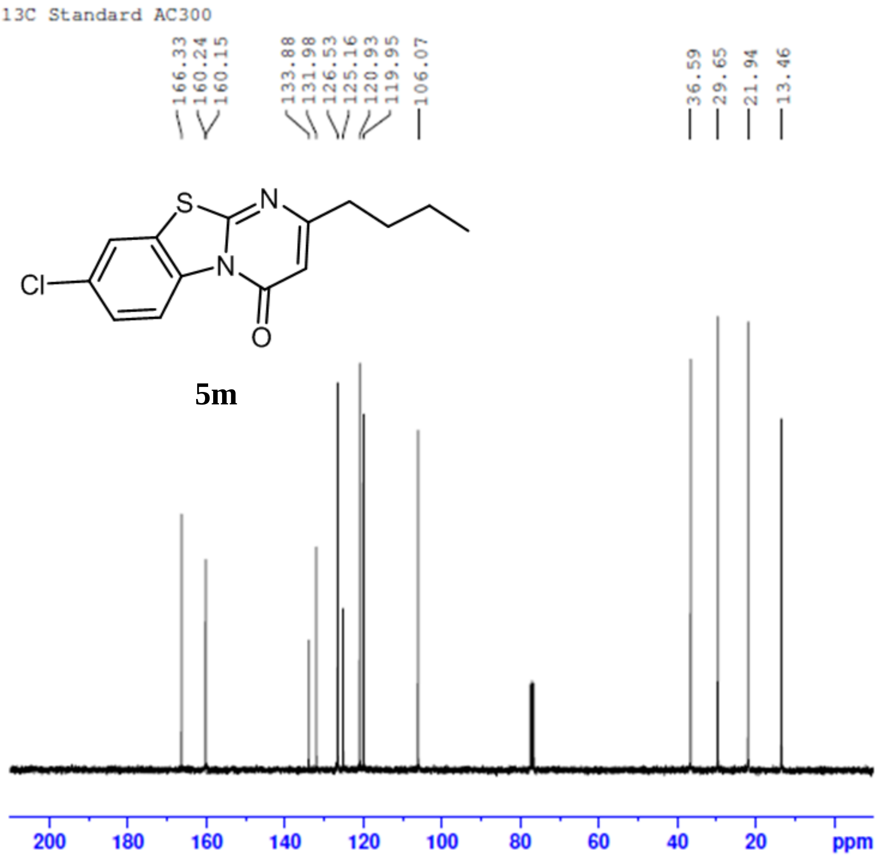
13C Standard AC300
166.33
160.24
160.15
133.88
131.98
126.53
125.16
120.93
119.95
106.07
36.59
29.65
21.94
13.46
5m
200 180 160 140 120 100 80 60 40 20 ppm

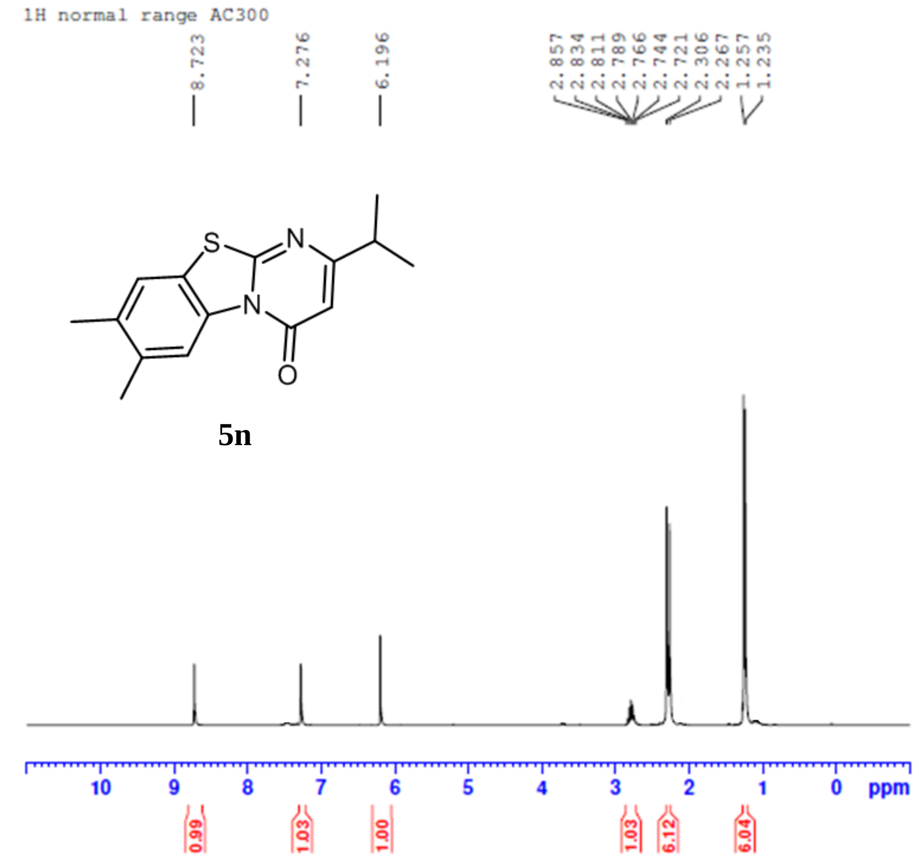

1H normal range AC300
8.723
7.276
6.196
2.857
2.834
2.811
2.789
2.766
2.744
2.721
2.306
2.267
1.257
1.235
S
N
N
O
5n
10
9
8
7
6
5
4
3
2
1
0
ppm
0.99
1.03
1.00
1.03
6.12
6.04

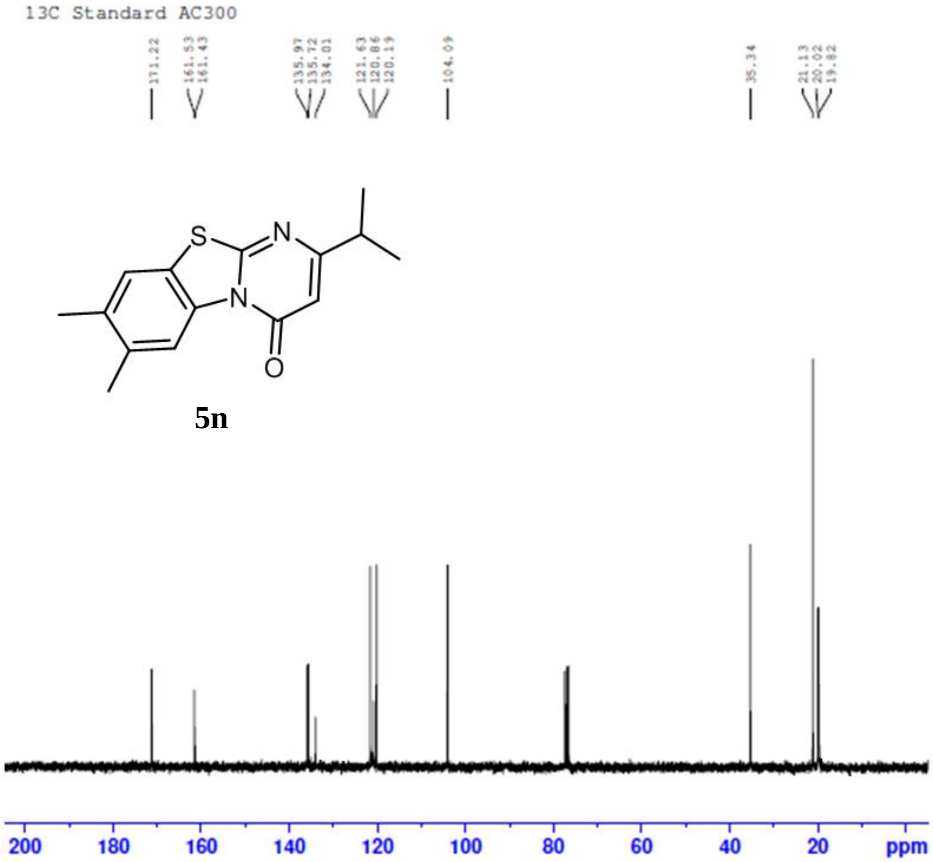

13C Standard AC300
171.22
161.53
161.43
135.97
135.72
134.01
121.63
120.86
120.19
104.09
35.34
21.13
20.02
19.82
S
N
N
O
5n
200
180
160
140
120
100
80
60
40
20
ppm

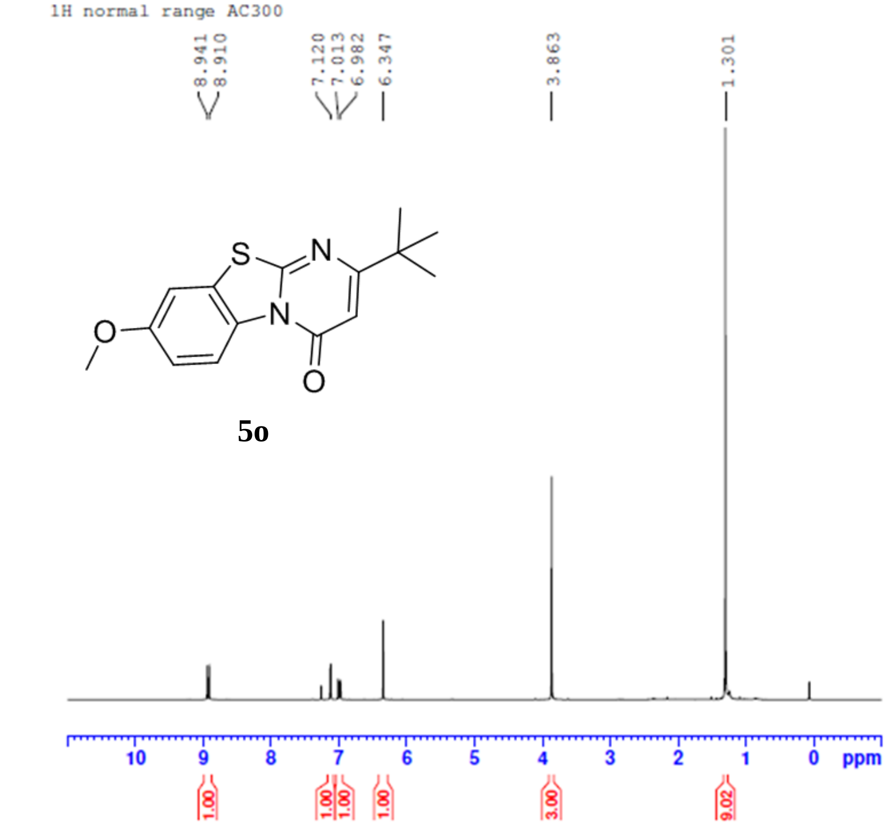

1H normal range AC300
8.941
8.910
7.120
7.013
6.982
6.347
3.863
1.301
S
N
N
O
O
5o
10
9
8
7
6
5
4
3
2
1
0
ppm
1.00
1.00
1.00
1.00
3.00
9.02

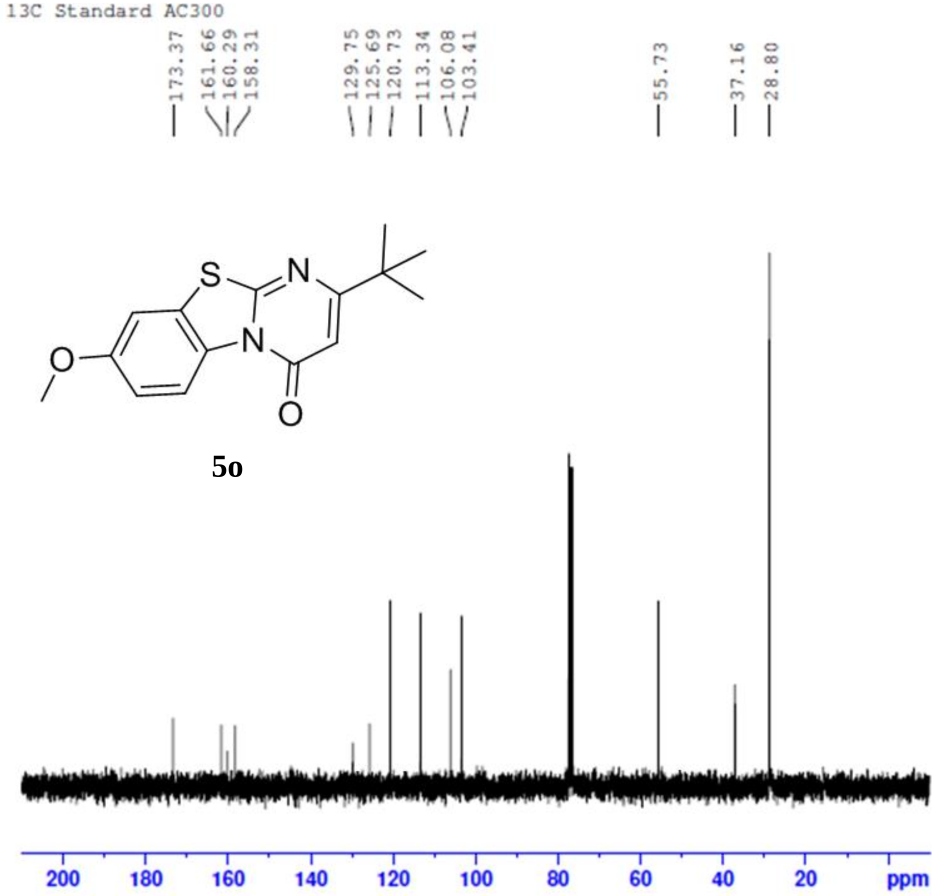

13C Standard AC300
173.37
161.66
160.29
158.31
129.75
125.69
120.73
113.34
106.08
103.41
55.73
37.16
28.80
S
N
N
O
O
5o
200
180
160
140
120
100
80
60
40
20
ppm

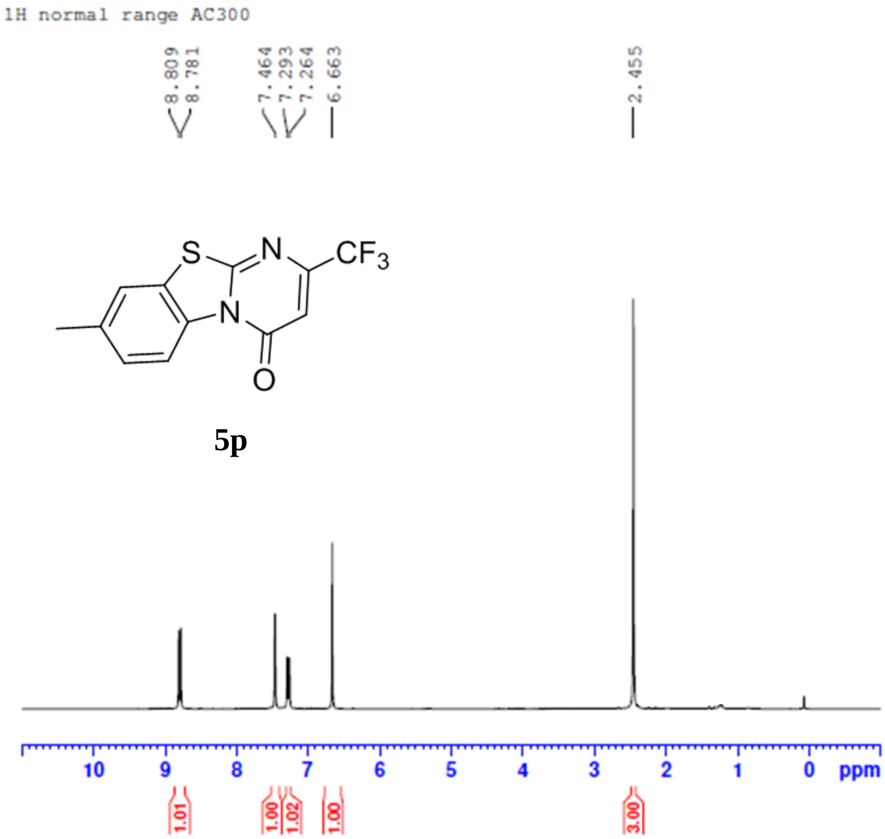
1H normal range AC300
8.809
8.781
7.464
7.293
7.264
6.663
2.455
CF3
S
N
O
5p
10
9
8
7
6
5
4
3
2
1
0
ppm
1.01
1.00
1.02
1.00
3.00

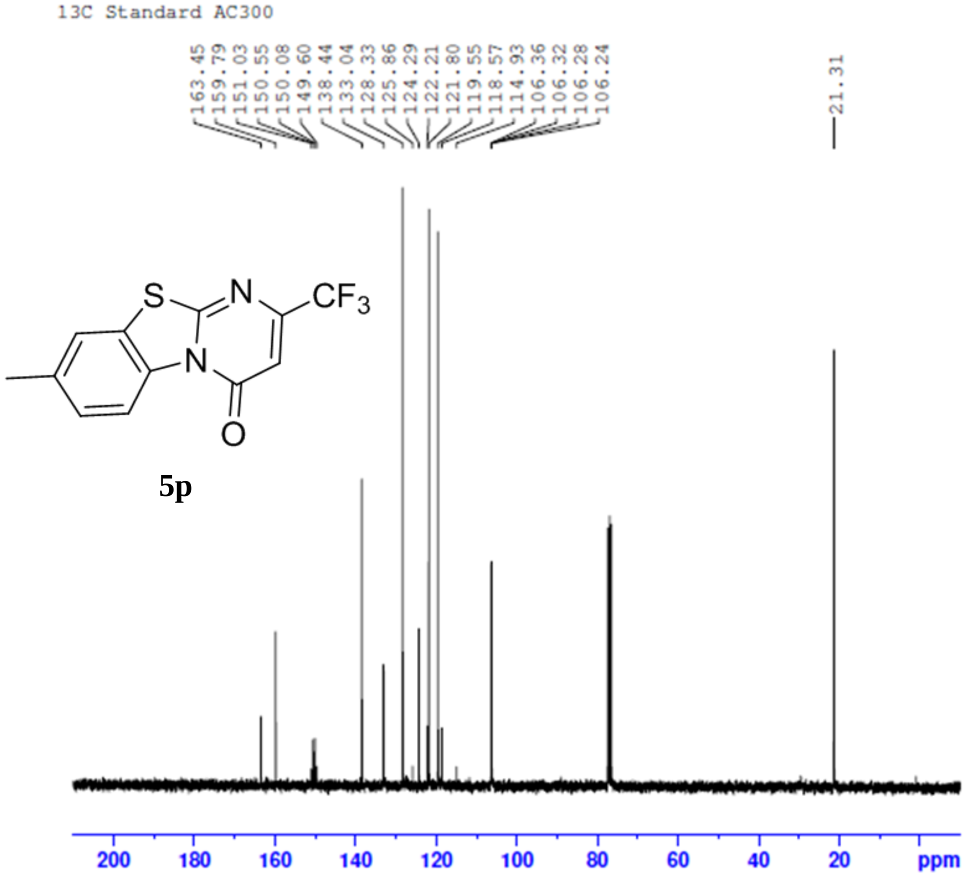
13C Standard AC300
163.45
159.79
151.03
150.55
150.08
149.60
138.44
133.04
128.33
125.86
124.29
122.21
121.80
119.55
118.57
114.93
106.36
106.32
106.28
106.24
21.31
CF3
S
N
O
5p
200
180
160
140
120
100
80
60
40
20
ppm

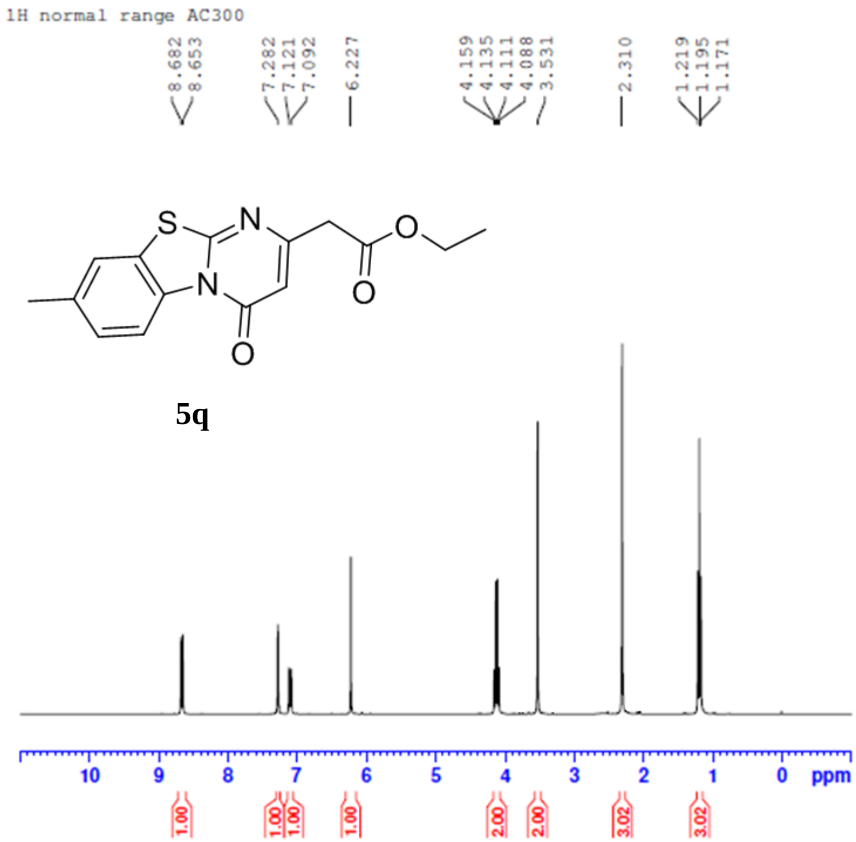
1H normal range AC300
8.682
8.653
7.282
7.121
7.092
6.227
4.159
4.135
4.111
4.088
3.531
2.310
1.219
1.195
1.171
S
N
N
O
O
O
ppm
1.00
1.00
1.00
1.00
2.00
2.00
3.02
3.02

5q

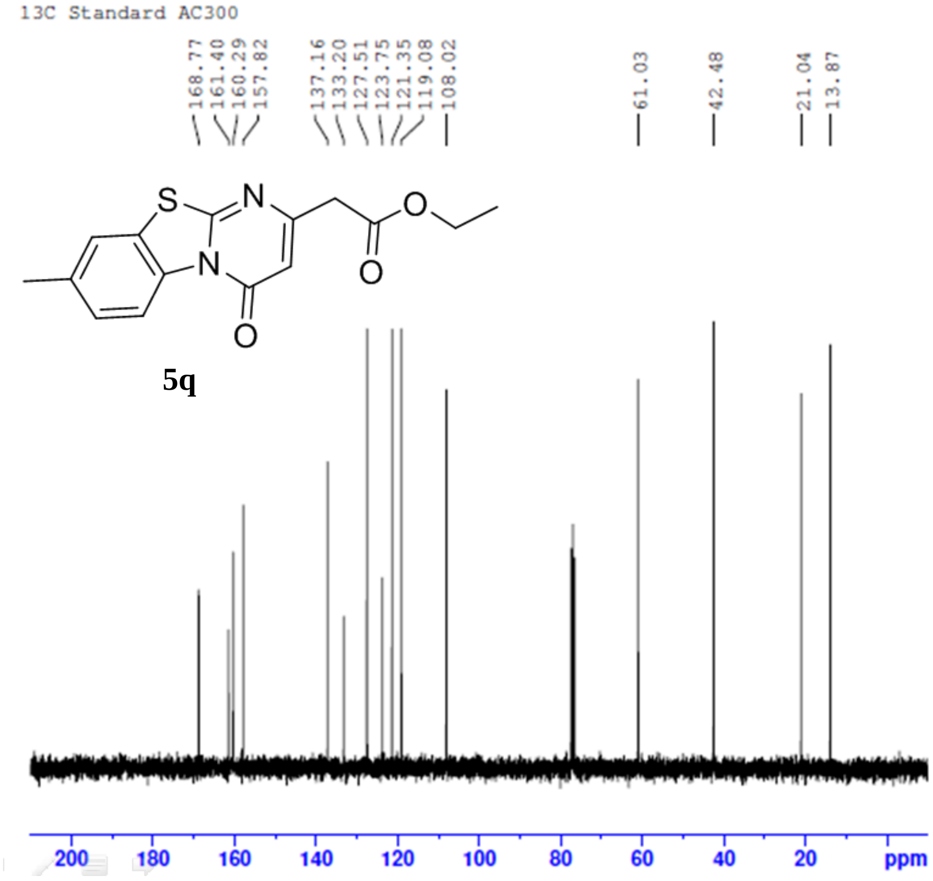
13C Standard AC300
168.77
161.40
160.29
157.82
137.16
133.20
127.51
123.75
121.35
119.08
108.02
61.03
42.48
21.04
13.87
S
N
N
O
O
O
ppm

5q

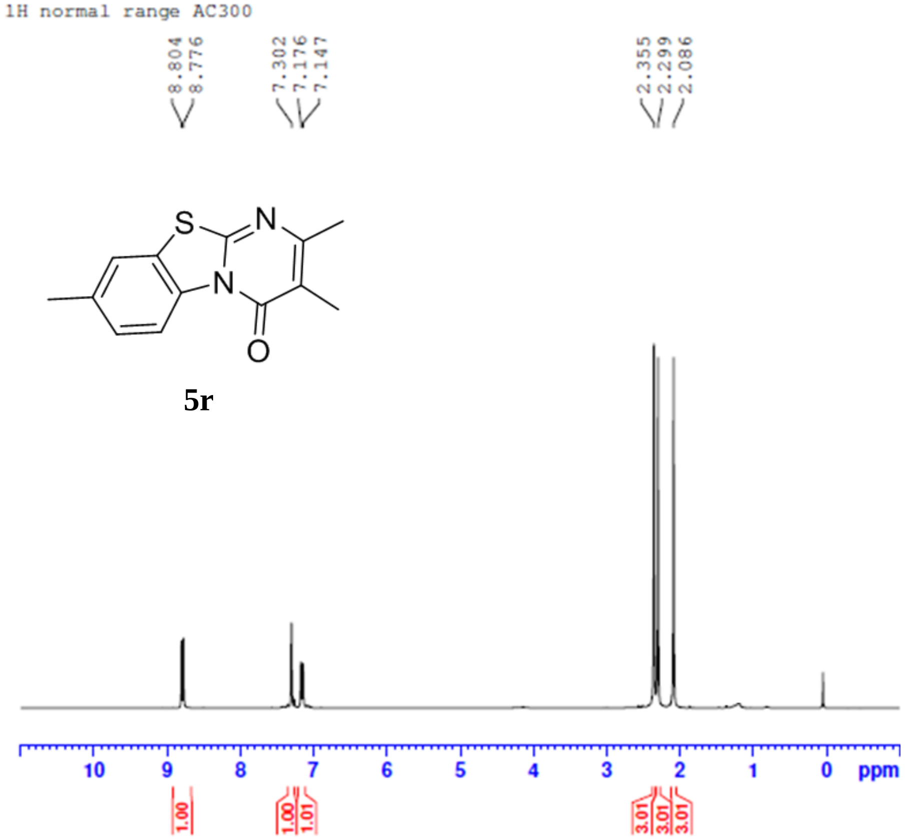
1H normal range AC300
8.804
8.776
7.302
7.176
7.147
2.355
2.299
2.086
S
N
N
O
5r
10
9
8
7
6
5
4
3
2
1
0
ppm
1.00
1.00
1.01
3.01
3.01
3.01

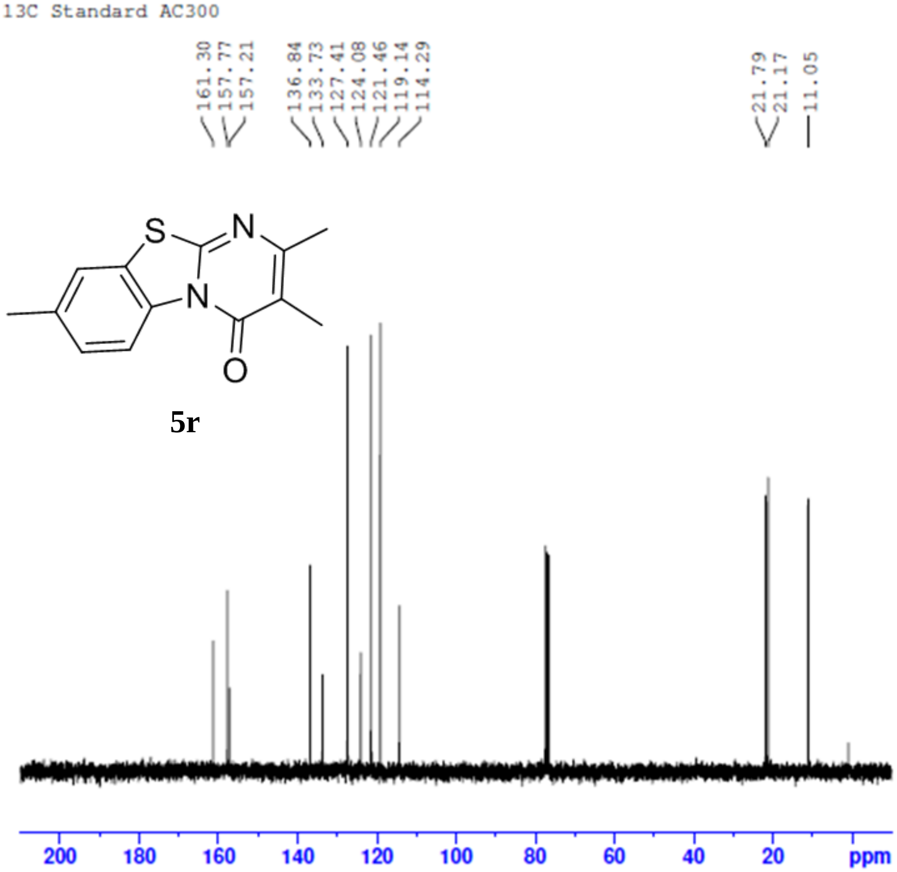
13C Standard AC300
161.30
157.77
157.21
136.84
133.73
127.41
124.08
121.46
119.14
114.29
21.79
21.17
11.05
S
N
N
O
5r
200
180
160
140
120
100
80
60
40
20
ppm

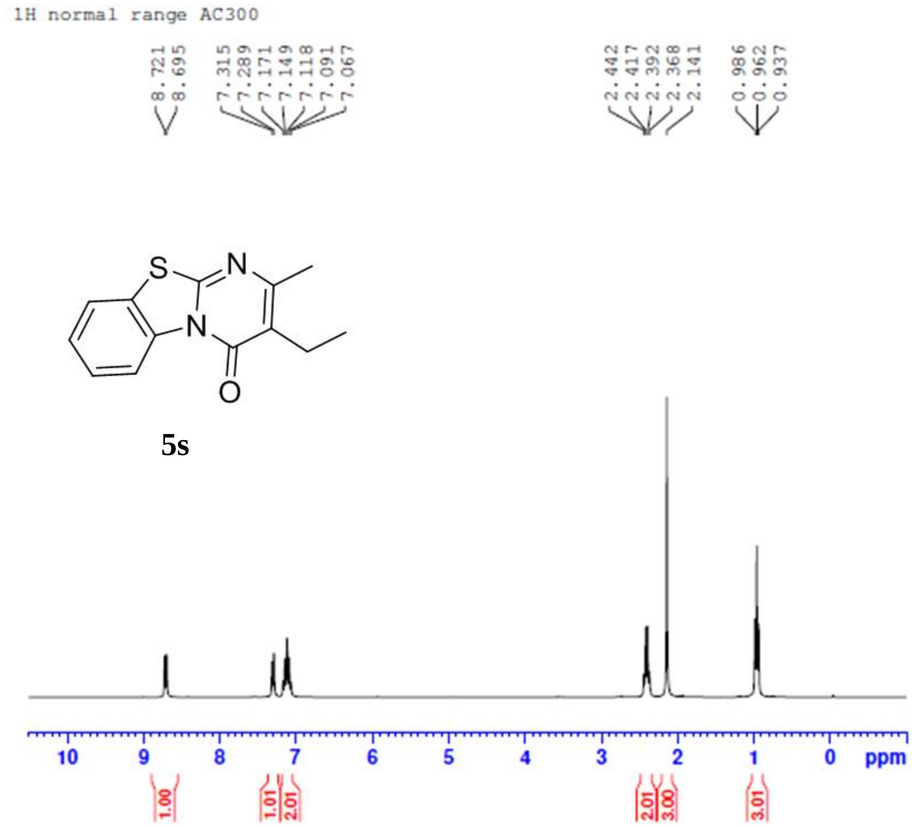
1H normal range AC300
8.721
8.695
7.315
7.289
7.171
7.149
7.118
7.091
7.067
2.442
2.417
2.392
2.368
2.141
0.986
0.962
0.937
S
N
N
O
5s
10 9 8 7 6 5 4 3 2 1 0 ppm
1.00
1.01
2.01
2.01
3.00
3.01

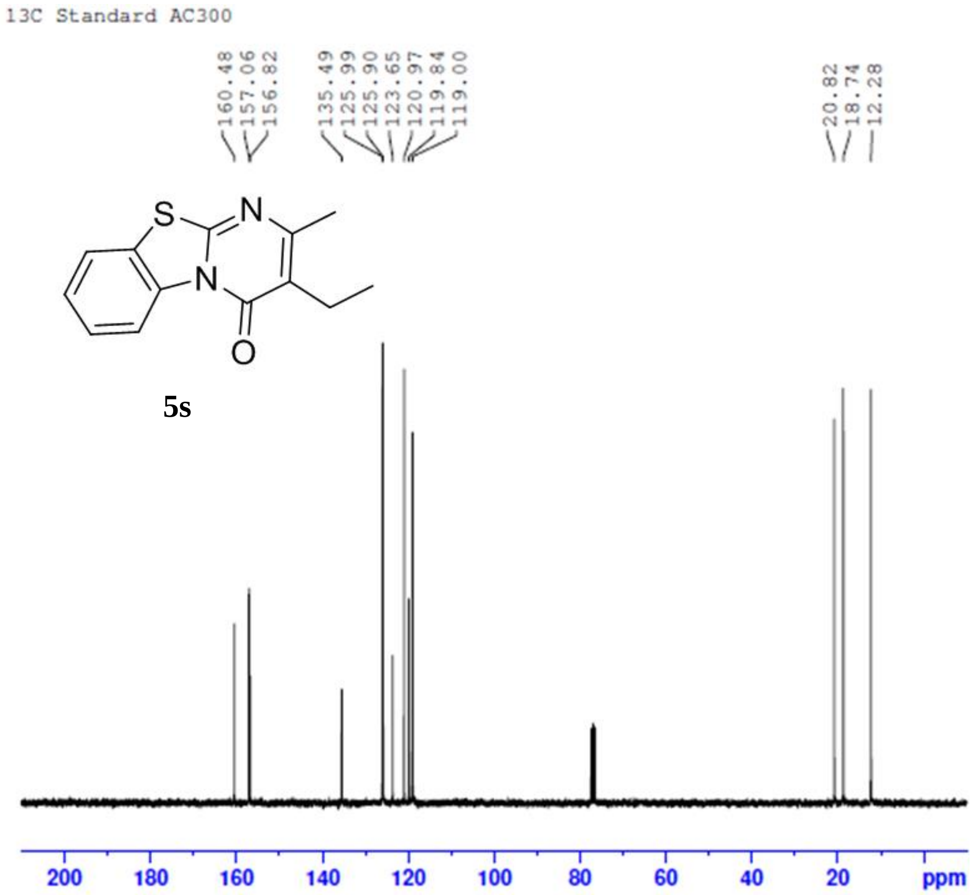
13C Standard AC300
160.48
157.06
156.82
135.49
125.99
125.90
123.65
120.97
119.84
119.00
20.82
18.74
12.28
S
N
N
O
5s
200 180 160 140 120 100 80 60 40 20 ppm

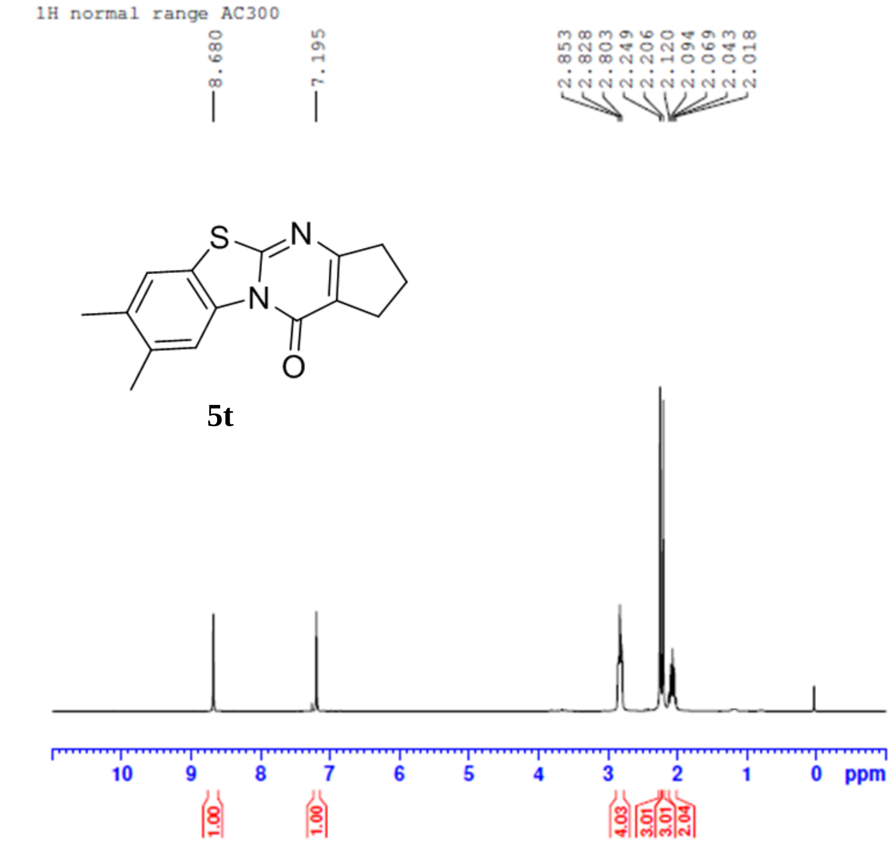
1H normal range AC300
8.680
7.195
2.853
2.828
2.803
2.249
2.206
2.120
2.094
2.069
2.043
2.018
S
N
N
O
5t
10 9 8 7 6 5 4 3 2 1 0 ppm
1.00
1.00
4.03
3.01
3.01
2.04

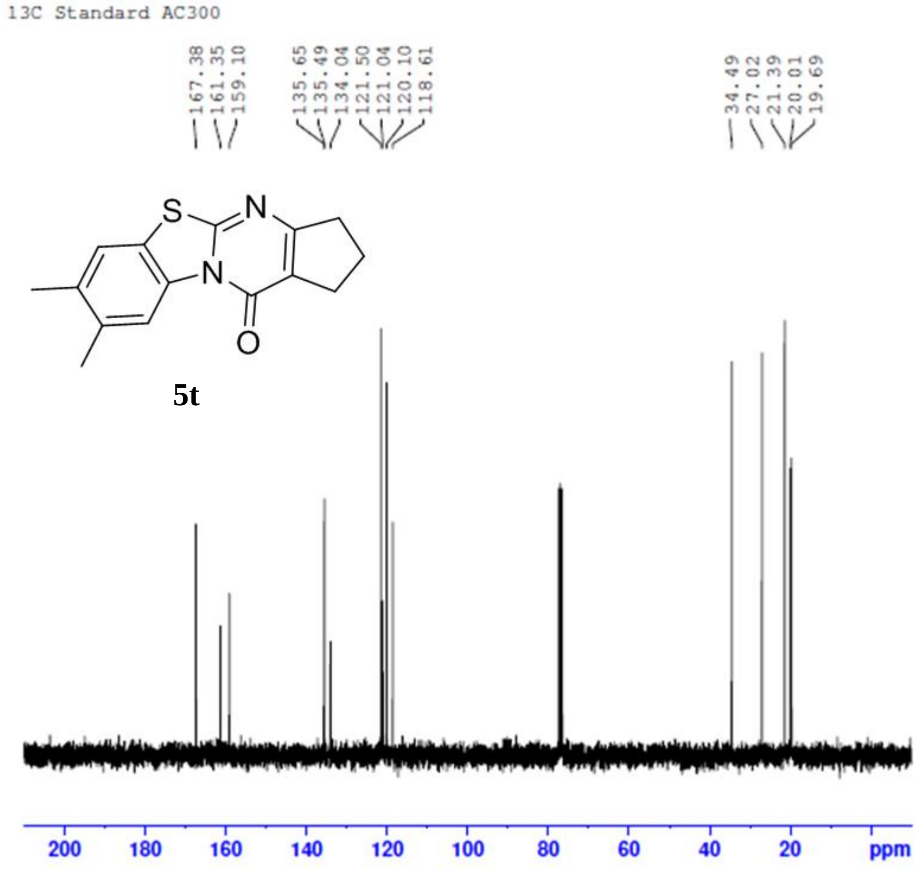
13C Standard AC300
167.38
161.35
159.10
135.65
135.49
134.04
121.50
121.04
120.10
118.61
34.49
27.02
21.39
20.01
19.69
S
N
N
O
5t
200 180 160 140 120 100 80 60 40 20 ppm

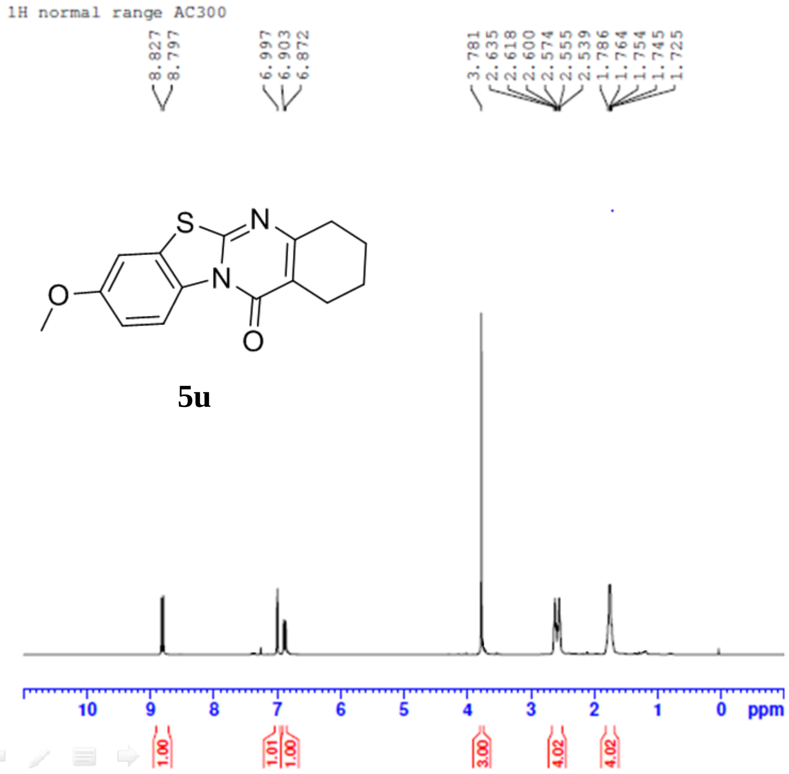
1H normal range AC300
8.827
8.797
6.997
6.903
6.872
3.781
2.635
2.618
2.600
2.574
2.555
2.539
1.786
1.764
1.754
1.745
1.725
S
N
N
O
O
5u
10
9
8
7
6
5
4
3
2
1
0
ppm
1.00
1.01
1.00
3.00
4.02
4.02

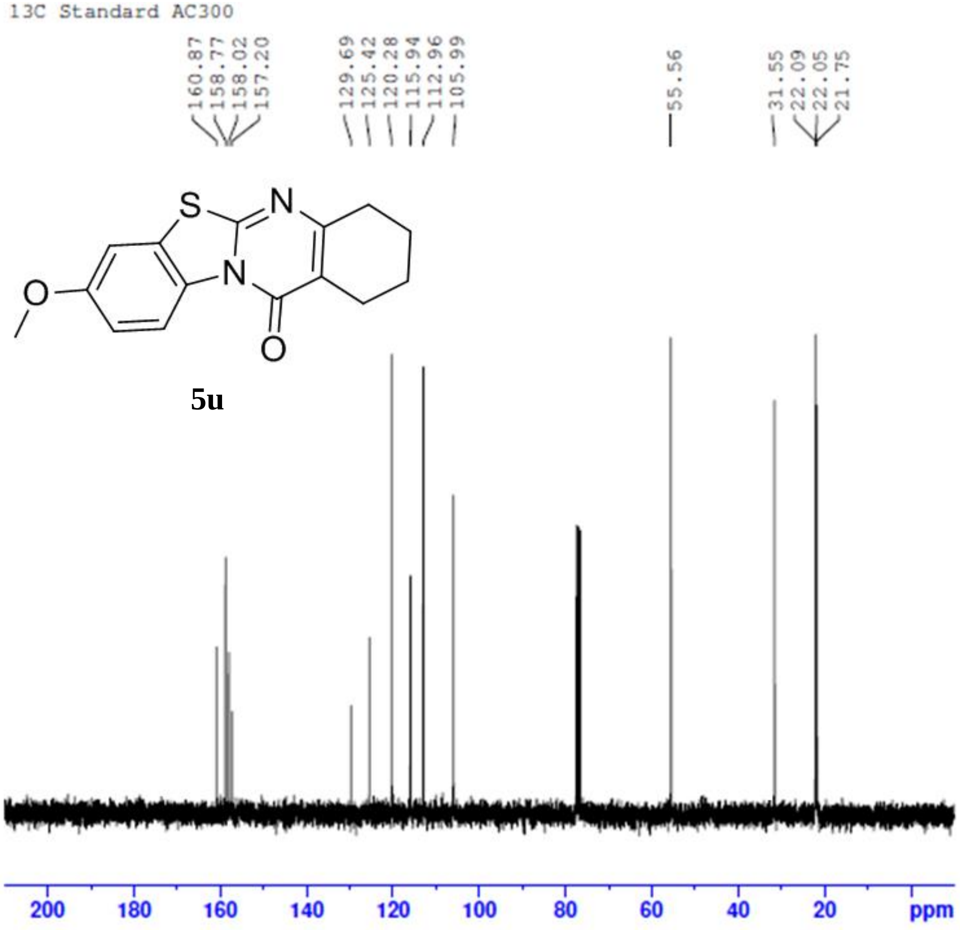
13C Standard AC300
160.87
158.77
158.02
157.20
129.69
125.42
120.28
115.94
112.96
105.99
55.56
31.55
22.09
22.05
21.75
S
N
N
O
O
5u
200
180
160
140
120
100
80
60
40
20
ppm

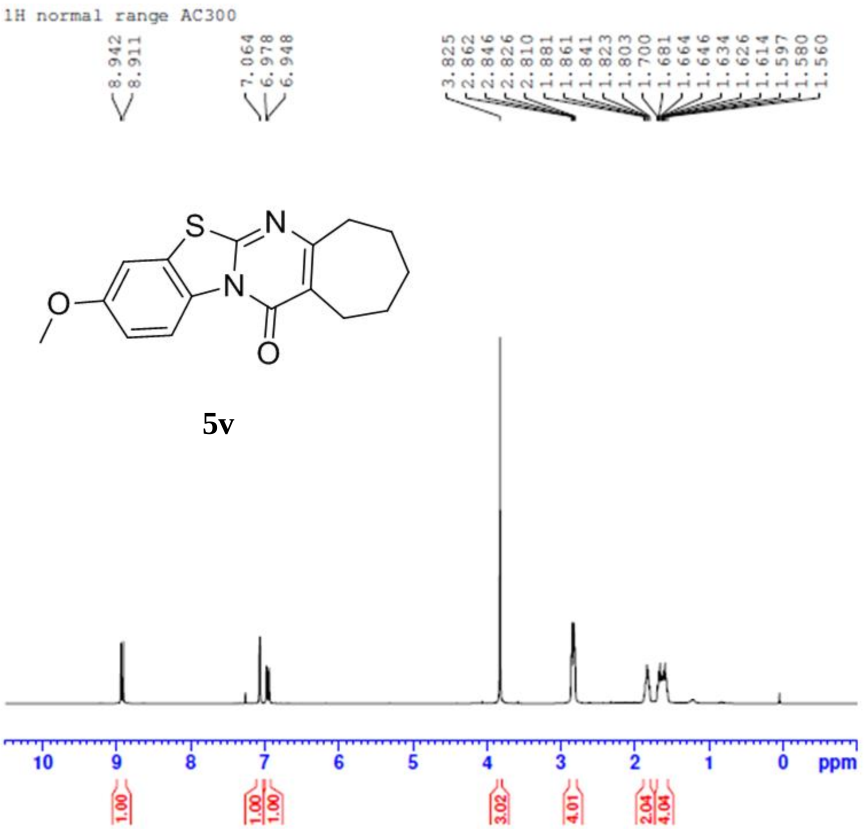
1H normal range AC300
8.942
8.911
7.064
6.978
6.948
3.825
2.862
2.846
2.826
2.810
1.881
1.861
1.841
1.823
1.803
1.700
1.681
1.664
1.646
1.634
1.626
1.614
1.597
1.580
1.560
5v
10 9 8 7 6 5 4 3 2 1 0 ppm
1.00
1.00
1.00
3.02
4.01
2.04
4.04

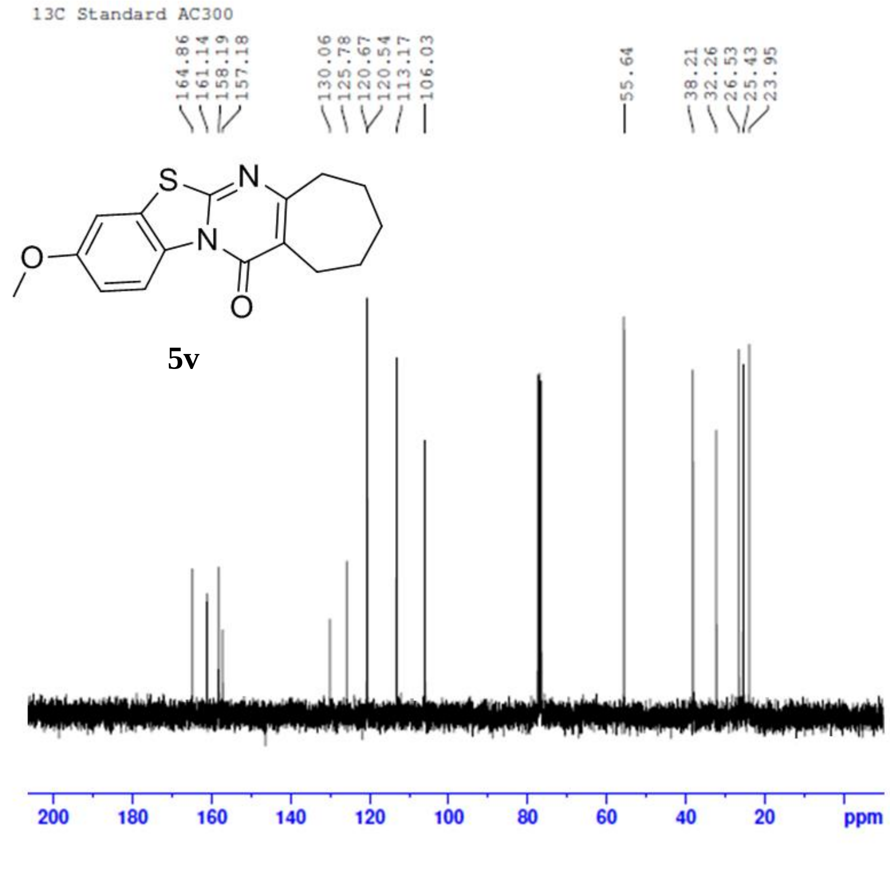
13C Standard AC300
164.86
161.14
158.19
157.18
130.06
125.78
120.67
120.54
113.17
106.03
55.64
38.21
32.26
26.53
25.43
23.95
5v
200 180 160 140 120 100 80 60 40 20 ppm

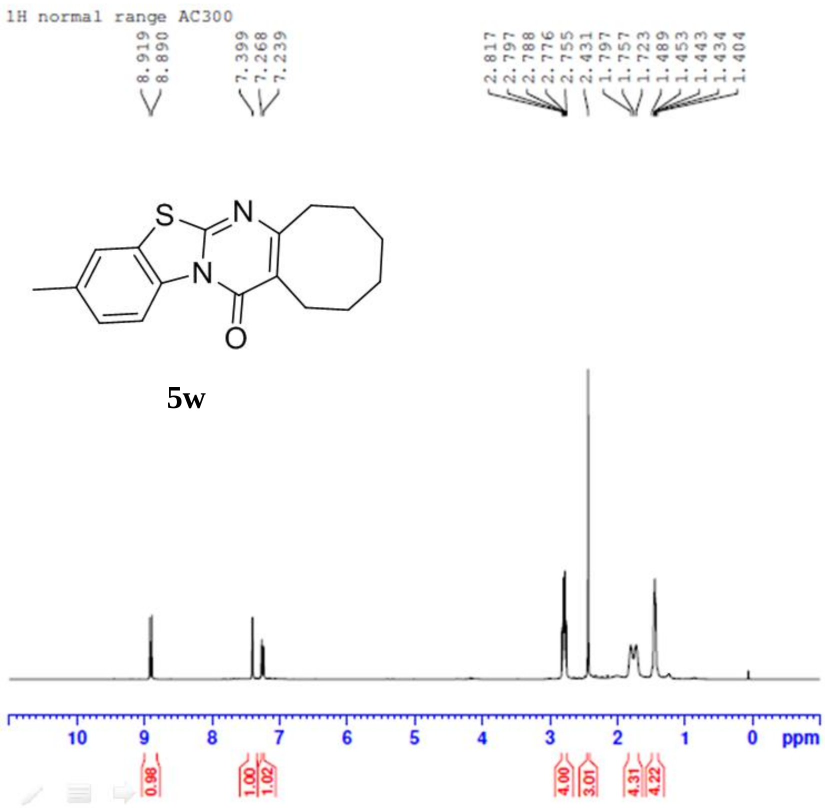
1H normal range AC300
8.919
8.890
7.399
7.268
7.239
2.817
2.797
2.788
2.776
2.755
2.431
1.797
1.757
1.723
1.489
1.453
1.443
1.434
1.404
S
N
N
O
10 9 8 7 6 5 4 3 2 1 0 ppm
0.98
1.00
1.02
4.00
3.01
4.31
4.22

5w

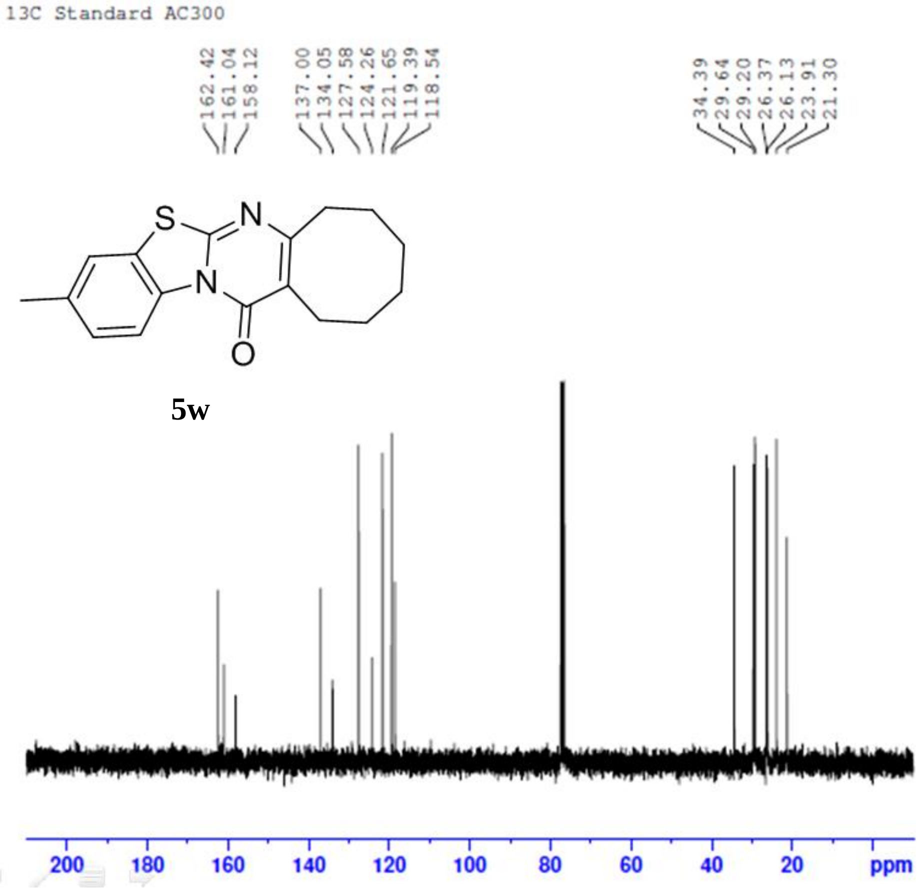
13C Standard AC300
162.42
161.04
158.12
137.00
134.05
127.58
124.26
121.65
119.39
118.54
34.39
29.64
29.20
26.37
26.13
23.91
21.30
S
N
N
O
200 180 160 140 120 100 80 60 40 20 ppm

5w

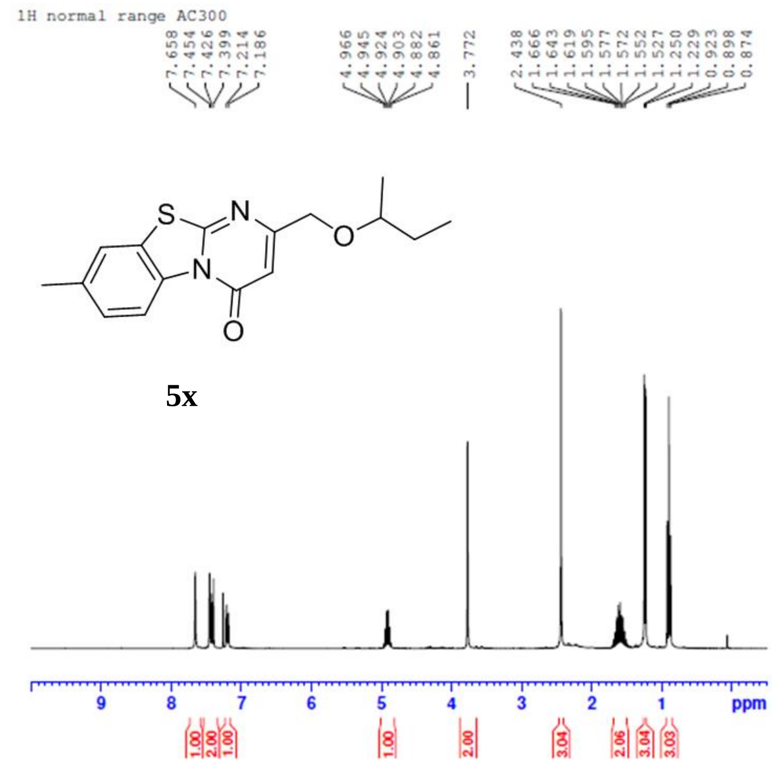
1H normal range AC300
7.658
7.454
7.426
7.399
7.214
7.186
4.966
4.945
4.924
4.903
4.882
4.861
3.772
2.438
1.666
1.643
1.619
1.595
1.577
1.572
1.552
1.527
1.250
1.229
0.923
0.898
0.874
S
N
N
O
O
5x
9
8
7
6
5
4
3
2
1
ppm
1.00
2.00
1.00
1.00
2.00
3.04
2.06
3.04
3.03

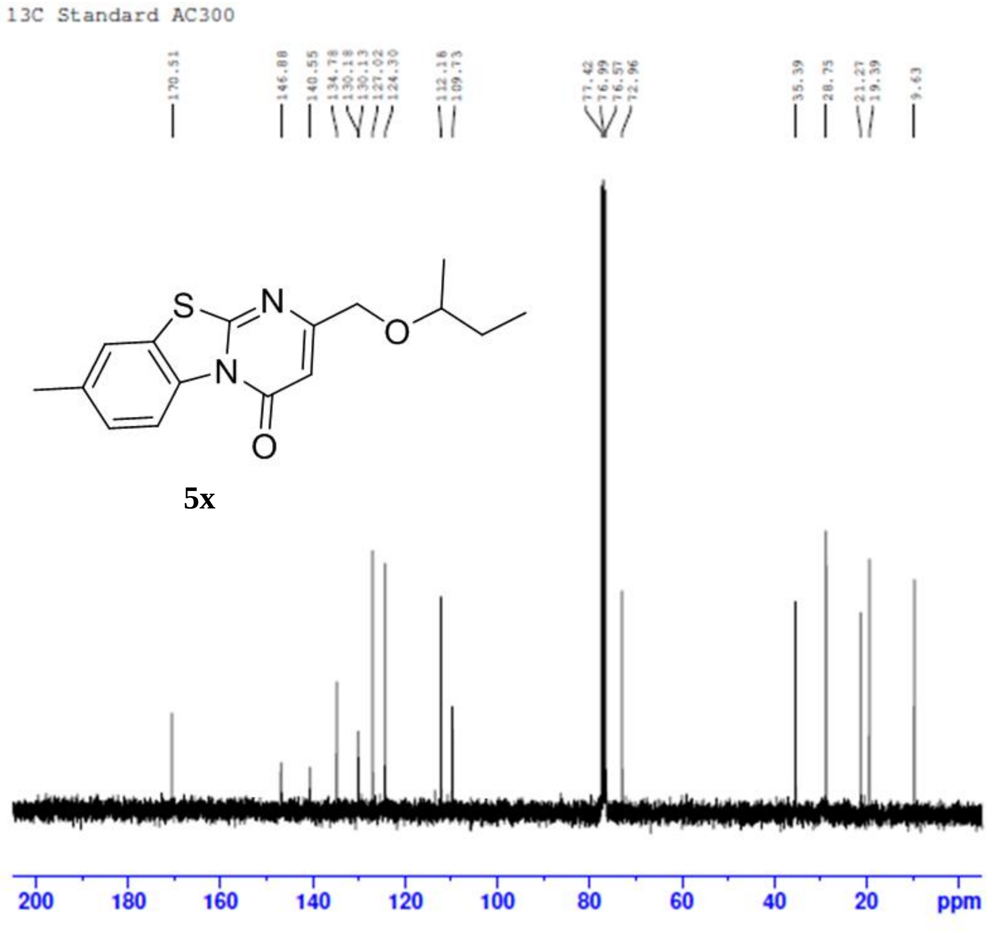
13C Standard AC300
170.51
146.88
140.55
134.78
130.18
130.13
127.02
124.30
112.18
109.73
77.42
76.99
76.57
72.96
35.39
28.75
21.27
19.39
9.63
S
N
N
O
O
5x
200
180
160
140
120
100
80
60
40
20
ppm

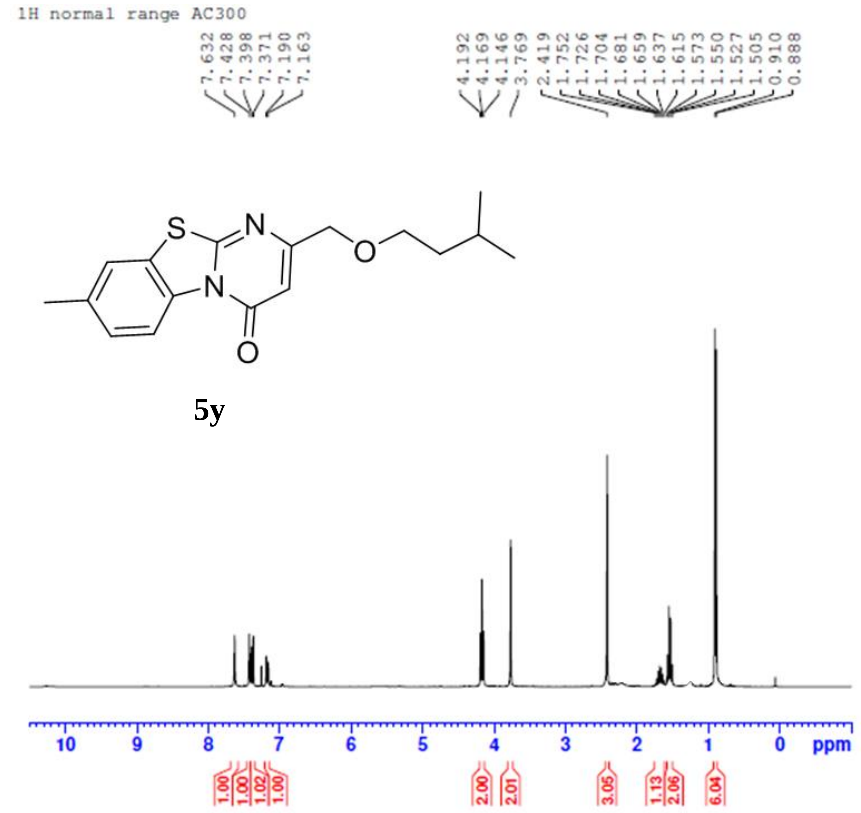
1H normal range AC300
7.632
7.428
7.398
7.371
7.190
7.163
4.192
4.169
4.146
3.769
2.419
1.752
1.726
1.704
1.681
1.659
1.637
1.615
1.573
1.550
1.527
1.505
0.910
0.888
S
N
N
O
O
5y
10 9 8 7 6 5 4 3 2 1 0 ppm
1.00
1.00
1.02
1.00
2.00
2.01
3.05
1.13
2.06
6.04

5y

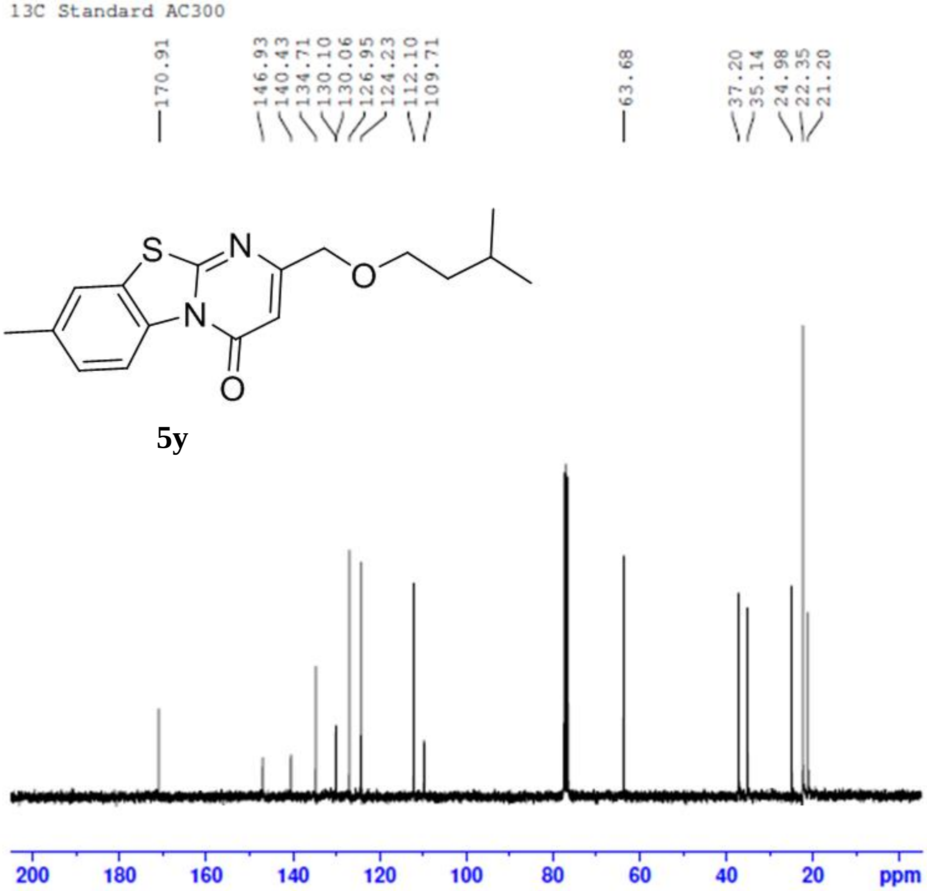
13C Standard AC300
170.91
146.93
140.43
134.71
130.10
130.06
126.95
124.23
112.10
109.71
63.68
37.20
35.14
24.98
22.35
21.20
S
N
N
O
O
5y
200 180 160 140 120 100 80 60 40 20 ppm

5y
